# Rapid and robust cysteine bioconjugation with vinylheteroarenes†

**DOI:** 10.1039/d1sc02722k

**Published:** 2021-06-07

**Authors:** Hikaru Seki, Stephen J. Walsh, Jonathan D. Bargh, Jeremy S. Parker, Jason Carroll, David R. Spring

**Affiliations:** Department of Chemistry, University of Cambridge Lensfield Road Cambridge CB2 1EW UK spring@ch.cam.ac.uk; Cancer Research UK Cambridge Institute, University of Cambridge Robinson Way Cambridge CB2 0RE UK; Early Chemical Development, Pharmaceutical Sciences, R&D, AstraZeneca Macclesfield UK

## Abstract

Methods for residue-selective and stable modification of canonical amino acids enable the installation of distinct functionality which can aid in the interrogation of biological processes or the generation of new therapeutic modalities. Herein, we report an extensive investigation of reactivity and stability profiles for a series of vinylheteroarene motifs. Studies on small molecule and protein substrates identified an optimum vinylheteroarene scaffold for selective cysteine modification. Utilisation of this lead linker to modify a number of protein substrates with various functionalities, including the synthesis of a homogeneous, stable and biologically active antibody–drug conjugate (ADC) was then achieved. The reagent was also efficient in labelling proteome-wide cysteines in cell lysates. The efficiency and selectivity of these reagents as well as the stability of the products makes them suitable for the generation of biotherapeutics or studies in chemical biology.

## Abstract 2

Vinylheteroarene linkers can chemoselectively modify cysteine residues in proteins and antibodies. These linkers give stable bioconjugates, and were used to synthesise efficacious antibody-drug conjugates.

## Graphical abstract

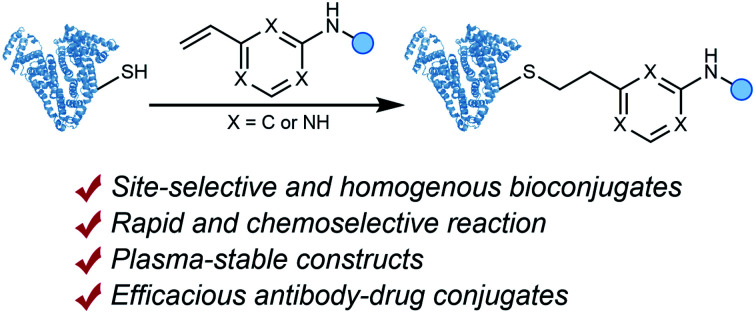

## Introduction

The chemical modification of proteins has a crucial role in enabling the interrogation of biology and providing improved medication. However, the controlled modification of these highly complex macromolecules remains challenging. To preserve the native structure and function of meta-stable proteins, mild reaction conditions are required.^1,2^ Furthermore, site-selective modification is often desirable, meaning that the transformation must be chemo- and regioselective for a target residue. Despite these challenges, a toolbox of reagents has been developed for the modification of proteinogenic amino acids: lysine,^3^ cysteine, tryptophan,^4^ methionine,^5,6^ tyrosine,^7,8^ histidine^9^ and the N-^10,11^ and C-terminus^12^ can now be functionalised with varying levels of success.

Cysteine modification is particularly attractive due to its high nucleophilicity under biocompatible conditions, solvent accessibility, and low natural abundance. In addition to the well-established α-halo acetamides,^13–15^ several cysteine-modification methodologies have been reported in recent years which selectively modify cysteine residues in a range of protein substrates. These reagents include bromomaleimides,^16^ bromopyridazinediones,^17,18^ unsaturated carbonyls,^19–21^ vinylsulfones,^22–25^ vinyl-pyridines^26–28^ and -pyridiniums,^29^ and unsaturated phosphonamidates;^30,31^ an excellent dedicated review has been published on cysteine modification reagents.^32^ Despite these new developments, in the field of biotherapeutic development, maleimide reagents remain most widely used due to their remarkable conjugation efficiency.^33^ For instance, antibody–drug conjugates (ADCs) are an emerging class of bioconjugate therapeutics, facilitating the targeted delivery of highly toxic payloads to target cells. ADCs comprise a tumour-specific monoclonal antibody attached to a potent cytotoxic drug *via* an appropriate linker. Indeed, six of the nine Food and Drug Administration (FDA)-approved ADCs are synthesised *via* maleimide modification of cysteine residues. Despite the utility of the maleimide linker, the product thiosuccinimide linkage has demonstrated poor stability caused by de-conjugation reactions. Instability of the protein–payload linkage can greatly affect the pharmacological properties of biotherapeutics such as ADCs, given that premature release of cytotoxic payloads can decrease its targeted delivery and cause off-target toxicity.^34,35^ To address this, modified maleimide reagents have been developed to increase the stability of the bioconjugate.^36,37^ One approach involves the use of self-hydrolysing maleimides which catalyse hydrolytic opening of the thiosuccinimide ring, thus reducing its susceptibility towards E_1_cB-type elimination.^38,39^

**Fig. 1:**
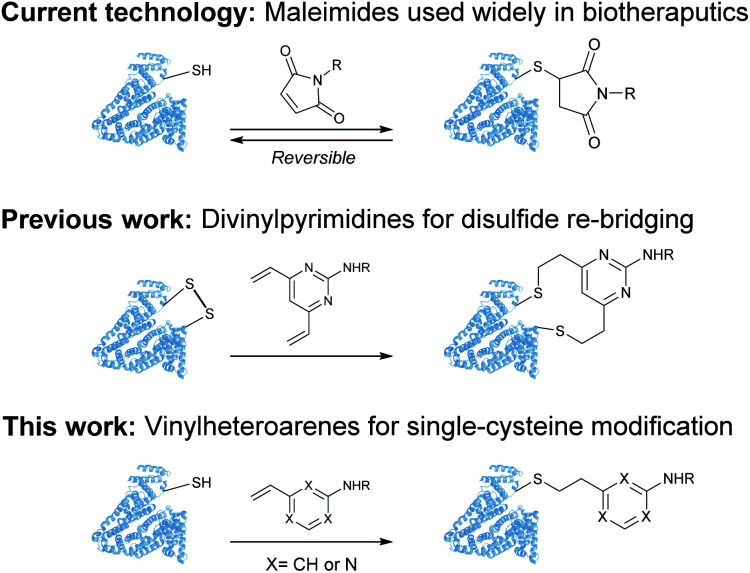
Use of maleimides, divinylpyrimidines and vinylheteroarenes for post-translational protein modification.

Despite these significant advances, new methods to predictively generate stable cysteine bioconjugates are still required. We have previously reported divinylpyrimidine and divinyltriazine reagents for cysteine cross-linking.^40–47^ While stable and functional modification of proteins or peptides could be achieved with these reagents, they are only useful when two proximal cysteine residues are present in the protein of interest. To expand the scope and generality of this scaffold, it was envisioned that monovinylheteroarene reagents would enable efficient and selective modification of single cysteine residues, regardless of spatial arrangement.^28^ Herein, a comprehensive comparison of a library of monovinylheteroarenes was conducted, culminating in the selective and stable modification of numerous protein substrates (Fig. 1).

## Results and discussion

### Reactivity of vinylheteroarenes towards biological nucleophiles

It was hypothesised that variable heteroarene ring electronics would greatly affect reactivity, with increasing electron deficiency increasing the rate of cysteine addition into the vinyl group. However, increased electrophilicity may also cause increased reactivity towards other proteinogenic nucleophiles (*e.g.* lysine, N-terminus). It was therefore deemed imperative to measure the rate of reaction of vinylheteroarenes with differing electronic characteristics with a range of proteinogenic nucleophiles. To begin investigations, vinyl-pyridine **1**, -pyrimidine **2**, -triazine **3** and -tetrazine **4** ^48,49^ were synthesised from commercially available starting materials (refer to ESI†); these model linkers all contain an amino functionality for further functionalisation.

Vinylheteroarenes **1**, **2**, **3**, and **4** (10 mM) were incubated with an equimolar quantity of Boc-Cys-OMe in a mixture of sodium phosphate (NaPi, pH 8, 50 mM in D_2_O) and CD_3_OD, and the reaction progress was monitored *via*^1^H NMR spectroscopy (ESI Section 2†). Kinetic analysis revealed that vinylpyrimidine **2** and vinyltriazine **3** were the most reactive, displaying second order rate constants of 0.375 M^−1^ s^−1^ and 3.10 M^−1^ s^−1^, respectively (Fig. S2 and S3†). Pleasingly, >95% conversion was observed in 20 minutes for **2** and 10 minutes for **3** when 10 mM vinylheteroarene solutions were incubated with stoichiometric Boc-Cys-OMe; this represents good kinetics for bioconjugation. For comparison, traceless Staudinger ligations and strain-promoted alkyne–azide cycloadditions have rate constants of only 10^−3^ M^−1^ s^−1^ and 10^−2^ M^−1^ s^−1^, respectively, and yet are widely used for protein modification.^50^ With this in mind, the reaction on vinylpyrimidine and vinyltriazine scaffolds was deemed sufficiently fast for cysteine modification. In contrast, the significantly lower measured rate constant for vinylpyridine **1** (4.91 × 10^−3^ M^−1^ s^−1^) was considered too low to be an effective bioconjugation method (Fig. S1†). Surprisingly, no reaction with the vinyl group was observed upon treatment of vinyltetrazine **4** with Boc-Cys-OMe. Instead, a second set of vinyl peaks were observed in the ^1^H NMR spectra (Fig. S7†), suggestive of direct nucleophilic attack on the tetrazine ring – a previously documented type of reactivity.^51,52^ As such, vinyltetrazines were also discounted from further studies.

Further investigations were conducted using **2** as a model substrate. Vinylpyrimidine **2** reacted with Boc-Cys-OMe over a range of biologically relevant pHs, with second order rate constants of 0.510, 0.636 and 0.851 M^−1^ s^−1^ at pH 7, 6 and 5, respectively (ESI Section 2.5†). Furthermore, when more dilute NaPi (pH 8; 50 mM, 25 mM or 10 mM) was used as the reaction medium, a reduction of the second order rate constants was observed (ESI Section 2.6†). These data suggest that vinylhetereoarenes react with cysteines *via* general base catalysis.

To ascertain the selectivity of these linkers for cysteines over other protein nucleophiles, vinylheteroarenes **1**, **2** and **3** were incubated with either Boc-Lys-OMe or H-Ala-NH_2_ (representative of the protein N-terminus) under the same conditions used in the reactions with Boc-Cys-OMe (*i.e.* 10 mM vinylheteroarene, 10 mM amino acid, NaPi (pH 8, 50 mM), CD_3_OD; refer to ESI Sections 2.3 and 2.4†). Pleasingly, no reactivity was observed between vinylpyridine **1** and vinylpyrimidine **2** with Boc-Lys-OMe or H-Ala-NH_2_ after monitoring overnight. Gratifyingly, the minimal reactivity that was observed between vinyltriazine **3** with the N-nucleophiles translates to >3200-fold selectivity for cysteine. This represents a significant improvement *versus* maleimides – the state-of-the-art linker for cysteine modification – which are known to react with both cysteine and lysine residues^53–55^ (Table 1).

**Table 1:** The second order rate constants for the reaction of vinylpyridine **1**, vinylpyrimidine **2**, vinyltriazine **3**, and vinyltetrazine **4** with Boc-Cys-OMe, Boc-Lys-OMe or H-Ala-NH_2_. Average of two replicates; error bars represent standard deviation of the mean

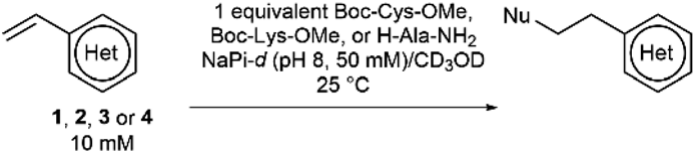			
Linker	Second order rate constants (*k*_2_), ×10^−3^ M^−1^ s^−1^		
	Boc-Cys-OMe	Boc-Lys-OMe	H-Ala-NH_2_
Vinylpyridine **1**	4.91 ± 0.01	0^a^	0^a^
Vinylpyrimidine **2**	375 ± 30	0^a^	0^a^
Vinyltriazine **3**	3100 ± 30	0.323 ± 0.006	0.944 ± 0.090
Vinyltetrazine **4**	n/a^b^	—	—
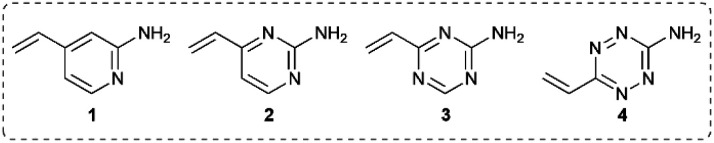			

a No reaction observed.

b Significant side reaction observed.

### Stability of vinylheteroarene–thiol conjugates

Given the favourable reaction rates and cysteine selectivity observed for both vinylpyrimidine and vinyltriazine scaffolds, our attention turned towards determining the stability of the corresponding linker–cysteine conjugates. It was proposed that using a fluorinated thiol would enable the use of ^19^F NMR to monitor the rate of decomposition, given the quantitative nature and high sensitivity of this technique. Thus, conjugates **5**, **6** and **7** were synthesised by reacting difluorobenzyl mercaptan with vinylheteroarenes **2** and **3**, and *N*-methylmaleimide, respectively.

**Fig. 2:**
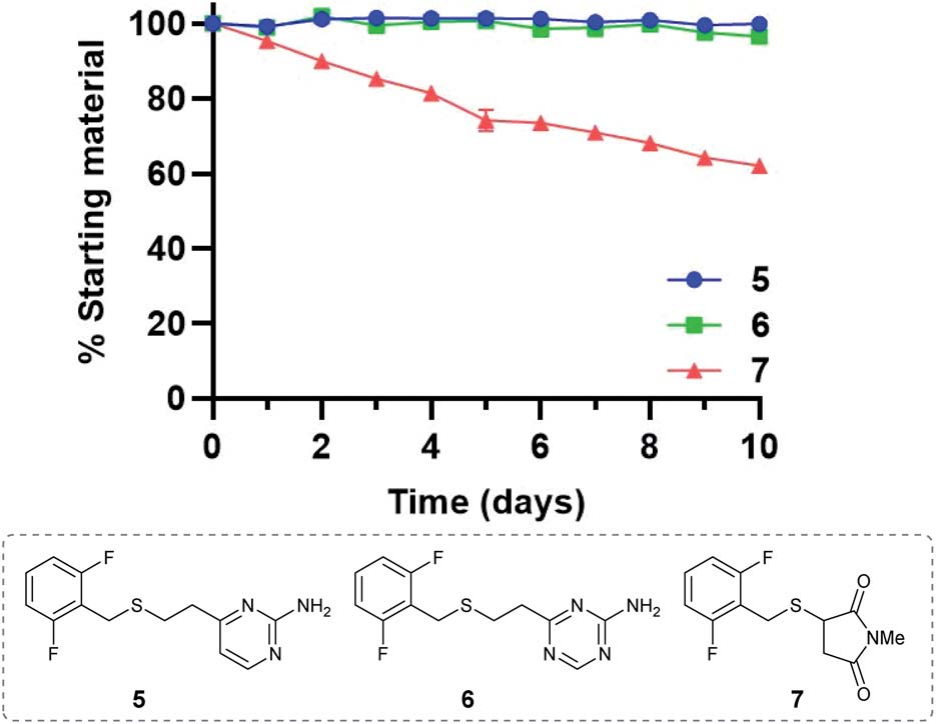
Stability comparison of pyrimidine **5**, triazine **6**, and succinimide **7** in NaPi (pH 7.4, 50 mM) and CD_3_CN, in the presence of 1-thioglycerol (150 mM). Average of two replicates; error bars represent standard deviation of the mean.

To measure the stability of the linker–thiol conjugates under physiologically relevant conditions, thioethers **5**, **6** and **7** (15 mM) in a pH 7.4 solution were incubated with an excess of 1-thioglycerol at 37 °C (ESI Section 3†). 1-Thioglycerol was added to mimic biological thiols such as glutathione or human serum albumin and would trap any reactive species formed as a result of conjugate decomposition. Analysis by ^19^F NMR revealed that pyrimidinyl thioether **5** and triazinyl thioether **6** were exceptionally stable over the ten-day monitoring period with <5% decomposition observed (ESI Sections 3.2 and 3.3†). In contrast, the maleimide-derived conjugate **7** was unstable under these conditions, with approximately 60% starting material remaining after ten days incubation (ESI Section 3.4†). These results suggest that both vinyl-pyrimidine and -triazine scaffolds have the desired stability profile required for widespread biotherapeutic development (Fig. 2).

### Vinylheteroarenes for residue-specific protein labelling

Having quantitatively evaluated the reactivity and stability of the vinylheteroarene scaffolds in small molecule assays, attention turned to investigating if these favourable results could be translated onto cysteine-containing proteins. Human serum albumin (HSA) is the most abundant protein found in plasma, which has also been utilised as a biomacromolecular delivery system in protein–drug conjugates.^56^ Among the 35 cysteines encoded in the HSA peptide sequence, 34 are present as disulfide bonds; one cysteine (Cys34) is found unpaired. Thus, HSA was deemed to be an ideal substrate to commence protein modification investigations. Since commercially available HSA was found to be partially capped with cysteine through a disulfide bond, they were first treated with dithiothreitol (DTT) to reveal the uncapped protein (ESI Section 4.1†), which was subsequently used for bioconjugation studies.

To determine the reactivity of vinylpyrimidine **2** with HSA, extensive screening of reaction conditions was carried out. Pleasingly, >95% conversion to **HSA-2** was observed by LCMS analysis upon treatment of HSA with 20 equivalents of **2** at 37 °C for 2 h in a trisaminomethane buffer (Tris·HCl, pH 8, 50 mM) containing 5% DMSO (v/v; see Table S5† for optimisation). Given these encouraging results, we were eager to evaluate the generality of this methodology by modifying HSA with a range of functional moieties. Using optimised bioconjugation conditions, HSA was reacted with vinylpyrimidine reagents bearing an alkyne biorthogonal handle **8**, a nitrobenzoxadiazole (NBD) fluorophore **9**, and a biotin tag **10**, yielding the corresponding bioconjugates **HSA-8**, **HSA-9**, **HSA-10**, respectively, all in >95% conversion by LCMS analysis (Fig. S37–S39†).

**Fig. 3:**
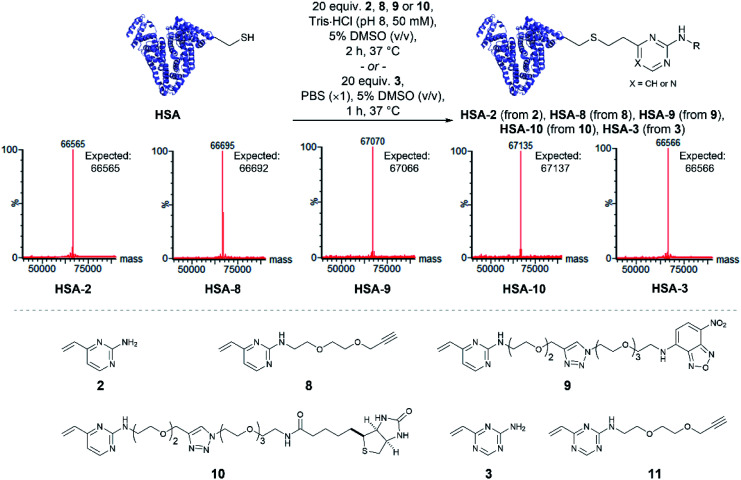
Modification of human serum albumin (HSA) using vinylpyrimidine and vinyltriazine linkers. The modification of HSA with a variety of functional units including alkyne reactive handle, fluorophore and biotin was successfully achieved.

The reactivity of vinyltriazine **3** with HSA was also investigated. After extensive screening of reaction conditions (see Table S6† for optimisation), it was found that treatment of HSA with vinyltriazine **3** (20 molar equivalents) for 1 h at 37 °C, in phosphate-buffered saline (PBS, ×1, pH 7.3) containing 5% DMSO (v/v) produced the desired bioconjugate **HSA-3** with >95% conversion by LCMS analysis (Fig. S40†). However, subsequent attempts to modify HSA with alkyne-bearing vinyltriazine **11** generated a mixture of unmodified, singly modified and doubly modified protein, perhaps due to reaction with another nucleophilic site, *e.g.* the N-terminus. These data suggest that the vinylpyrimidine scaffolds have the best combination of reactivity, selectivity and stability for general widespread use in protein modification (Fig. 3).

### Modification of cysteine-engineered antibodies and biological evaluation of antibody–drug conjugates

Encouraged by the positive properties observed for vinylpyrimidine reagents, we turned our attention towards generating a homogeneous and functional ADC using these linkers. One approach for generating ADCs uses antibodies engineered with additional surface-exposed cysteines, which allows conjugation of cytotoxic payloads to produce highly homogeneous ADCs with a defined modification site and drug-to-antibody ratio.^57^ Antibodies targeting the extracellular domain of the HER2 receptor tyrosine kinase have demonstrated immense clinical benefit for many years, particularly for the treatment of HER2-positive breast cancer.^58^ Several ADCs targeting HER2 have also undergone development with Kadcyla®^59^ and Enhertu®^60^ obtaining FDA approval. The utility of our linkers was evaluated using an anti-HER2 IgG1 antibody **mAb1** which has a cysteine insertion after position 239 in each heavy chain.^61^ The biological activity of ADCs generated using this antibody have been demonstrated previously *in vitro* and in *in vivo* mouse xenografts.^61^

**Fig. 4:**
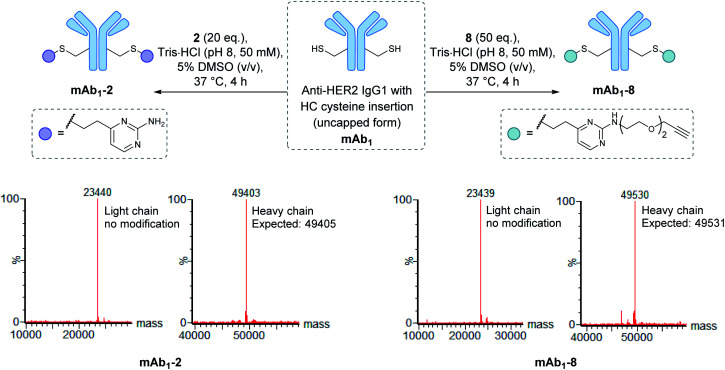
The modification of **mAb1** with linker **2** and **8**. The samples were deglycosylated and reduced prior to LCMS analysis.

To modify **mAb1** with a variety of payloads, this antibody was first subjected to a reduction-partial reoxidation protocol to unmask the free thiols of the inserted cysteine residues,^61^ whilst maintaining the intra- and inter-chain disulfides (ESI Section 5.1†). Next, the partially reduced antibody was treated with 20 equivalents of vinylpyrimidine **2** (*i.e.* 10 equivalents per cysteine) in Tris·HCl buffer (pH 8, 50 mM) for 4 hours at 37 °C. Analysis of the reaction *via* LCMS revealed >95% conversion to the desired conjugate **mAb1-2** (Fig. S43†). Pleasingly, modification was only observed on the heavy chains, where the engineered cysteines are located. Crucially, no modification was observed on the light chains, highlighting the excellent chemoselectivity of vinylpyrimidines for cysteines. Using analogous reaction conditions, the alkyne-modified bioconjugate **mAb1-8** was generated with >95% conversion by reaction of partially reduced **mAb1** with vinylpyrimidine **8** (Fig. 4 and S44†).

With a reactive alkyne handle installed on **mAb1**, its ability to be modified with functional payloads was investigated (ESI Sections 5.4 to 5.6†). First, azido-biotin **12** was reacted in the presence of CuSO_4_·5H_2_O, tris(3-hydroxypropyltriazolylmethyl)amine (THPTA) and sodium ascorbate, with LCMS analysis revealing >95% conversion to the desired conjugate **mAb1-8-12** (Fig. S45†). Similarly, **mAb1** was reacted with Alexa Fluor 488 azide **13**, yielding a fluorophore-to-antibody ratio of 1.9 *via* UV-vis analysis, indicating a near-complete conversion to the desired **mAb1-8-13**. Furthermore, sodium dodecyl sulfate-polyacrylamide gel electrophoresis (SDS-PAGE) analysis of **mAb1-8-13** displayed fluorescence for the heavy chains only, demonstrating modifications to be located on the heavy chains, consistent with mass spectrometry analysis (ESI Section 5.7†). The successful synthesis of **mAb1-8-12** and **mAb1-8-13** demonstrate that the alkyne-bearing vinylpyrimidine linker is compatible with on-protein copper-catalysed azide–alkyne cycloaddition (CuAAC) reactions.

**Fig. 5:**
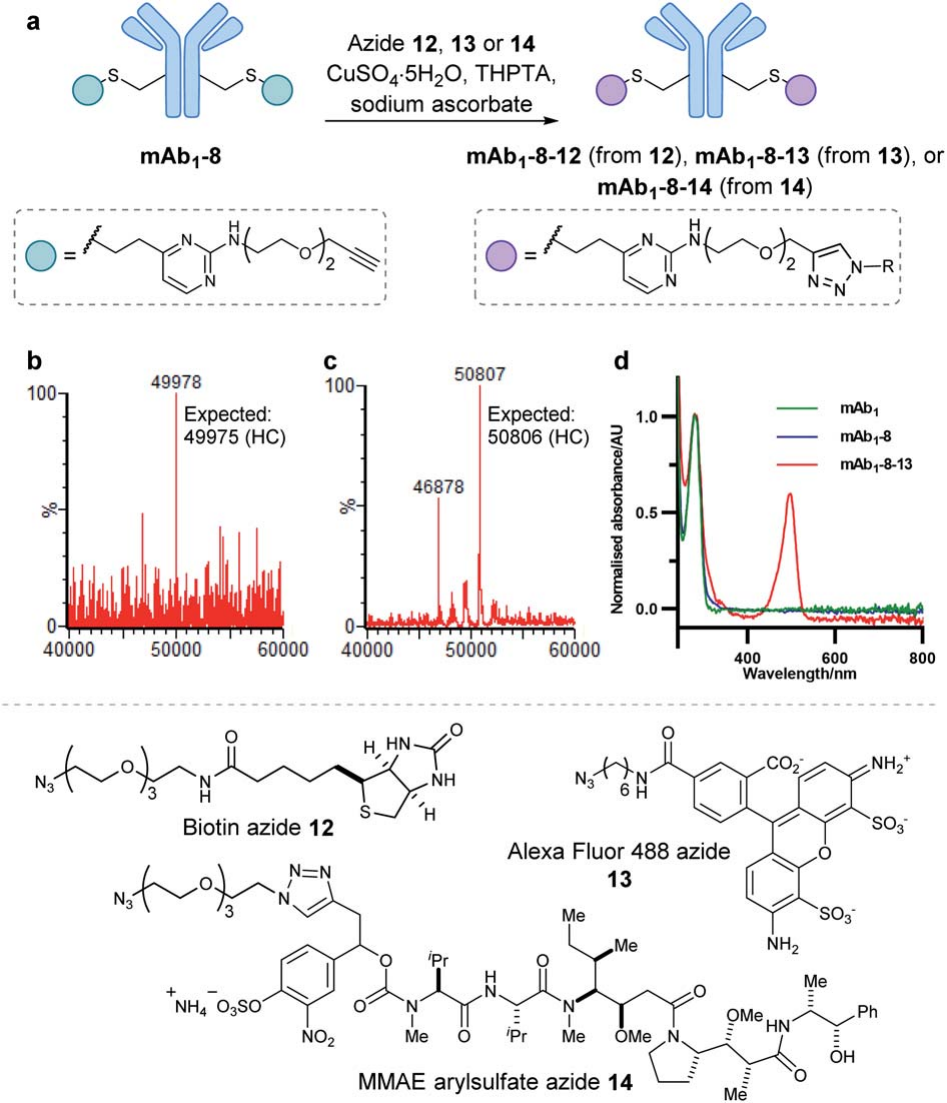
(a) On-protein copper catalysed azide–alkyne cycloaddition (CuAAC) reactions to functionalise alkynyl antibody **mAb1-8**. This antibody was successfully modified with biotin **12**, Alexa Fluor 488 fluorophore **13** and arylsulfate-MMAE **14**. (b) Deconvoluted mass spectrum of **mAb1-8-12** heavy chain. (c) Deconvoluted mass spectrum of **mAb1-8-14** heavy chain. Species with 46 878 Da correspond to light chain dimer. (d) UV-vis spectrum of unmodified **mAb1**, **mAb1-8**, and **mAb1-8-13**. Bioconjugate **mAb1-8-13** displayed a fluorophore-to-antibody ratio of 1.9.

With these promising results in hand, we proceeded to synthesise an ADC using this linker system. Thus, an appropriate azide-modified cytotoxic warhead was required. Monomethyl auristatin E (MMAE) is an anti-mitotic drug with sub-nanomolar potency, commonly used in ADC research. It was thought that incorporation of a cleavable moiety to enable release of unmodified MMAE from the antibody would achieve optimal cytotoxicity.^62^ We recently reported the use of sulfatase-cleavable linkers for use in MMAE-containing ADCs.^42^ Accordingly, MMAE-arylsulfate-azide **14** was synthesised and subsequently reacted with alkynyl antibody **mAb1-8** in the presence of CuSO_4_·5H_2_O, THPTA and sodium ascorbate, with LCMS analysis revealing >95% conversion to the desired ADC **mAb1-8-14** (Fig. 5 and S46†).

**Fig. 6:**
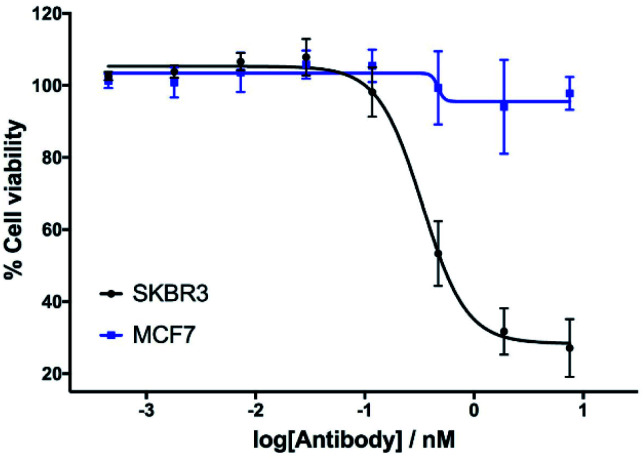
The *in vitro* evaluation of ADC **mAb1-8-14** in HER2-positive cells (SKBR3) and HER2-negative cells (MCF7). Viability data shows the mean of three independent experiments and error bars represent standard error of the mean.

The *in vitro* cytotoxicity of the anti-HER2 ADC was then evaluated by incubation in HER2-positive SKBR3 cells and HER2-negative MCF7 cells (ESI Section 7†). Pleasingly, potent dose-dependent cytotoxicity was observed in SKBR3 cells. This demonstrates that the vinylpyrimidine linker does not negatively affect the antibody's biological processes in terms of receptor specificity, binding or internalisation. In contrast, the ADC had little effect on the viability of the HER2-negative MCF7 cells at the concentrations tested. These *in vitro* studies highlight the potential of the vinylpyrimidine platform to generate clinically relevant biotherapeutics (Fig. 6).

### Labelling of cysteine-containing proteins in cell lysate

**Fig. 7:**
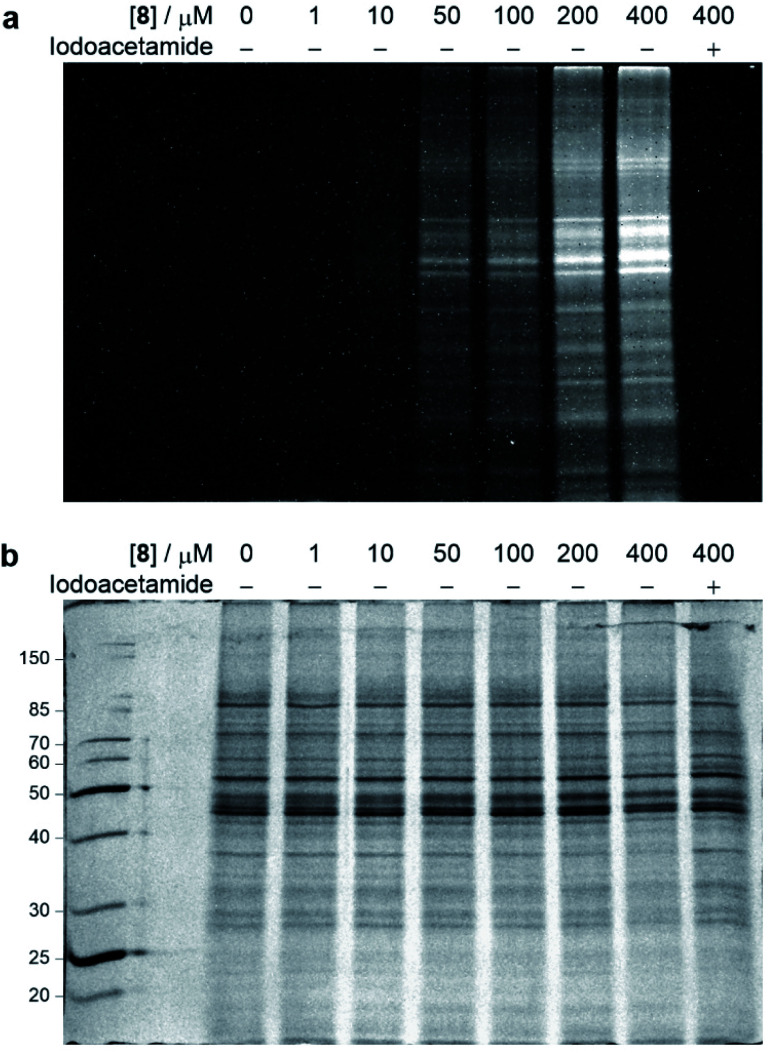
SDS-PAGE analysis of MCF7 cell lysate labelling studies. Cell lysates were first labelled with varying concentrations of probe **8**, and further modified with Alexa Fluor 488 azide **13***via* a CuAAC reaction. For cysteine blocking studies, cell lysates were first pre-incubated with iodoacetamide. (a) In-gel fluorescence of cell lysates. (b) Coomassie staining of cell lysates. All lanes were prepared under reducing conditions. Molecular weight ladder in kDa. Refer to ESI Section 8† for further details.

With the chemoselective modification of cysteine residues validated in isolated protein and antibody systems, we wanted to explore the linker's potential to probe the presence of proteome-wide cysteine residues. Accordingly, MCF7 cell lysates (at 1 mg mL^−1^) were treated with varying concentrations of vinylpyrimidine probe **8** (0 to 400 μM) at rt for 2 h. The alkyne-tagged lysates were then labelled with Alexa Fluor 488 azide **13** in the presence of CuSO_4_·5H_2_O, THPTA and sodium ascorbate (ESI Section 8.2†). The Alexa Fluor 488-labelled proteins were separated *via* SDS-PAGE followed by visualisation by in-gel fluorescence scanning, which revealed concentration-dependent labelling of lysates (Fig. 7). Crucially, when MCF7 cell lysates were pre-treated with iodoacetamide before incubating with probe **8**, no labelling was observed. This further supports that vinylpyrimidine **8** react with cysteine residues with exceptional chemoselectivity. These studies demonstrate that vinylpyrimidine linkers can successfully label lysates, and offers a complementary approach to methods found in literature.^63,64^

### Plasma stability

Finally, to determine the stability of bioconjugates in human plasma, the plasma stability of the vinylpyrimidine bioconjugates was determined, and compared to those synthesised using maleimide bioconjugation. It was anticipated that the synthesis of antibody–fluorophore conjugates would enable qualitative analysis of the stability profiles of the different scaffolds using SDS-PAGE. First, the interchain disulfides of the native anti-HER2 antibody trastuzumab **mAb2** were reduced with tris(2-carboxyethyl)phosphine (TCEP) hydrochloride to reveal eight reactive thiols. Then, the reduced antibody was reacted with vinylpyrimidine **8** or maleimide **15** to generate the corresponding alkynyl antibodies. Finally, the resulting alkynes were subjected to CuAAC with Alexa Fluor 488 azide **13** in the presence of CuSO_4_·5H_2_O, THPTA and sodium ascorbate, yielding antibody–fluorophore conjugates **mAb2-8-13** and **mAb2-15-13**, with a fluorophore-antibody ratio of 6.0 and 5.8, respectively (see ESI Sections 6.2 to 6.5† for full synthetic procedure). The trastuzumab–fluorophore conjugates **mAb2-8-13** and **mAb2-15-13** were then incubated in human plasma at 37 °C for eight days (ESI Section 6.6†). Analysis by SDS-PAGE revealed that maleimide conjugate **mAb2-15-13** over time formed a new fluorescence band at ∼67 kDa. This corresponds to the product formed from linker deconjugation followed by interception with serum albumin, in accordance with previous reports.^65^ In contrast, no transfer of fluorescence to any of the plasma proteins was observed for the vinylpyrimidine analogue **mAb2-8-13** over the eight-day monitoring period. These plasma stability data confirm the exquisite stability of vinylpyrimidine conjugates. Further stability studies were conducted in human serum, which also demonstrated the superior stability of vinylpyrimidine conjugates over maleimide conjugates (Fig. S52† and 8).

**Fig. 8:**
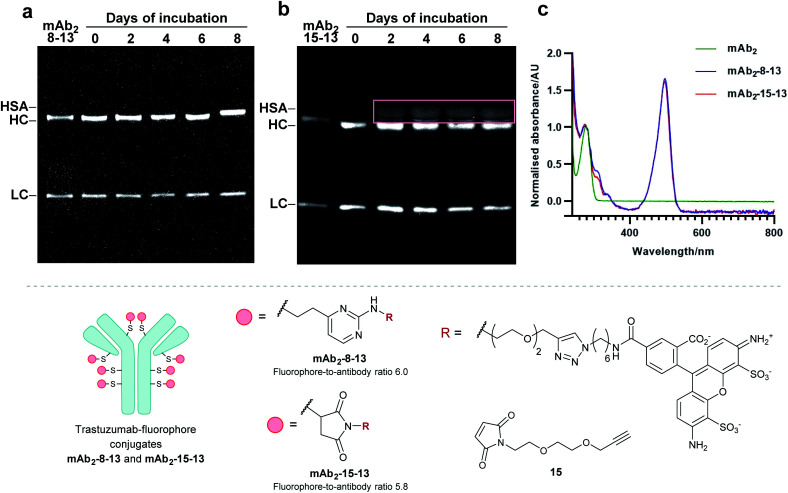
Determination of plasma stability by SDS-PAGE for (a) **mAb2-8-13** and (b) **mAb2-15-13**, which are bioconjugates synthesised by reduction of interchain disulfides of native trastuzumab **mAb2**, followed by bioconjugation with linker **8** or **15**, respectively, and functionalisation with Alexa Fluor 488 **13**. In-gel fluorescence analysis displays no transfer of fluorescence for vinylpyrimidine-derived conjugate **mAb2-8-13**. In contrast, fluorescence transfer to serum proteins (indicated by the beige box) was observed for maleimide-derived **mAb2-15-13**. All lanes were prepared under reducing conditions. (c) UV-vis spectra for **mAb2**, **mAb2-8-13**, and **mAb2-15-13**.

## Conclusion

In this work, a series of vinylheteroarene scaffolds were explored for cysteine modification. Through several small molecule reactivity, chemoselectivity and stability assays, vinylpyrimidine reagents emerged as the most promising candidates for selective modification of proteinogenic cysteine residues. Vinylpyrimidine reagents demonstrated rapid reactivity and chemoselectivity towards cysteines, and the resulting conjugates were exceptionally stable under physiologically relevant conditions. These reagents were then elaborated to modify cysteine residues in protein substrates, culminating in the synthesis of a homogeneous ADC. Biological assays revealed the modified proteins retain their biological function, demonstrated by cell-specific cytotoxicity. Utilising the linker's cysteine selective reactivity, the linkers were used as a probe for proteinogenic cysteine residues in cell lysates. Finally, the bioconjugates exhibited superior stability in human plasma compared to the widely used maleimide reagents. The advantageous properties and synthetic accessibility of the vinylheteroarene scaffold lends itself toward widespread usage, both in the development of biological probes and next generation biotherapeutics.

## Author contributions

Research was conceived by all authors. Experiments were designed and performed by HS and SJW. Cell lysate preparation and in vitro cytotoxicity studies were conducted by SJW and JSC. Compound 14 was prepared by JDB. Antibodies used in this study were provided by JSP. The manuscript was written and proofread by all authors.

## Conflicts of interest

There are no conflicts to declare.

## Supplementary Material

SC-012-D1SC02722K-s001
