# Supplementary material for: Rapid and robust cysteine bioconjugation with vinylheteroarenes
Source: Chem Sci. 2021 Jun 7;12(26):9060–8. doi: 10.1039/d1sc02722k (PMC8261766; doi:10.1039/d1sc02722k)

Electronic Supplementary Material (ESI) for Chemical Science.
This journal is © The Royal Society of Chemistry 2021

Rapid and robust cysteine bioconjugation with vinylheteroarenes

Hikaru Seki,[a] Stephen J. Walsh,[a,b] Jonathan D. Bargh,[a] Jeremy S. Parker,[c] Jason S. Carroll,[b] and David R. Spring[a]*

a. *Department of Chemistry, University of Cambridge, Lensfield Road, Cambridge, CB2 1EW, UK. Email: spring@ch.cam.ac.uk;*
b. *Cancer Research UK Cambridge Institute, University of Cambridge, Robinson Way, Cambridge, CB2 0RE, UK.*
c. *Early Chemical Development, Pharmaceutical Sciences, R&D, AstraZeneca, Macclesfield, UK*

1. General experimental 4
2. Rate determination of conjugate addition 7
2.1. General procedure 7
2.2. Cysteine reactivity with vinylheteroarenes in CD_3OD/NaPi (pH 8, 50 mM) 9
2.3. Lysine reactivity with vinylheteroarenes in CD_3OD/NaPi (pH 8, 50 mM) 14
2.4. *N*-Terminus reactivity with vinylheteroarenes in CD_3OD/NaPi (pH 8, 50 mM) 19
2.5. Effect of buffer pH on vinylpyrimidine-cysteine reactivity 24
2.6. Effect of buffer concentration on vinylpyrimidine-cysteine reactivity 29
3. Thioether stability studies 32
3.1. General procedure 32
3.2. Stability data for pyrimidine 5 33
3.3. Stability data for triazine 6 34
3.4. Stability data for succinimide 7 35
4. Modification of human serum albumin 36
4.1. Reduction of human serum albumin 36
4.2. Optimisation for the reaction between HSA and vinylpyrimidine 2 38
4.3. Synthesis of HSA-2 using optimised conditions 39
4.4. Synthesis of HSA-8 40
4.5. Synthesis of HSA-9 41
4.6. Synthesis of HSA-10 42
4.7. Optimisation for the reaction between HSA and vinyltriazine 3 43
4.8. Synthesis of HSA-3 under optimised conditions 44
4.9. SDS-PAGE analysis of HSA conjugates 45
5. Modification of cysteine-engineered antibody 46
5.1. Reduction of cysteine-engineered antibody mAb_1 46
5.2. Synthesis of mAb_1-2 48

5.3. Synthesis of mAb_1-8 49
5.4. Synthesis of mAb_1-8-12 50
5.5. Synthesis of mAb_1-8-13 51
5.6. Synthesis of mAb_1-8-14 51
5.7. SDS-PAGE analysis of mAb_1 conjugates 53
6. Preparation of fluorophore-modified trastuzumab and plasma stability 54
6.1. Mass spectra of native trastuzumab 54
6.2. Synthesis of mAb_2-8 55
6.3. Synthesis of mAb_2-8-13 57
6.4. Synthesis of mAb_2-15 57
6.5. Synthesis of mAb_2-15-13 59
6.6. Plasma stability studies 60
7. *In vitro* cytotoxicity studies 62
8. Cell lysate labelling 63
8.1. Preparation of cell lysate 63
8.2. Labelling of cell lysate 63
9. Chemical synthesis 65
10. Spectra of small molecules 76
11. HPLC Traces 96

1. General experimental

All solvents and reagents were used as received unless otherwise stated. Ethyl acetate, methanol, dichloromethane, acetonitrile and toluene were distilled from calcium hydride. Diethyl ether was distilled from a mixture of lithium aluminium hydride and calcium hydride. Petroleum ether refers to the fraction between 40–60 °C upon distillation. Tetrahydrofuran was dried using Na wire and distilled from a mixture of lithium aluminium hydride and calcium hydride with triphenylmethane as indicator.

Non-aqueous reactions were conducted under a stream of dry nitrogen using oven dried glassware. Temperatures of 0 °C were maintained using an ice-water bath. Room temperature (rt) refers to ambient temperature.

Yields refer to spectroscopically and chromatographically pure compounds unless otherwise stated. Reactions were monitored by thin layer chromatography (TLC) or liquid chromatography mass spectroscopy (LC-MS). TLC was performed using glass plates pre-coated with Merck silica gel 60 F254 and visualized by quenching of UV fluorescence (λ_{max} = 254 nm) or by staining with potassium permanganate or *para*-anisaldehyde. Retention factors (R_f) are quoted to 0.01.

LC-MS was carried out using a Waters ACQUITY HClass UPLC with an ESCi Multi-Mode Ionisation Waters SQ Detector 2 spectrometer using MassLynx 4.1 software; EI refers to the electrospray ionisation technique; LC system: solvent A: 2 mM NH_4OAc in H_2O/MeCN (95:5); solvent B: MeCN; solvent C: 2% formic acid; column: ACQUITY UPLC® CSH C18 (2.1 mm × 50 mm, 1.7 μm, 130 Å) at 40 °C; gradient: 5 – 95 % B with constant 5 % C over 1 min at flow rate of 0.6 mL/min; detector: PDA eλ Detector 220 – 800 nm, interval 1.2 nm.

Flash column chromatography was carried out using slurry-packed Merck 9385 Kieselgel 60 SiO_2 (230-400 mesh) under a positive pressure.

Reverse-phase flash column chromatography was carried out using a Combiflash Rf200 automated chromatography system with Redisep® reverse-phase C18-silica flash columns (20-40 μm).

Analytical high performance liquid chromatography (HPLC) was performed on Agilent 1260 Infinity machine, using a Supelcosil™ ABZ+PLUS column (150 mm × 4.6 mm, 3 μm) with a linear gradient system (solvent A: 0.05% (v/v) TFA in H_2O; solvent B: 0.05% (v/v) TFA in MeCN) over 20 min at a flow rate of 1 mL/min, and UV detection (λ_{max} = 220 – 254 nm).

Infrared (IR) spectra were recorded neat on a Perkin-Elmer Spectrum One spectrometer with internal referencing. Selected absorption maxima (ν_{max}) are reported in wavenumbers (cm^{-1}).

Proton and carbon nuclear magnetic resonance (NMR) were recorded using an internal deuterium lock on Bruker DPX-400 (400 MHz, 101 MHz), Bruker Avance 400 QNP (400 MHz, 101 MHz) and Bruker Avance 500 Cryo Ultrashield (500 MHz, 126 MHz). In proton NMR, chemical shifts (δ_H) are reported in parts per million (ppm), to the nearest 0.01 ppm and are referenced to the residual non-deuterated solvent peak ($CHCl_3$: 7.26, CHD_2OD: 3.31, HOD: 4.79). Coupling constants (*J*) are reported in Hertz (Hz) to the nearest 0.1 Hz. Data are reported as follows: chemical shift, integration, multiplicity (s = singlet; d = doublet; t = triplet; q = quartet; qn = quintet; sep = septet; m = multiplet; app = apparent; br = broad; or as a combination of these, e.g. dd, dt etc.), and coupling constant(s). In carbon NMR, chemical shifts (δ_C) are quoted in ppm, to the nearest 0.1 ppm, and are referenced to the residual non-deuterated solvent peak ($CDCl_3$: 77.16, CD_3OD: 49.00).

High resolution mass spectrometry (HRMS) measurements were recorded with a Micromass Q-TOF mass spectrometer or a Waters LCT Premier Time of Flight mass spectrometer. Mass values are reported within the error limits of ±5 ppm mass units. ESI refers to the electrospray ionisation technique.

Protein LCMS was performed on a Xevo G2-S TOF mass spectrometer coupled to an Acquity UPLC system using an Acquity UPLC BEH300 C4 column (1.7 µm, 2.1 × 50 mm). H_2O with 0.1% formic acid (solvent A) and 95% MeCN and 5% water with 0.1% formic acid (solvent B), were used as the mobile phase at a flow rate of 0.2 mL/min. The gradient was programmed as follows: 95% A for 0.93 min, then a gradient to 100% B over 4.28 min, then 100% B for 1.04 minutes, then a gradient to 95% A over 1.04 min. The electrospray source was operated with a capillary voltage of 2.0 kV and a cone voltage of 40 or 150 V. Nitrogen was used as the desolvation gas at a total flow of 850 L/h. Total mass spectra were reconstructed from the ion series using the MaxEnt algorithm preinstalled on MassLynx software (v4.1 from Waters) according to the manufacturer's instructions.

Non-reducing Tris-Glycine SDS-PAGE with 12% acrylamide with 4% stacking gel was performed as standard. Broad range molecular weight marker (10-200 kDa, New England BioLabs) was run in all gels. Samples were prepared by mixing with loading dye and heated to 90 °C for 5 minutes. Loading dye containing β-mercaptoethanol was used to prepare samples under reducing conditions. Gels were run at constant voltage (160 V) for 70 min to 90 min in

×1 Laemmli running buffer. All gels were stained with Coomassie brilliant blue dye and imaged on a Syngene gel imaging system.

Monoclonal antibodies were deglycosylated and reduced prior to LCMS analysis. This was typically performed by adding 0.1 µL of peptide:*N*-glycosidase F (PNGase F; New England BioLabs Catalogue number P0704S) to a solution of antibody (10 µL at 1 µM) and was left to stand at rt for 15 h. To this solution, TCEP·HCl (1 µL, 5 mM in H_2O) was added and was left to stand at rt for 10 minutes before analysis.

UV-visible (UV-vis) spectrums were obtained using a NanoDrop™ One spectrophotometer (ThermoFisher). Raw data was plotted using GraphPad Prism software (version 8). The following equation[1,2] was used to calculate fluorophore-to-antibody ratio for AlexaFluor488-containing antibodies, where ε_{280} = 215380 M^{-1} cm^{-1} is the molar extinction coefficient trastuzumab at 280 nm; ε_{495} = 71000 M^{-1} cm^{-1} is the molar extinction coefficient for AlexaFluor488 at 495 nm; Abs_{495} and Abs_{280} are absorbance at 495 nm and 280 nm, respectively. A correction factor of 0.11 was used to account for AlexaFluor488 absorbance at 280 nm.

$$\text{Fluorophore to antibody ratio } = \frac{\text{Abs}_{495}/\varepsilon_{495}}{[\text{Abs}_{280} - 0.11\text{Abs}_{495}]/\varepsilon_{280}}$$

[1] Maruani, A.; Savoie, H.; Bryden, F.; Caddick, S.; Boyle, R.; Chudasama, V. *Chem. Commun.* **2015**, *51*, 15304.
[2] Walsh, S. J.; Omarjee, S.; Galloway, W. R. J. D.; Kwan, T. T.-L.; Sore, H. F.; Parker, J. S.; Hyvönen, M.; Carroll, J. S.; Spring, D. R. *Chem. Sci.* **2019**, *10*, 694.

2. Rate determination of conjugate addition

2.1. General procedure

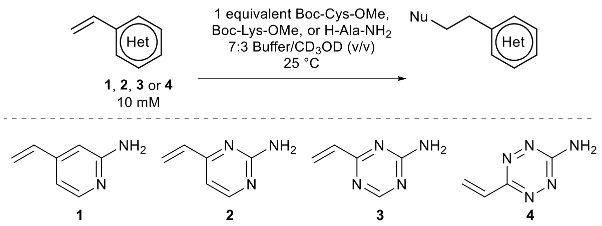

To a solution of linker **1**, **2**, **3** or **4**[3] [0.7 mL, 20 mM in 3:7 CD_3OD/buffer] in an NMR tube, a solution of Boc-Cys-OMe, Boc-Lys-OMe·HCl or H-Ala-NH_2·HCl [0.7 mL, 20 mM in 3:7 CD_3OD/buffer] was added and mixed by vigorous shaking.

After the first ^{1}H NMR spectrum (with water suppression) was acquired, subsequent measurements were taken every ~10 minutes for all nucleophile-vinylheteroarene combinations, apart from Table S1, Entries 2–3 and 11–15, where measurements were taken every ~15 seconds. The vinyl peaks at 5.52 ppm, 5.75 ppm, and 5.96 ppm were integrated to determine the concentration of substrates **1**, **2** and **3**, respectively.

The following equations were used to determine second order rate constants k_2.

$$\frac{\mathrm{d[E]}}{\mathrm{dt}} = -k_2[\mathrm{Nu}][\mathrm{E}]$$

$$\text{When } [\mathrm{Nu}] = [\mathrm{E}],\ \frac{\mathrm{d[E]}}{\mathrm{dt}} = -k_2[\mathrm{E}]^2$$

$$\text{Integration gives } \frac{1}{[\mathrm{E}]_\mathrm{t}} = k_2\mathrm{t} + \frac{1}{[\mathrm{E}]_{\mathrm{t}=0}}$$

Thus, the second order rate constant k_2 can be determined by plotting $\mathrm{y} = \frac{1}{[\mathrm{E}]_\mathrm{t}}$ and $\mathrm{x} = \mathrm{t}$; the gradient is equal to k_2.[4]

[3] For tetrazine **4**, only reactivity with Boc-Cys-OMe was examined. For results, refer to **Figure S7**.
[4] For an example of second order rate constants determined using this method, see Kamber, D. N.; Liang, Y.; Blizzard, R. J.; Liu, F.; Mehl, R. A.; Houk, K. N.; Prescher, J. A. *J. Am. Chem. Soc.* **2015**, *137*, 8388.

Table S1. Second order rate constants of vinylheteroarene reaction with Boc-Cys-OMe, Boc-Lys-OMe and H-Ala-NH_2.

Entry	Linker	Nucleophile	Buffer	Second order rate constant k_2 ($\times 10^{-3}$ $M^{-1}\cdot s^{-1}$)
1	**1**	Boc-Cys-OMe	NaPi (pH 8, 50 mM in D_2O)	4.91 ± 0.01
2	**2**			375 ± 30
3	**3**			3100 ± 30
4	**4**			n/a
5	**1**	Boc-Lys-OMe·HCl	NaPi (pH 8, 50 mM in D_2O)	0
6	**2**			0
7	**3**			0.323 ± 0.006
8	**1**	H-Ala-NH_2·HCl	NaPi (pH 8, 50 mM in D_2O)	0
9	**2**			0
10	**3**			0.944 ± 0.090
11	**2**	Boc-Cys-OMe	NaPi (pH 8, 25 mM in D_2O)	330 ± 6
12	**2**		NaPi (pH 8, 10 mM in D_2O)	213 ± 2
13	**2**	Boc-Cys-OMe	NaPi (pH 7, 50 mM in D_2O)	510 ± 1
14	**2**		NaPi (pH 6, 50 mM in D_2O)	636 ± 10
15	**2**		CD_3CO_2Na (pH 5, 50 mM in D_2O)	851 ± 7

2.2. Cysteine reactivity with vinylheteroarenes in CD_3OD/NaPi (pH 8, 50 mM)

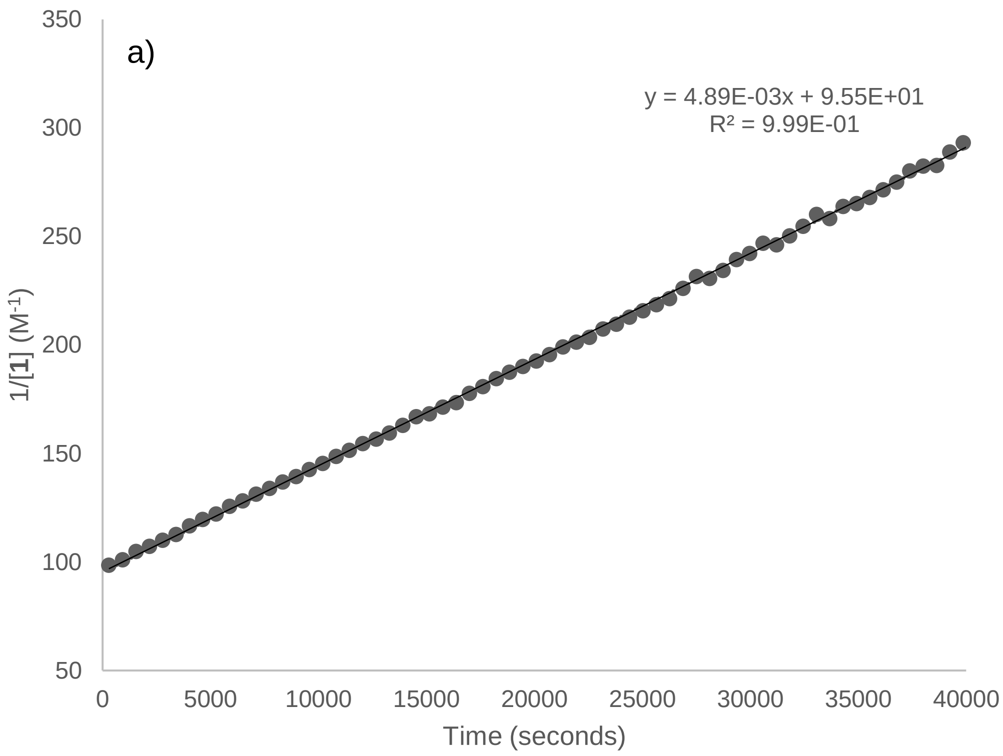

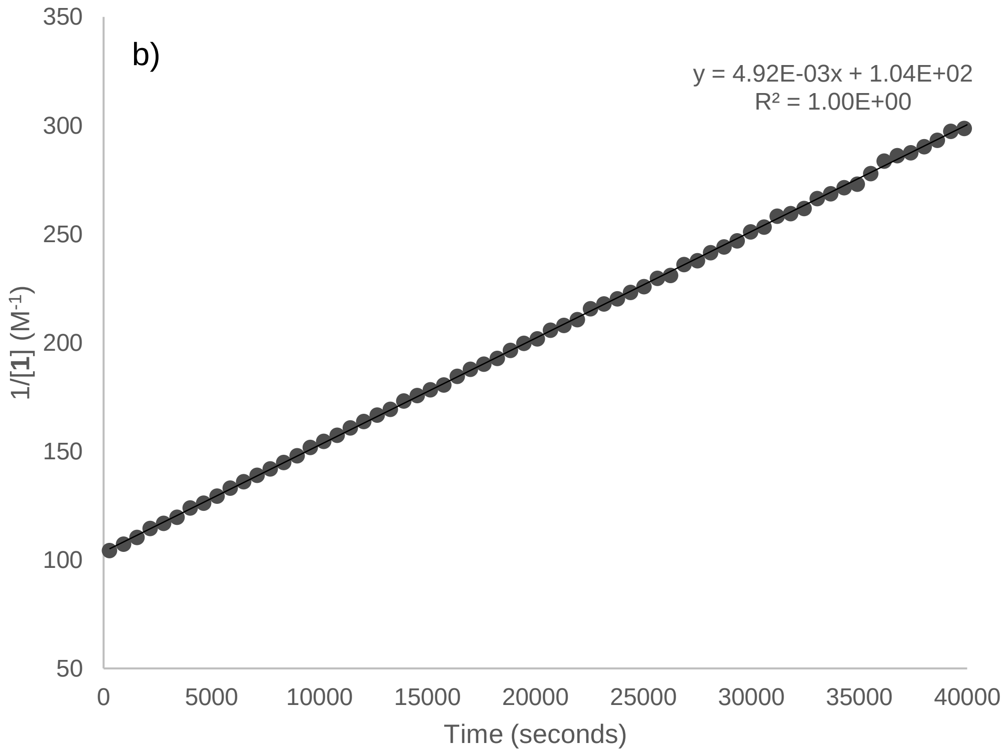

Figure S1. Kinetic data used to calculate second order rate constants for the reaction of Boc-Cys-OMe with vinylpyridine **1** in 3:7 CD_3OD/NaPi (pH 8, 50 mM in D_2O). Two measurements were taken (plots a and b) and averaged.

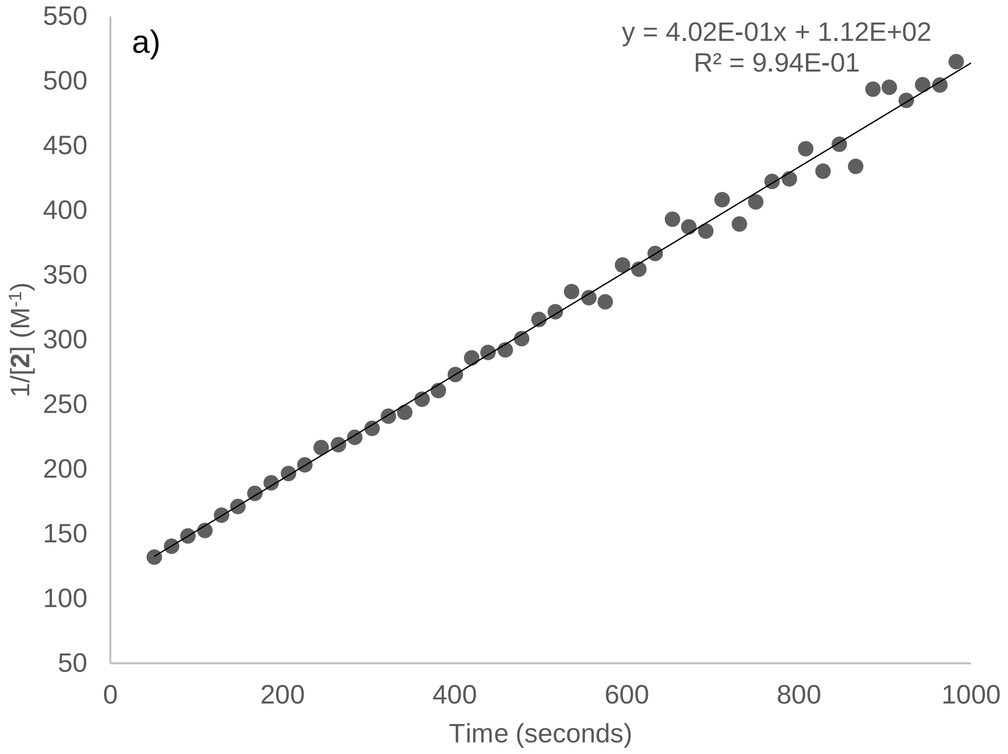

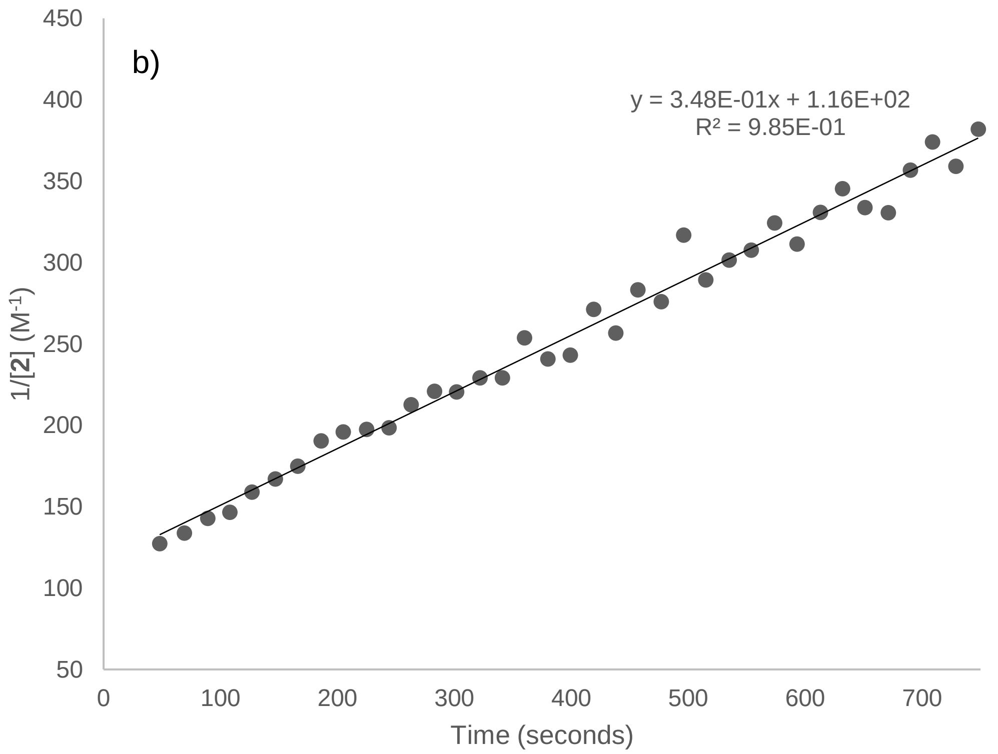

Figure S2. Kinetic data used to calculate second order rate constants for the reaction of Boc-Cys-OMe with vinylpyrimidine **2** in 3:7 CD_3OD/NaPi (pH 8, 50 mM in D_2O). Two measurements were taken (plots a and b) and averaged.

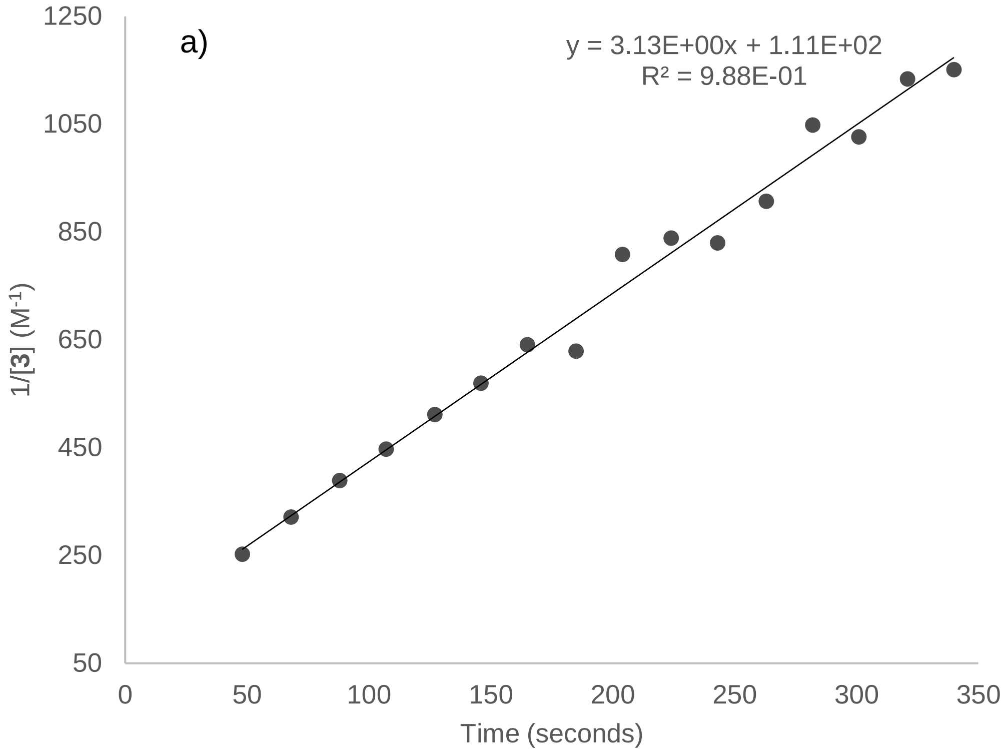

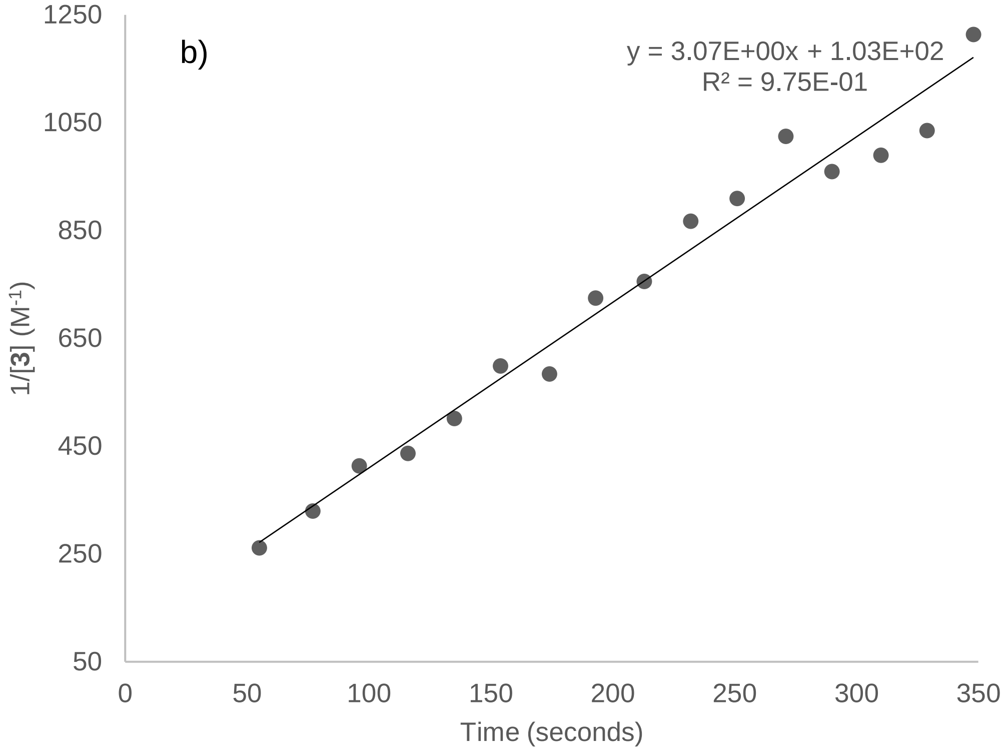

Figure S3. Kinetic data used to calculate second order rate constants for the reaction of Boc-Cys-OMe with vinyltriazine **3** in 3:7 CD_3OD/NaPi (pH 8, 50 mM in D_2O). Two measurements were taken (plots a and b) and averaged.

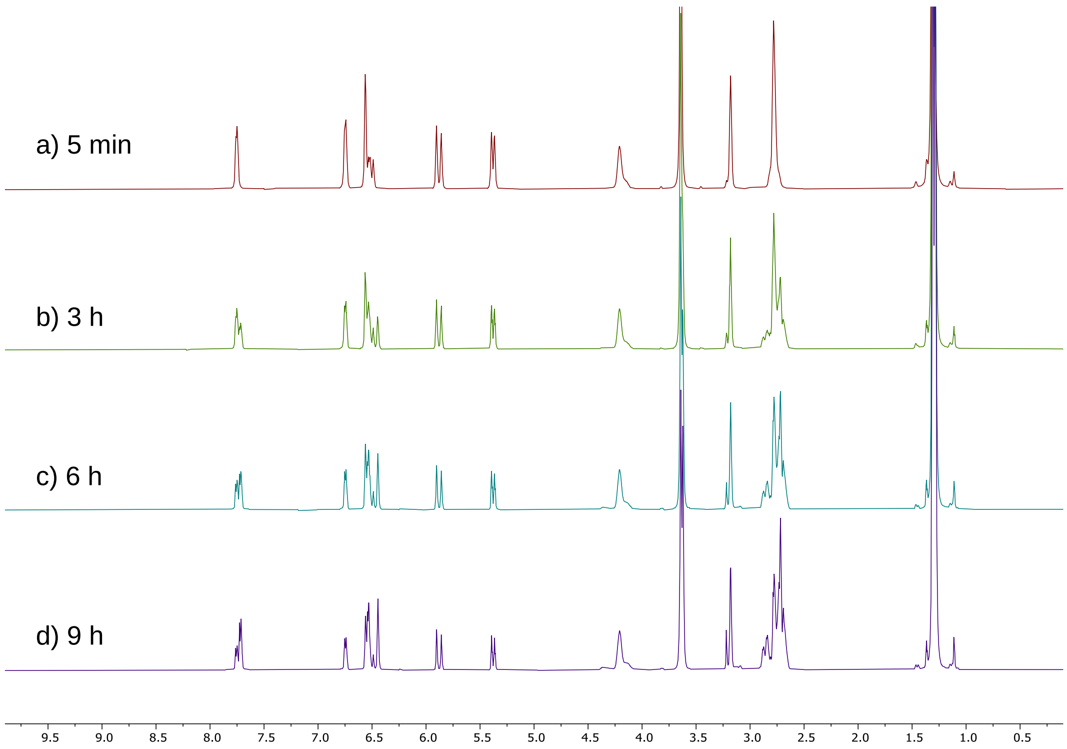

Figure S4. Representative ^{1}H NMR spectra of the reaction of vinylpyridine **1** with Boc-Cys-OMe in 3:7 CD_3OD/NaPi (pH 8, 50 mM in D_2O).

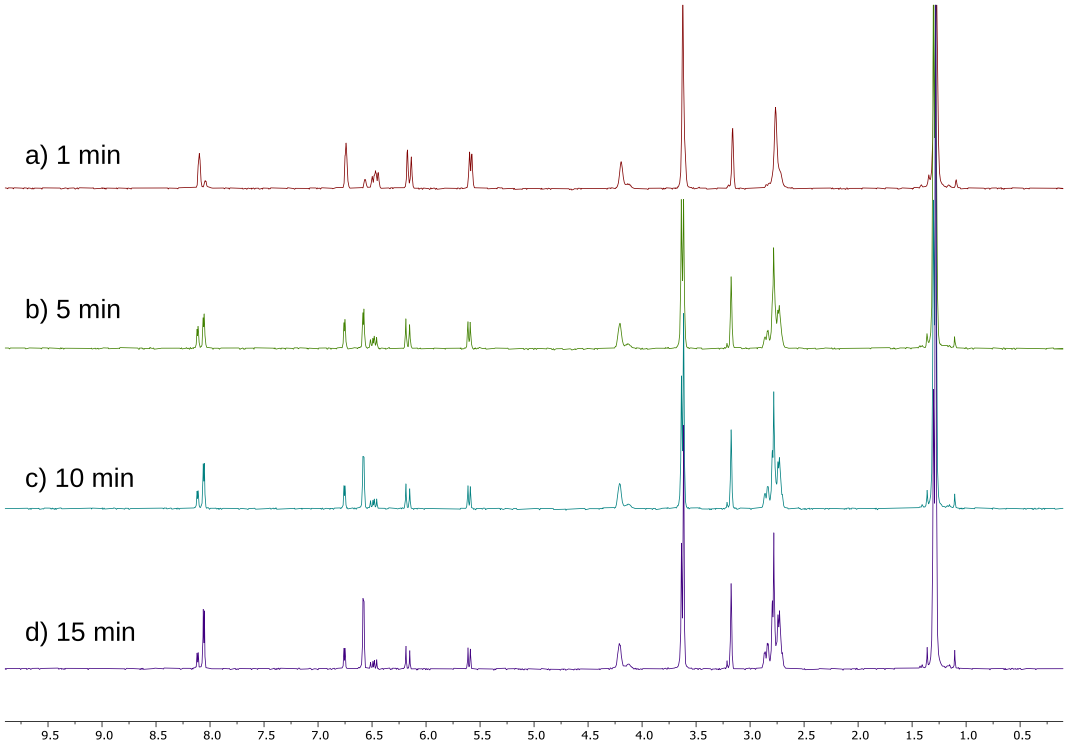

Figure S5. Representative ^{1}H NMR spectra of the reaction of vinylpyrimidine **2** with Boc-Cys-OMe in 3:7 CD_3OD/NaPi (pH 8, 50 mM in D_2O).

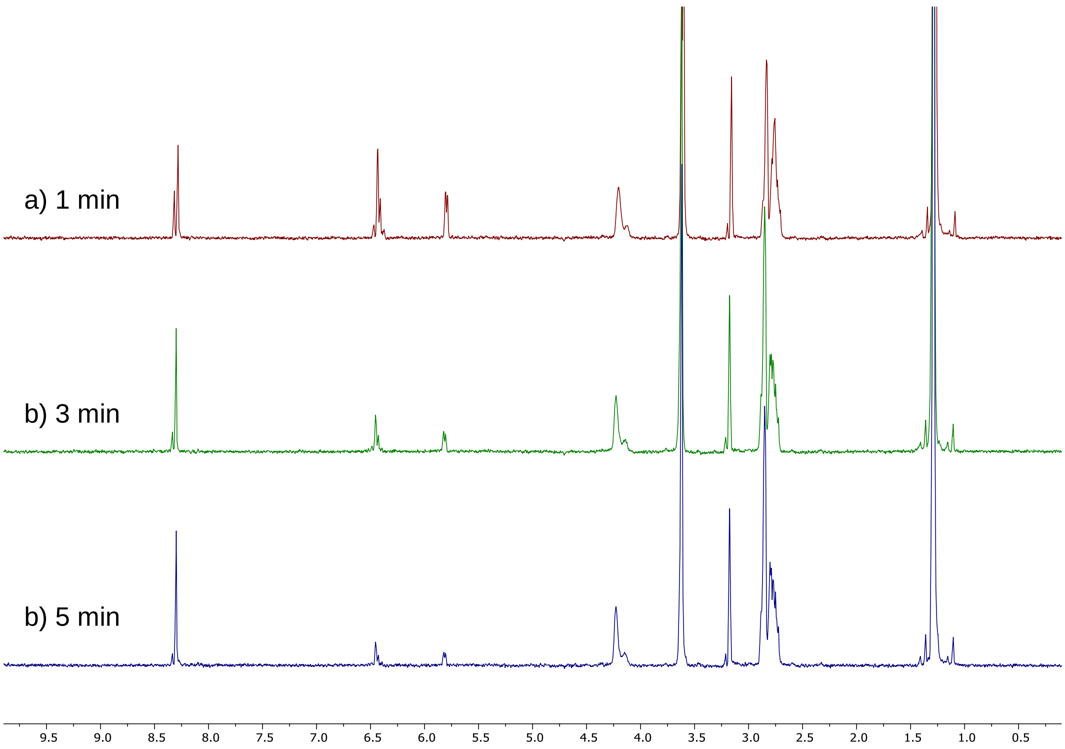

Figure S6. Representative ^{1}H NMR spectra of the reaction of vinyltriazine **3** with Boc-Cys-OMe in 3:7 CD_3OD/NaPi (pH 8, 50 mM in D_2O).

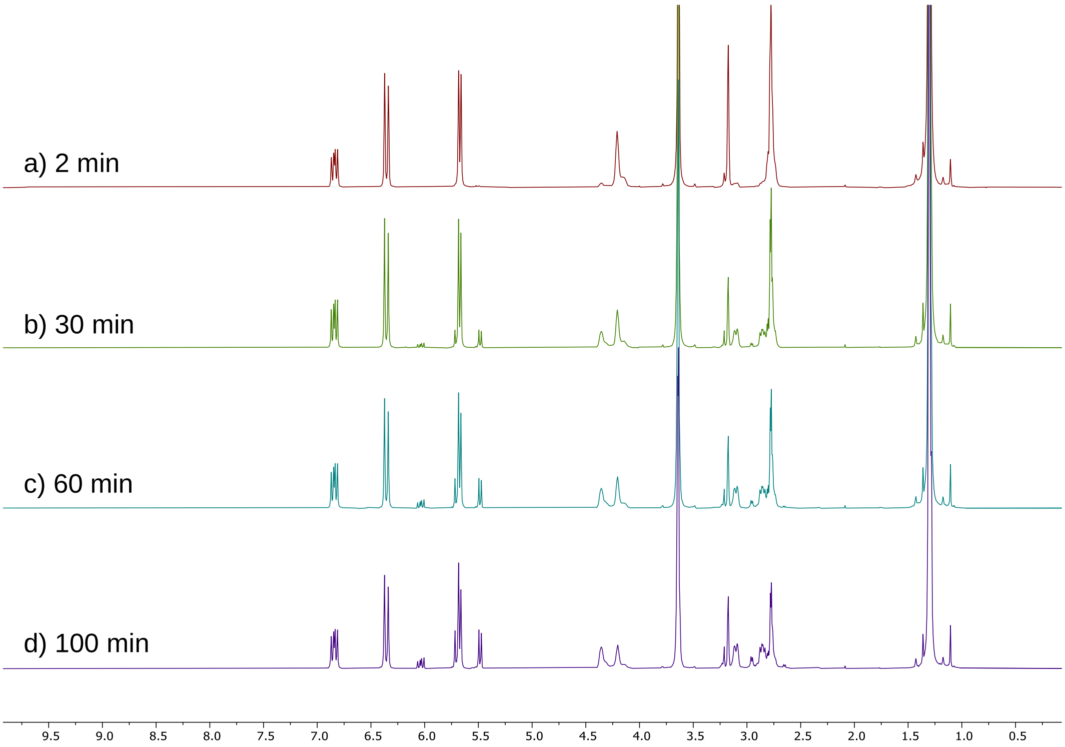

Figure S7. ^{1}H NMR spectra of the reaction of vinyltetrazine **4** with Boc-Cys-OMe in 3:7 CD_3OD/NaPi (pH 8, 50 mM in D_2O). Rather than the depletion of vinyl peaks (which indicate conjugate addition), a new set of vinyl peaks arise over time at 6.04 ppm (dd, J = 18.0, 11.3 Hz), 5.70 (overlapping) and 5.48 (dd, J = 11.3, 1.6 Hz). This suggests that Boc-Cys-OMe is reacting with the tetrazine ring rather than the vinyl group.

2.3. Lysine reactivity with vinylheteroarenes in CD_3OD/NaPi (pH 8, 50 mM)

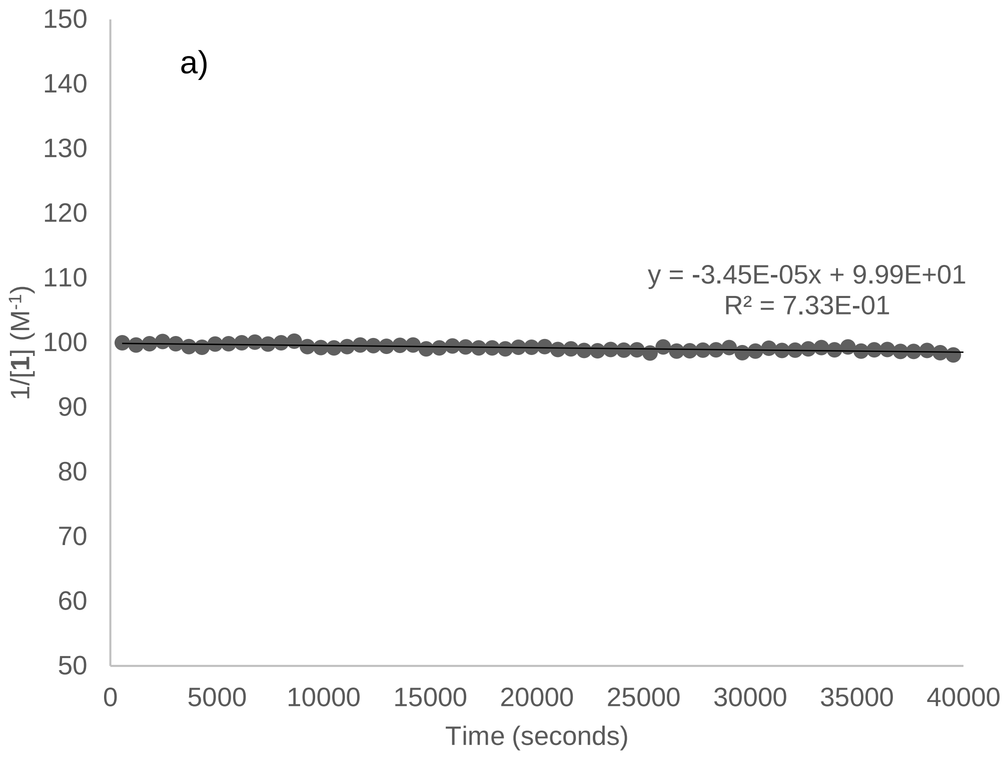

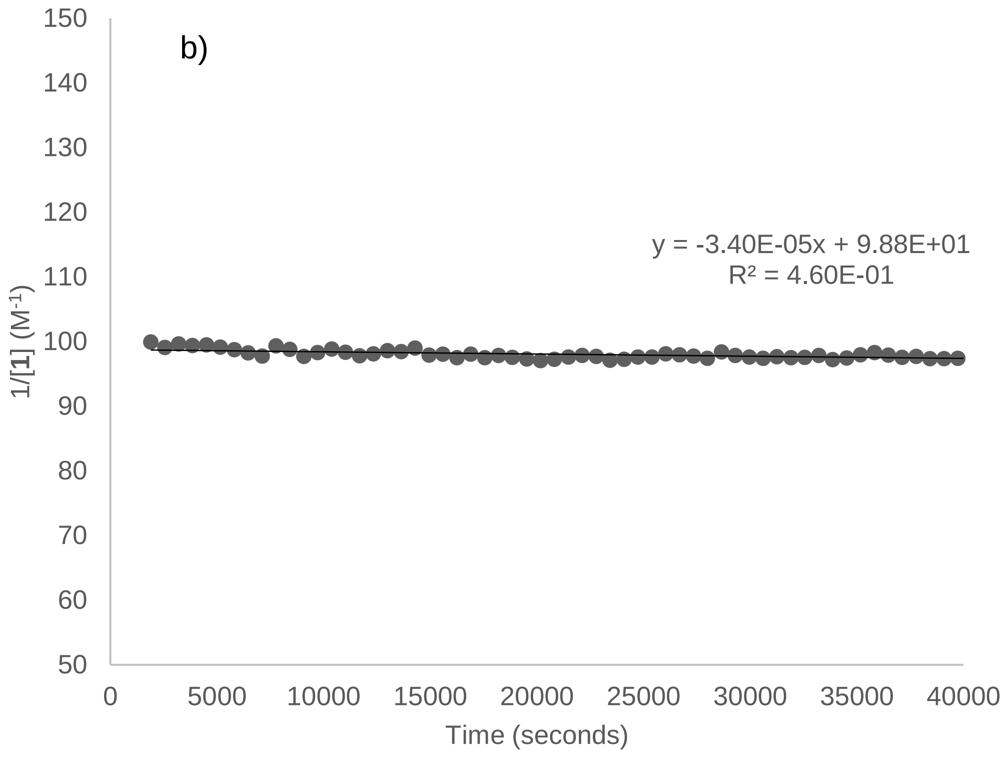

Figure S8. Kinetic data used to calculate second order rate constants for the reaction of Boc-Lys-OMe·HCl with vinylpyridine **1** in 3:7 CD_3OD/NaPi (pH 8, 50 mM in D_2O). Two measurements were taken (plots a and b) and averaged.

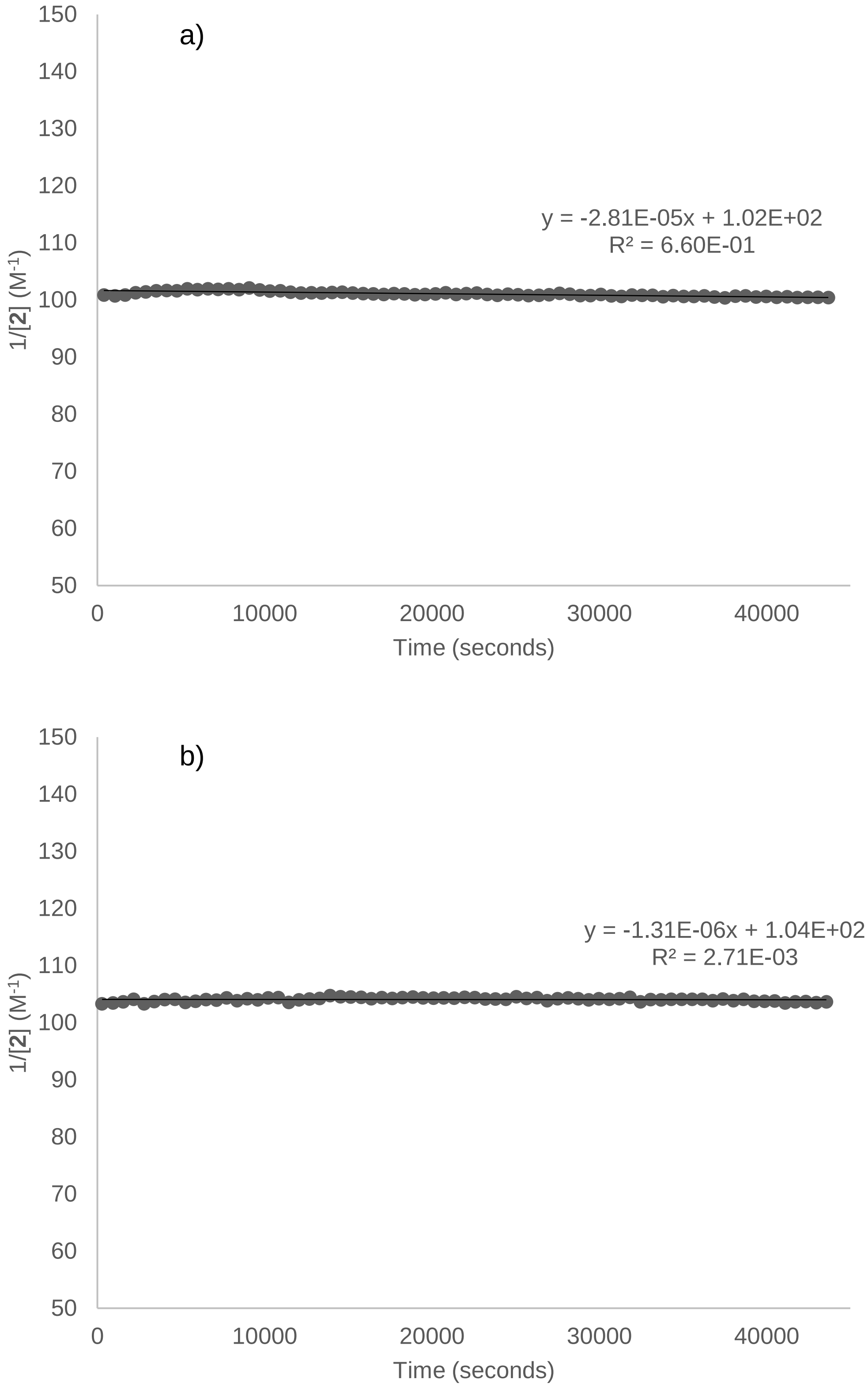

Figure S9. Kinetic data used to calculate second order rate constants for the reaction of Boc-Lys-OMe·HCl with vinylpyrimidine **2** in 3:7 CD_3OD/NaPi (pH 8, 50 mM in D_2O). Two measurements were taken (plots a and b) and averaged.

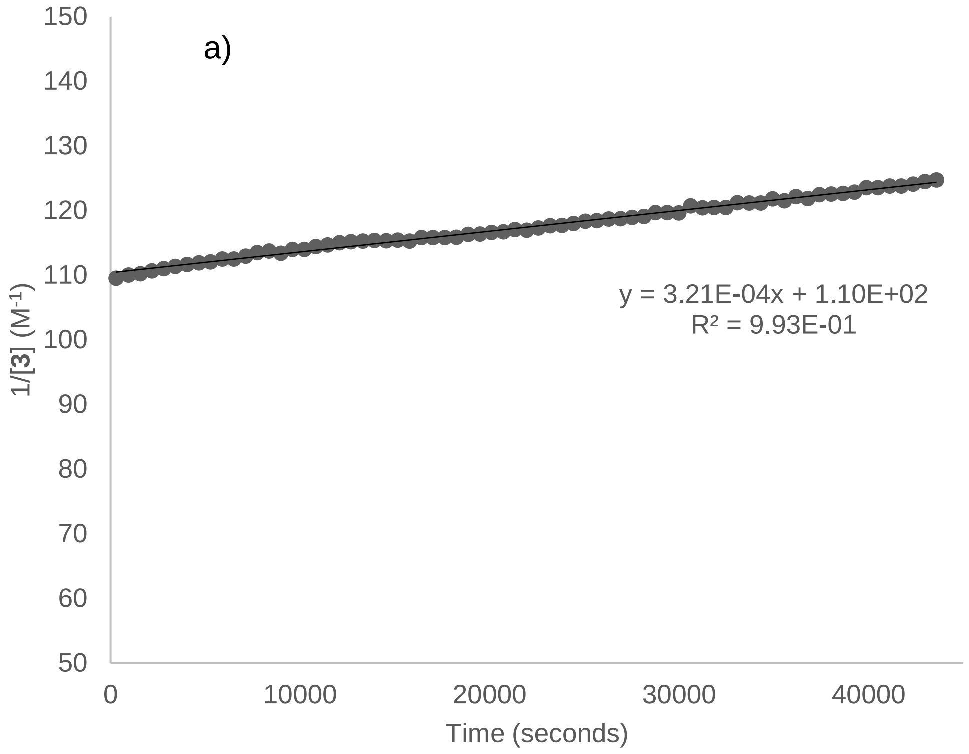

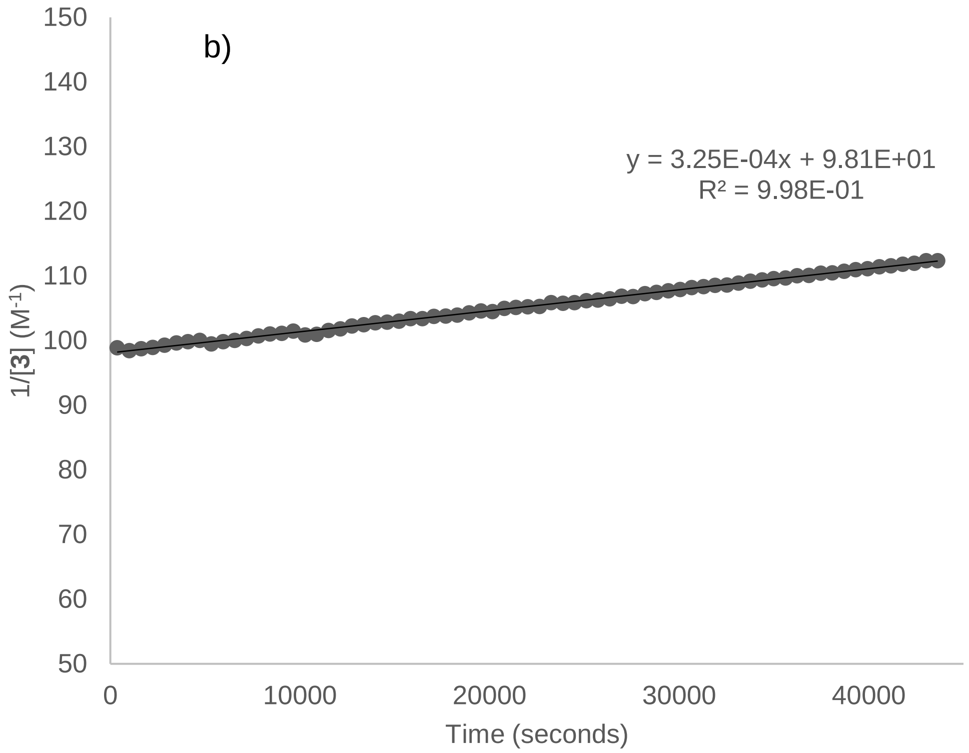

Figure S10. Kinetic data used to calculate second order rate constants for the reaction of Boc-Lys-OMe·HCl with vinyltriazine **3** in 3:7 CD_3OD/NaPi (pH 8, 50 mM in D_2O). Two measurements were taken (plots a and b) and averaged.

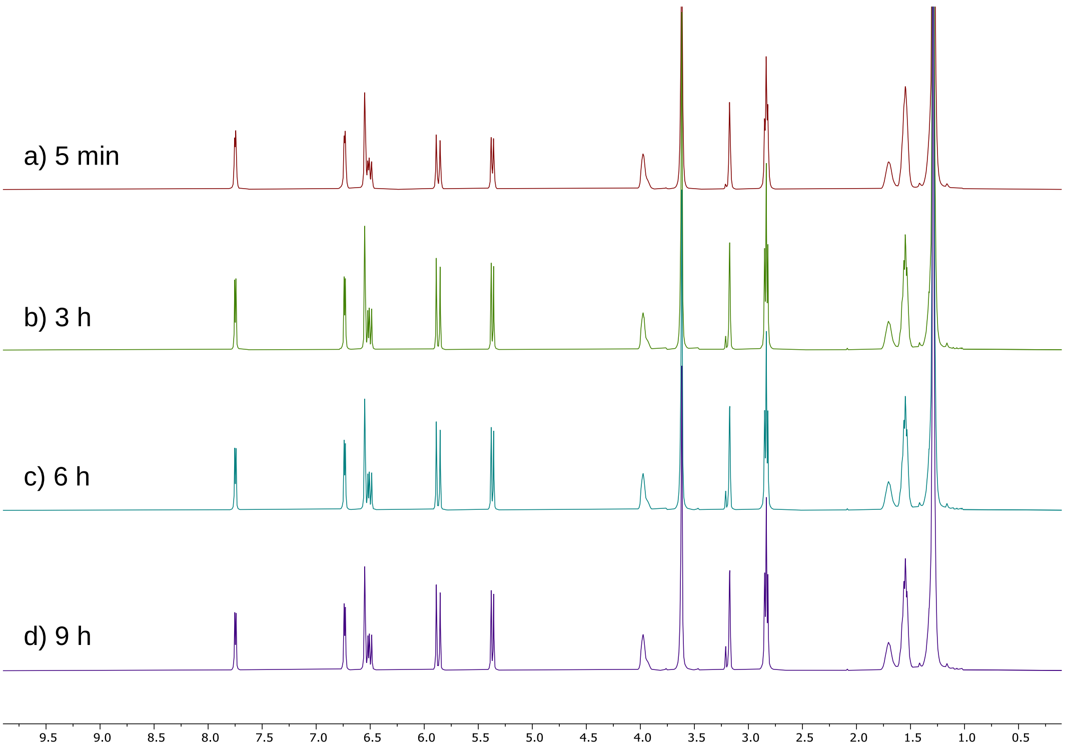

Figure S11. Representative ^{1}H NMR spectra of the reaction of vinylpyridine **1** with Boc-Lys-OMe·HCl in 3:7 CD_3OD/NaPi (pH 8, 50 mM in D_2O).

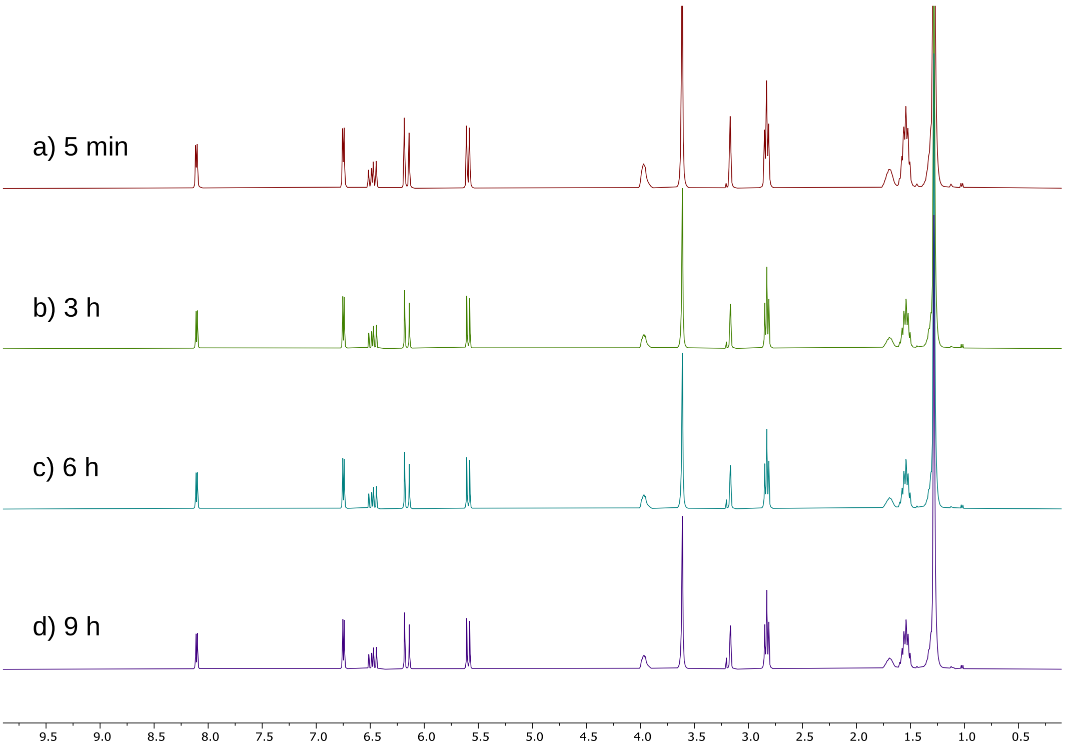

Figure S12. Representative ^{1}H NMR spectra of the reaction of vinylpyrimidine **2** with Boc-Lys-OMe·HCl in 3:7 CD_3OD/NaPi (pH 8, 50 mM in D_2O).

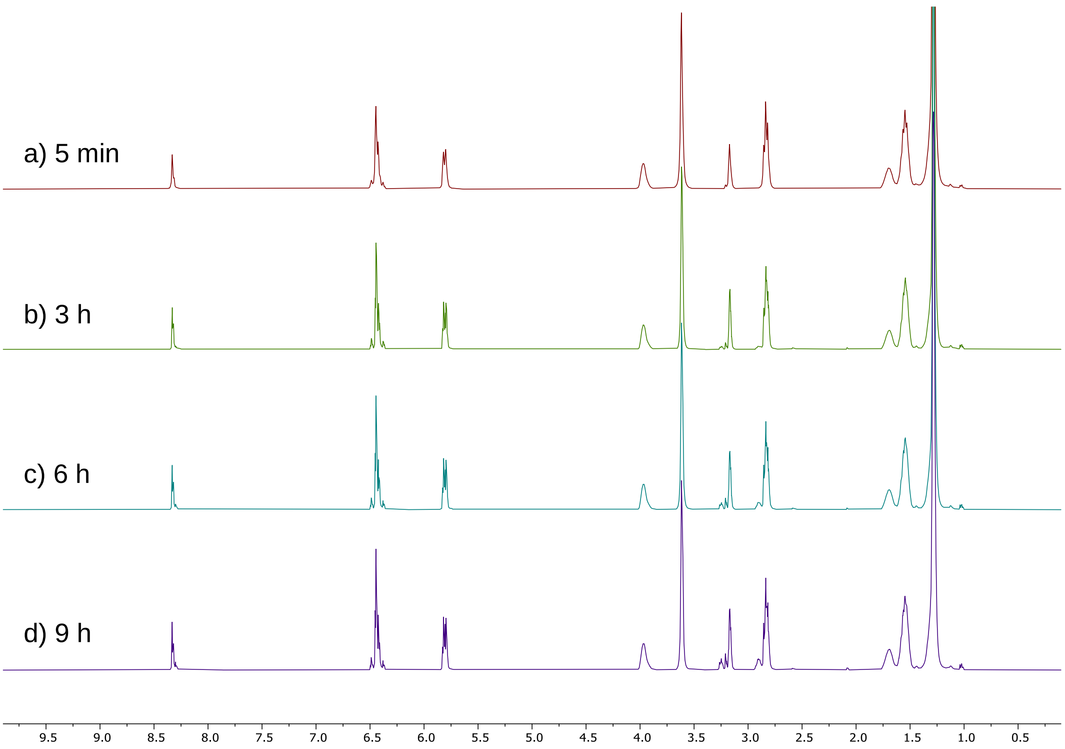

Figure S13. Representative ^{1}H NMR spectra of the reaction of vinyltriazine **3** with Boc-Lys-OMe·HCl in 3:7 CD_3OD/NaPi (pH 8, 50 mM in D_2O).

2.4. *N*-Terminus reactivity with vinylheteroarenes in CD_3OD/NaPi (pH 8, 50 mM)

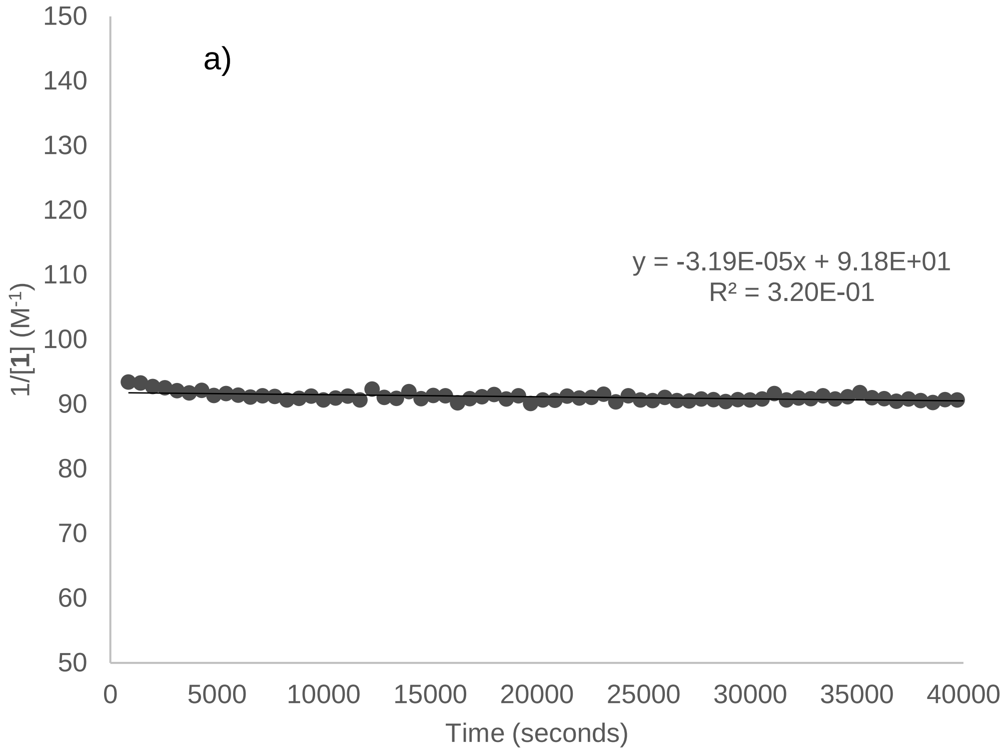

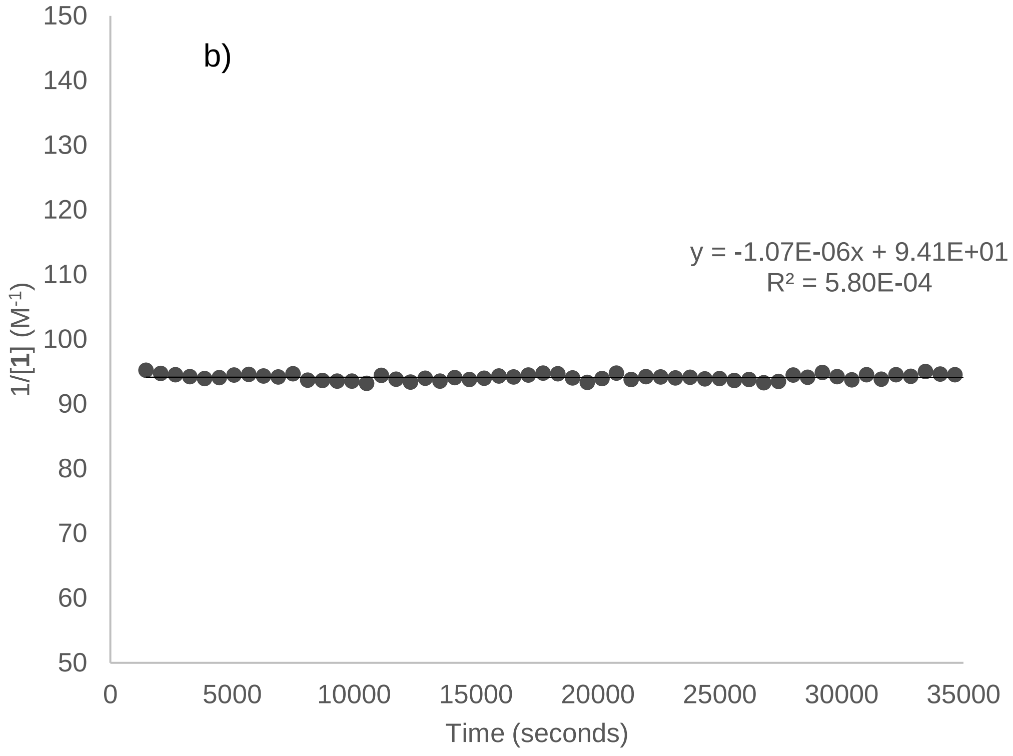

Figure S14. Kinetic data used to calculate second order rate constants for the reaction of H-Ala-NH_2·HCl with vinylpyridine **1** in 3:7 CD_3OD/NaPi (pH 8, 50 mM in D_2O). Two measurements were taken (plots a and b) and averaged.

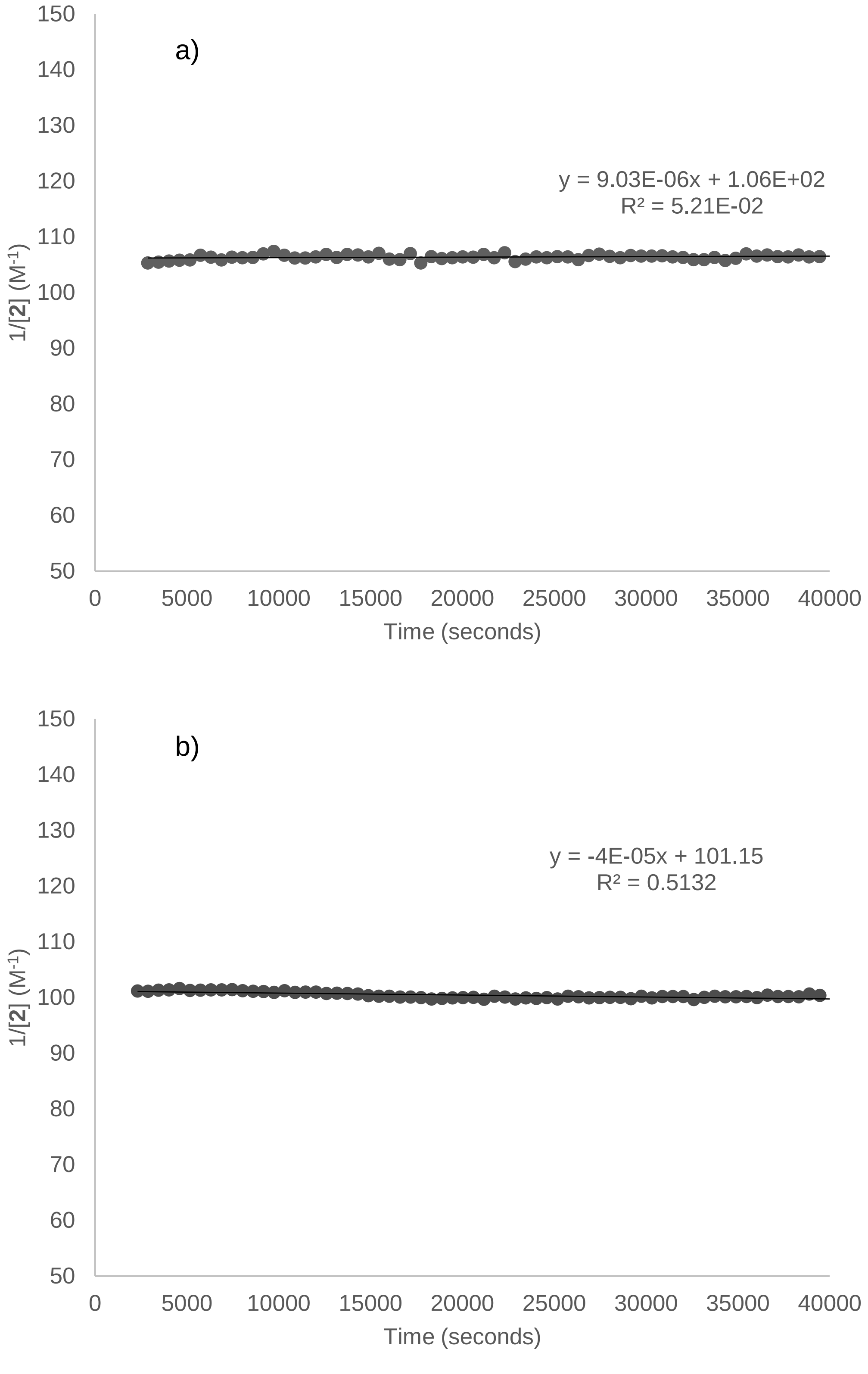

Figure S15. Kinetic data used to calculate second order rate constants for the reaction of H-Ala-NH_2·HCl with vinylpyrimidine **2** in 3:7 CD_3OD/NaPi (pH 8, 50 mM in D_2O). Two measurements were taken (plots a and b) and averaged.

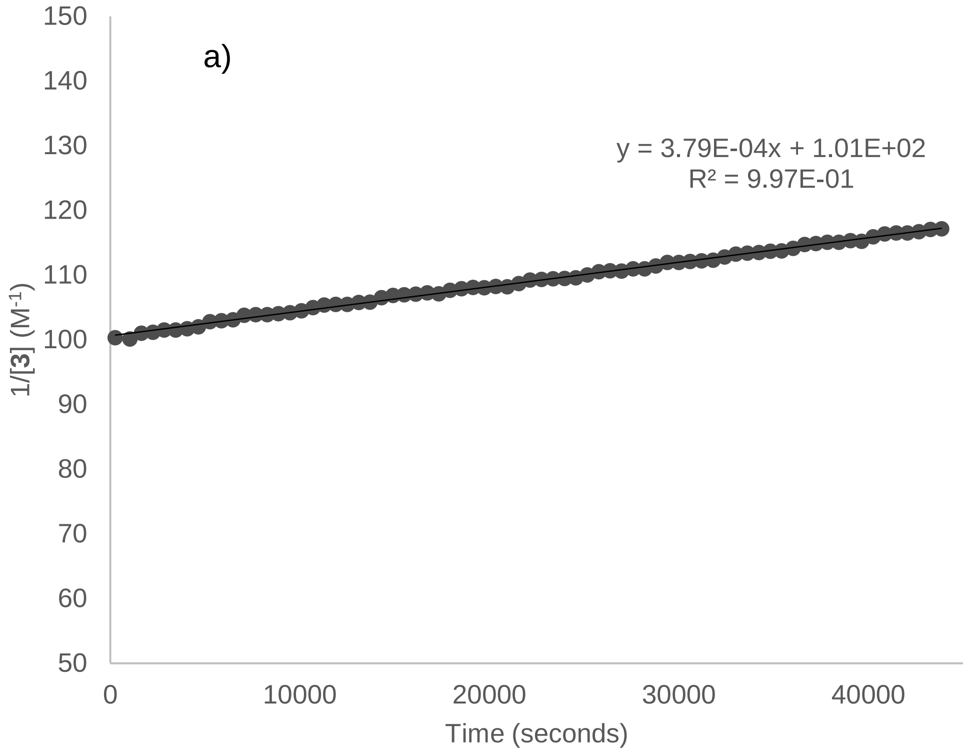

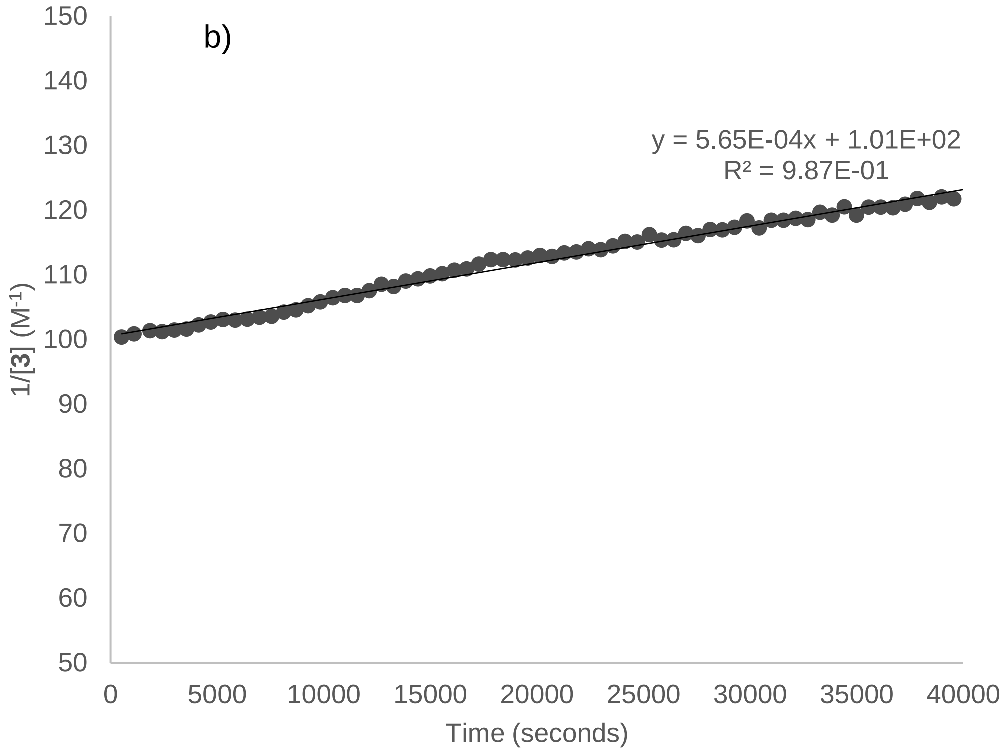

Figure S16. Kinetic data used to calculate second order rate constants for the reaction of H-Ala-NH_2·HCl with vinyltriazine **3** in 3:7 CD_3OD/NaPi (pH 8, 50 mM in D_2O). Two measurements were taken (plots a and b) and averaged.

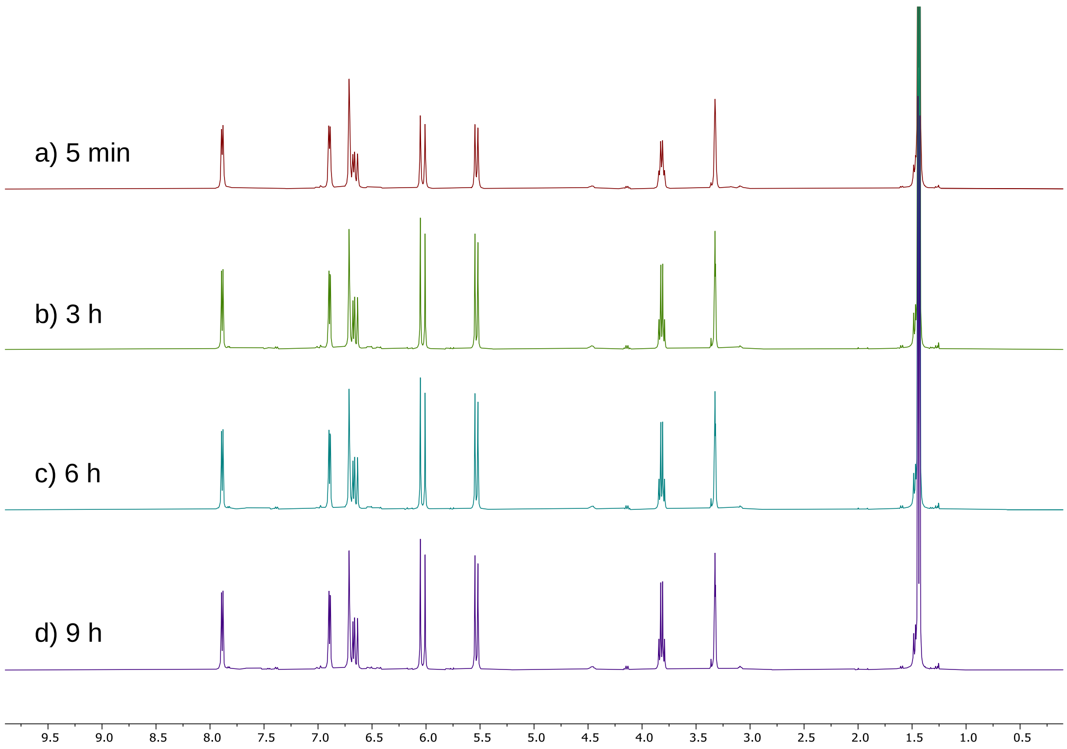

Figure S17. Representative ^{1}H NMR spectra of the reaction of vinylpyridine **1** with H-Ala-NH_2·HCl in 3:7 CD_3OD/NaPi (pH 8, 50 mM in D_2O).

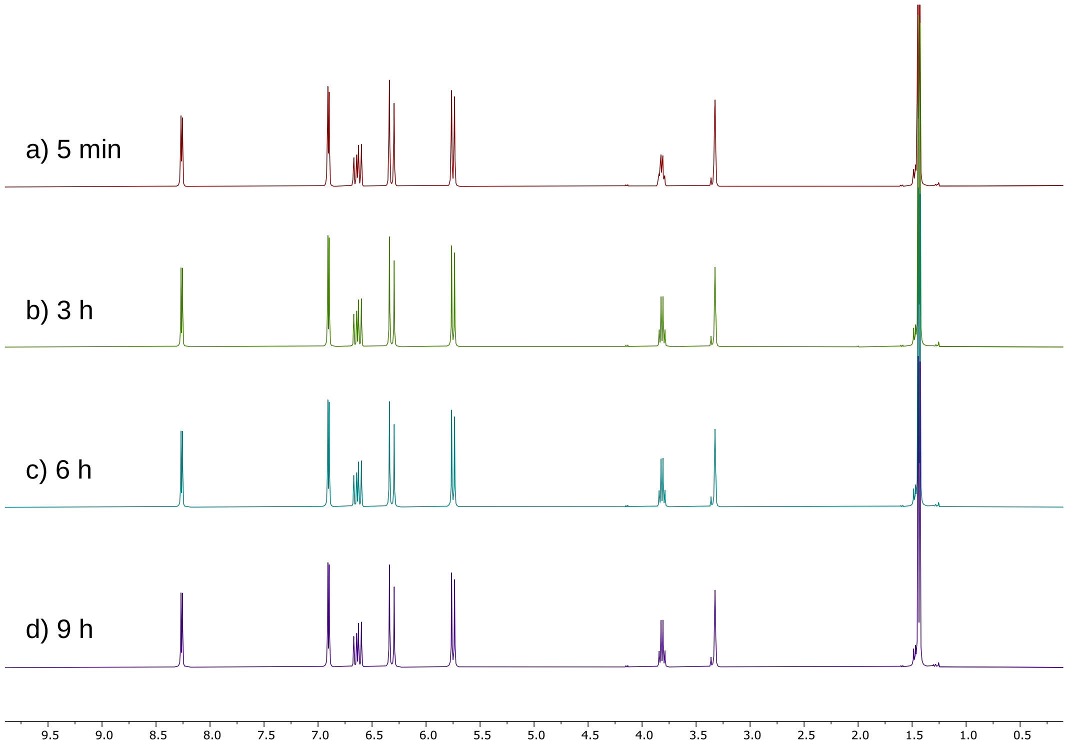

Figure S18. Representative ^{1}H NMR spectra of the reaction of vinylpyrimidine **2** with H-Ala-NH_2·HCl in 3:7 CD_3OD/NaPi (pH 8, 50 mM in D_2O).

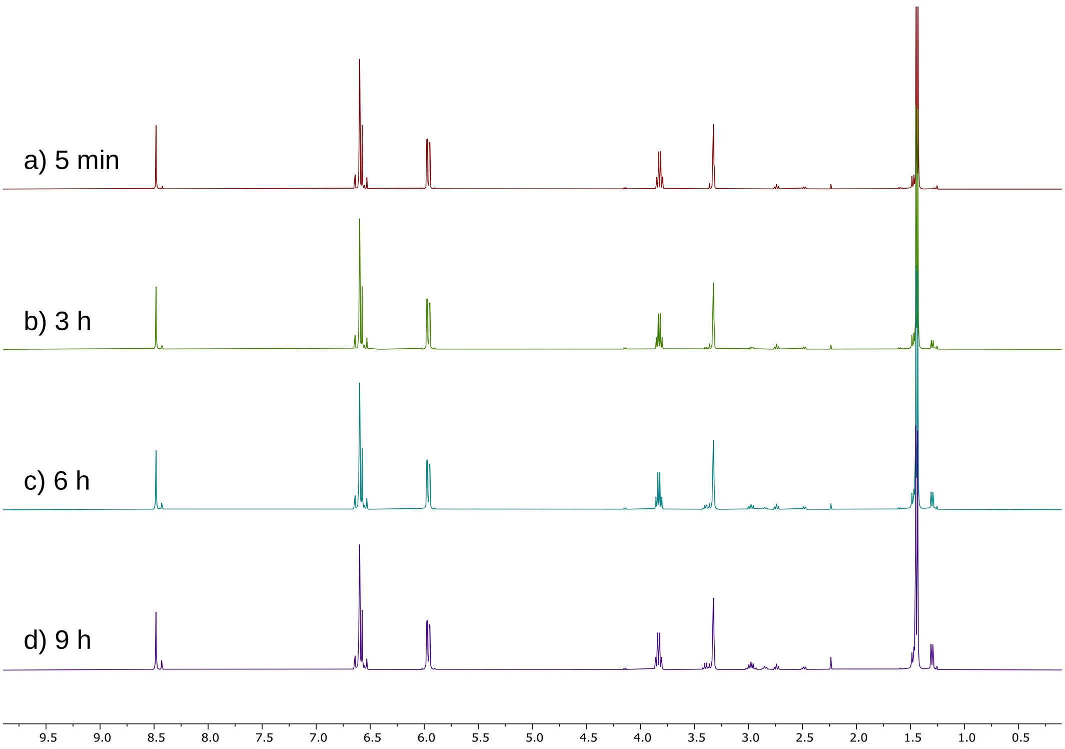

Figure S19. Representative ^{1}H NMR spectra of the reaction of vinyltriazine **3** with H-Ala-NH_2·HCl in 3:7 CD_3OD/NaPi (pH 8, 50 mM in D_2O).

2.5. Effect of buffer pH on vinylpyrimidine-cysteine reactivity

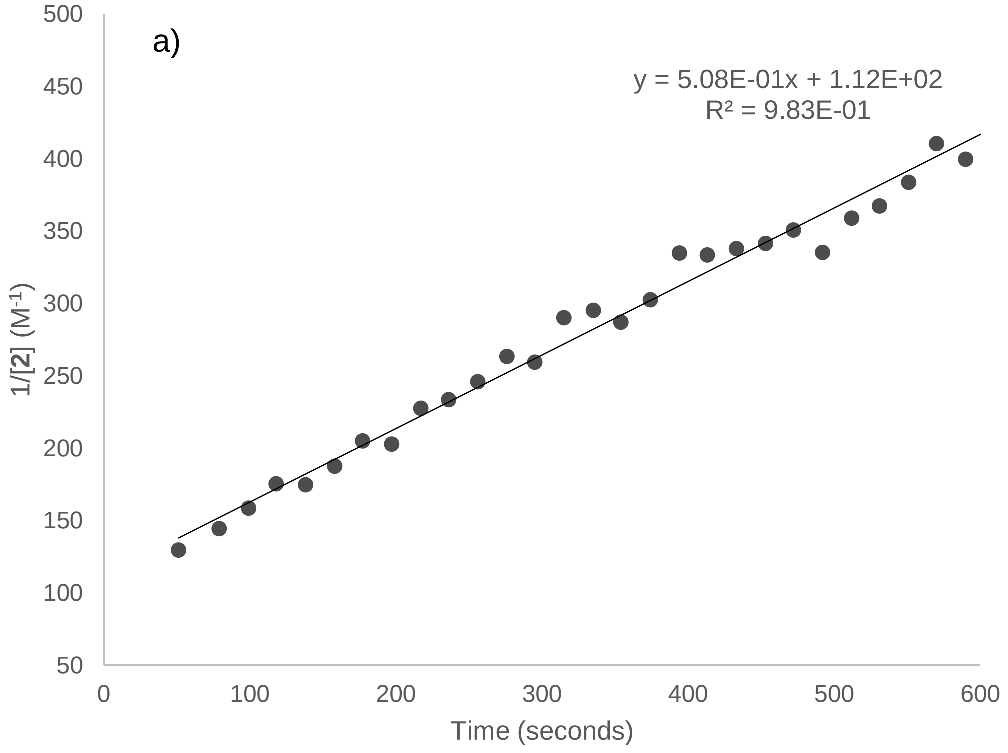

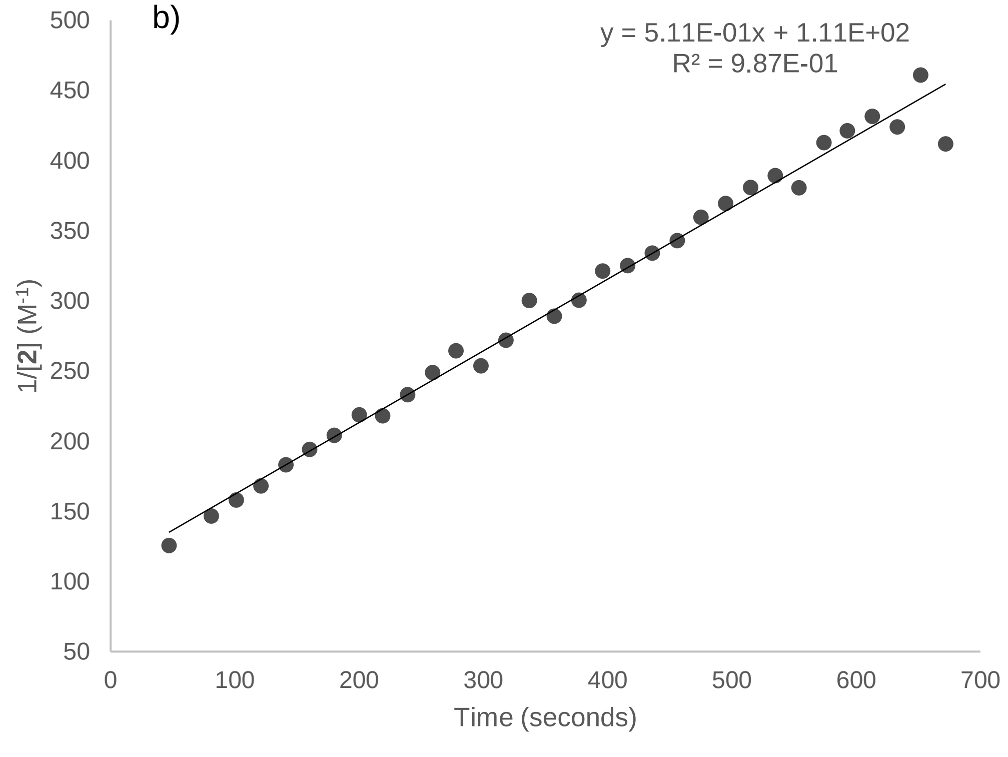

Figure S20. Kinetic data used to calculate second order rate constants for the reaction of Boc-Cys-OMe with vinylpyrimidine **2** in 3:7 CD_3OD/NaPi (pH 7, 50 mM in D_2O). Two measurements were taken (plots a and b) and averaged.

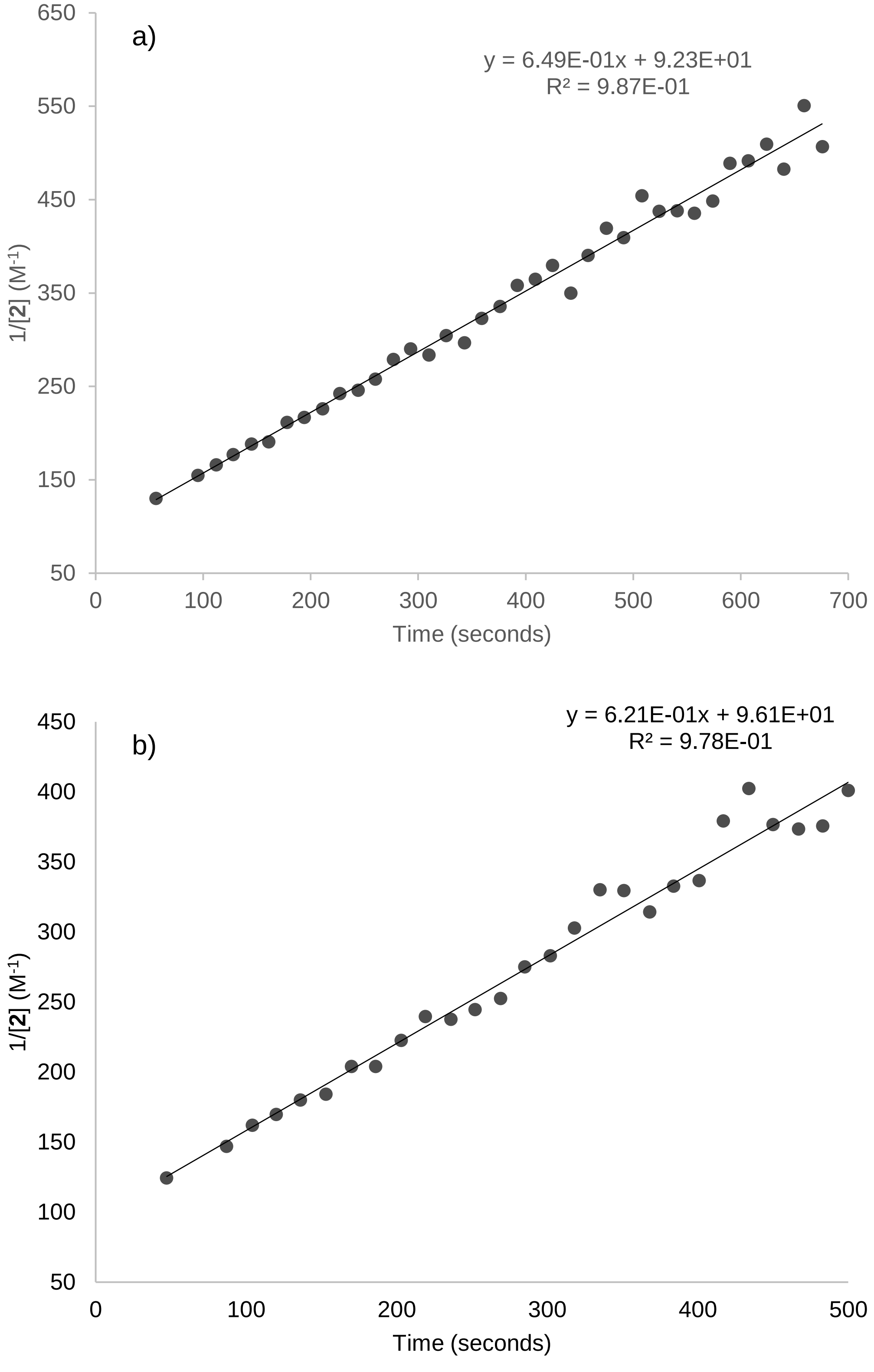

Figure S21. Kinetic data used to calculate second order rate constants for the reaction of Boc-Cys-OMe with vinylpyrimidine **2** in 3:7 CD_3OD/NaPi (pH 6, 50 mM in D_2O). Two measurements were taken (plots a and b) and averaged.

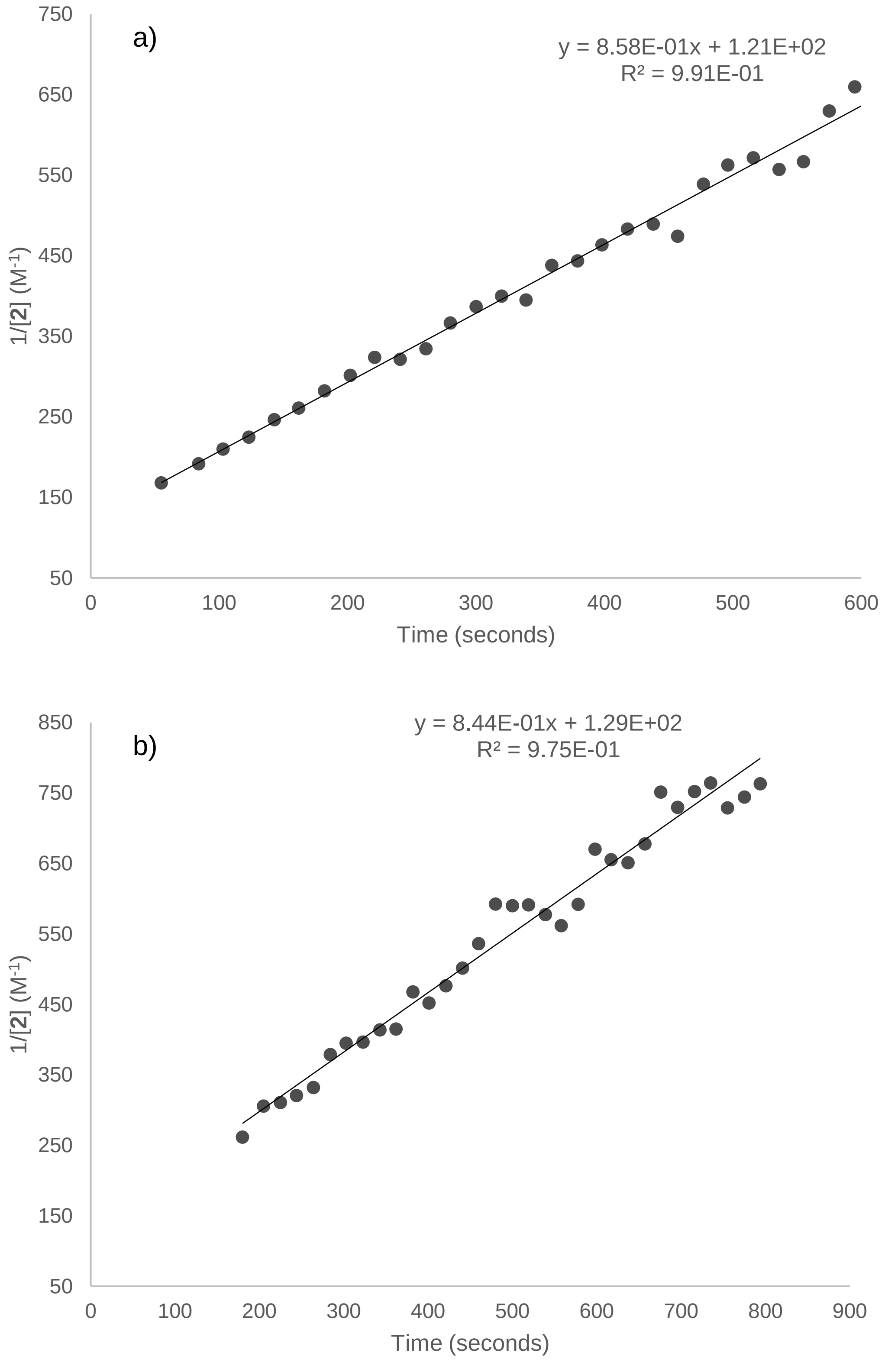

Figure S22. Kinetic data used to calculate second order rate constants for the reaction of Boc-Cys-OMe with vinylpyrimidine **2** in 3:7 CD_3OD/CD_3CO_2Na (pH 5, 50 mM in D_2O). Two measurements were taken (plots a and b) and averaged.

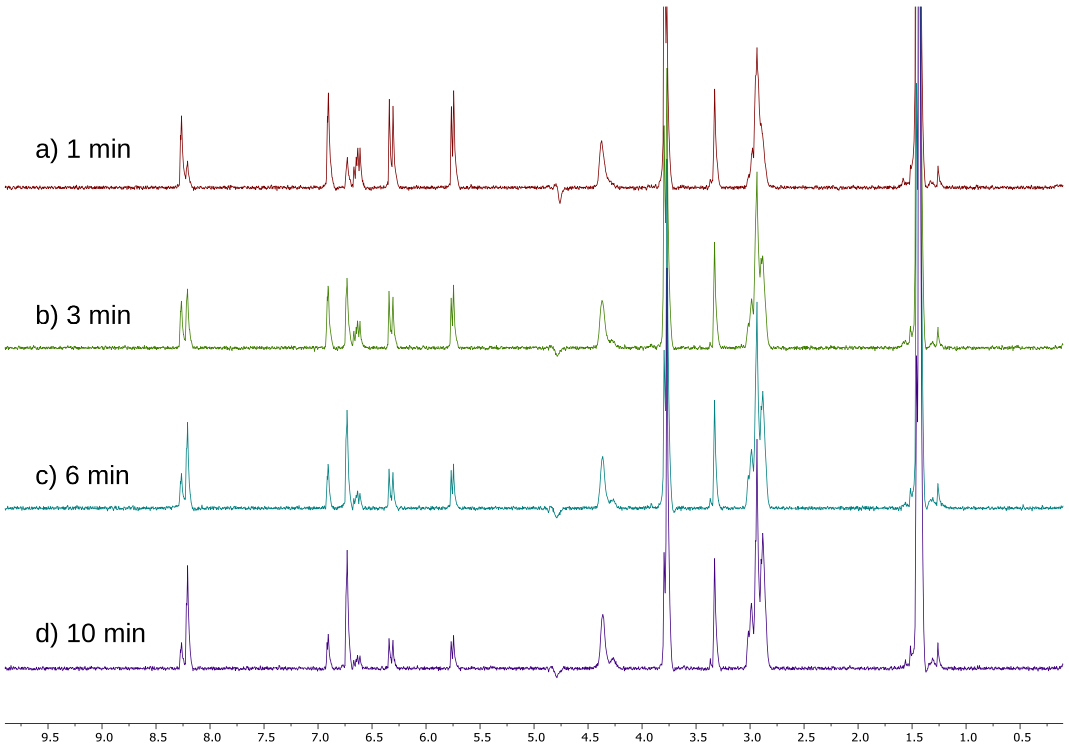

Figure S23. Representative ^{1}H NMR spectra of the reaction of vinylpyrimidine **2** with Boc-Cys-OMe in 3:7 CD_3OD/NaPi (pH 7, 50 mM in D_2O).

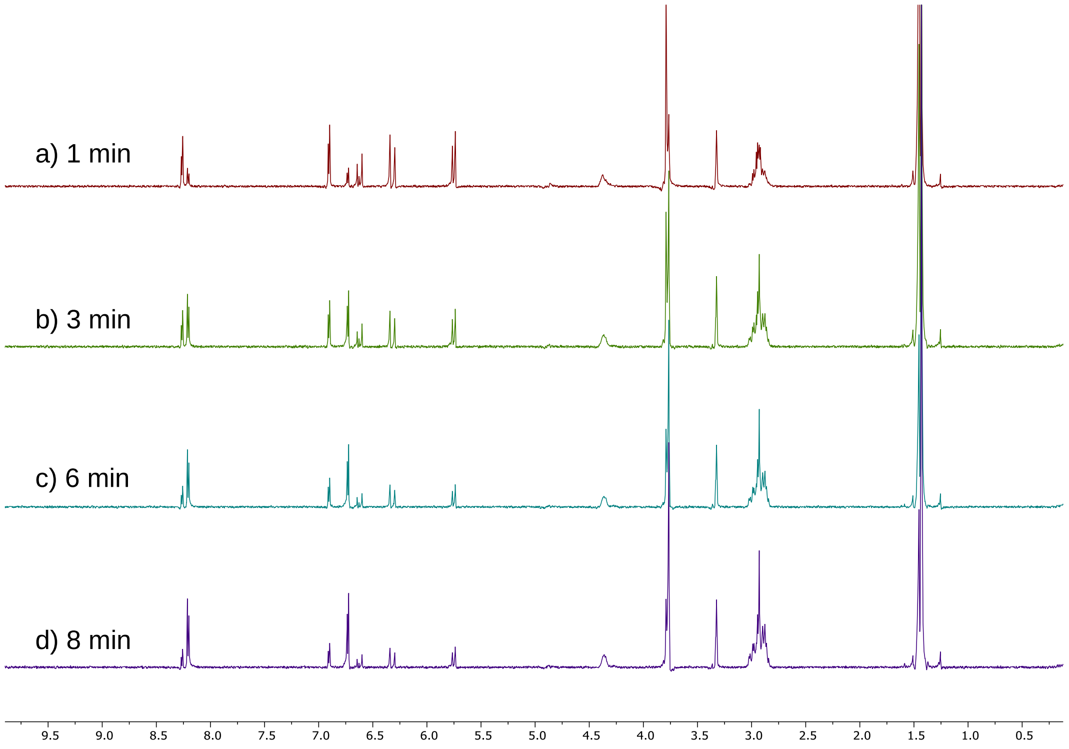

Figure S24. Representative ^{1}H NMR spectra of the reaction of vinylpyrimidine **2** with Boc-Cys-OMe in 3:7 CD_3OD/NaPi (pH 6, 50 mM in D_2O).

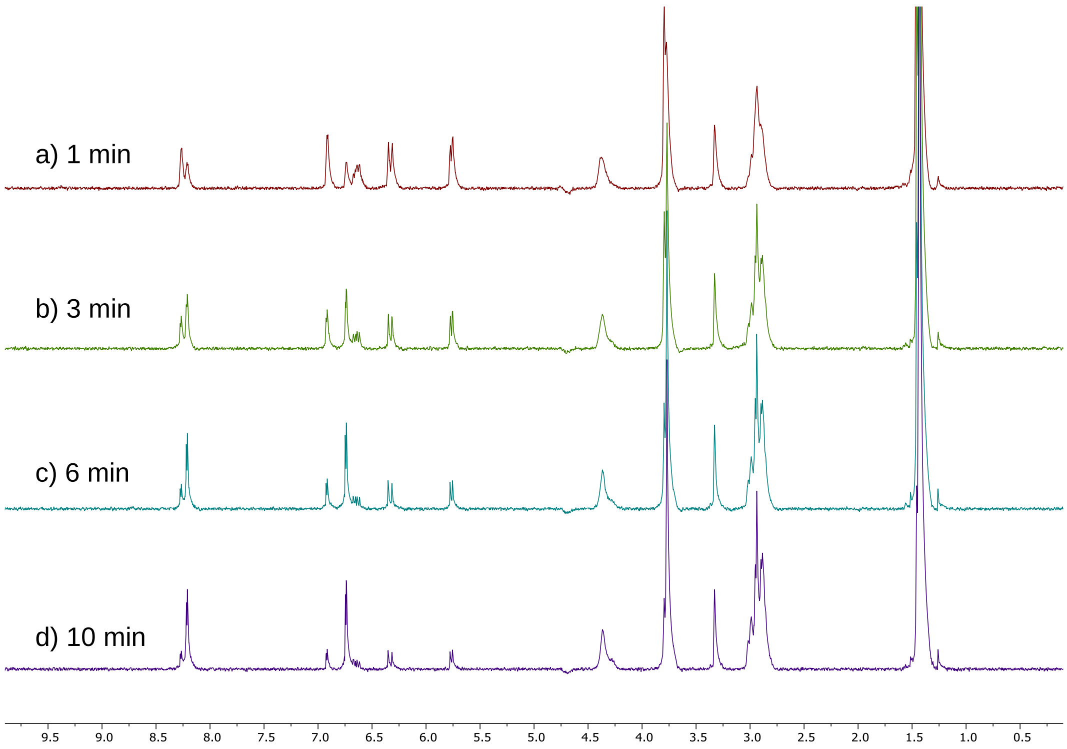

Figure S25. Representative ^{1}H NMR spectra of the reaction of vinylpyrimidine **2** with Boc-Cys-OMe in 3:7 CD_3OD/CD_3CO_2Na (pH 5, 50 mM in D_2O).

2.6. Effect of buffer concentration on vinylpyrimidine-cysteine reactivity

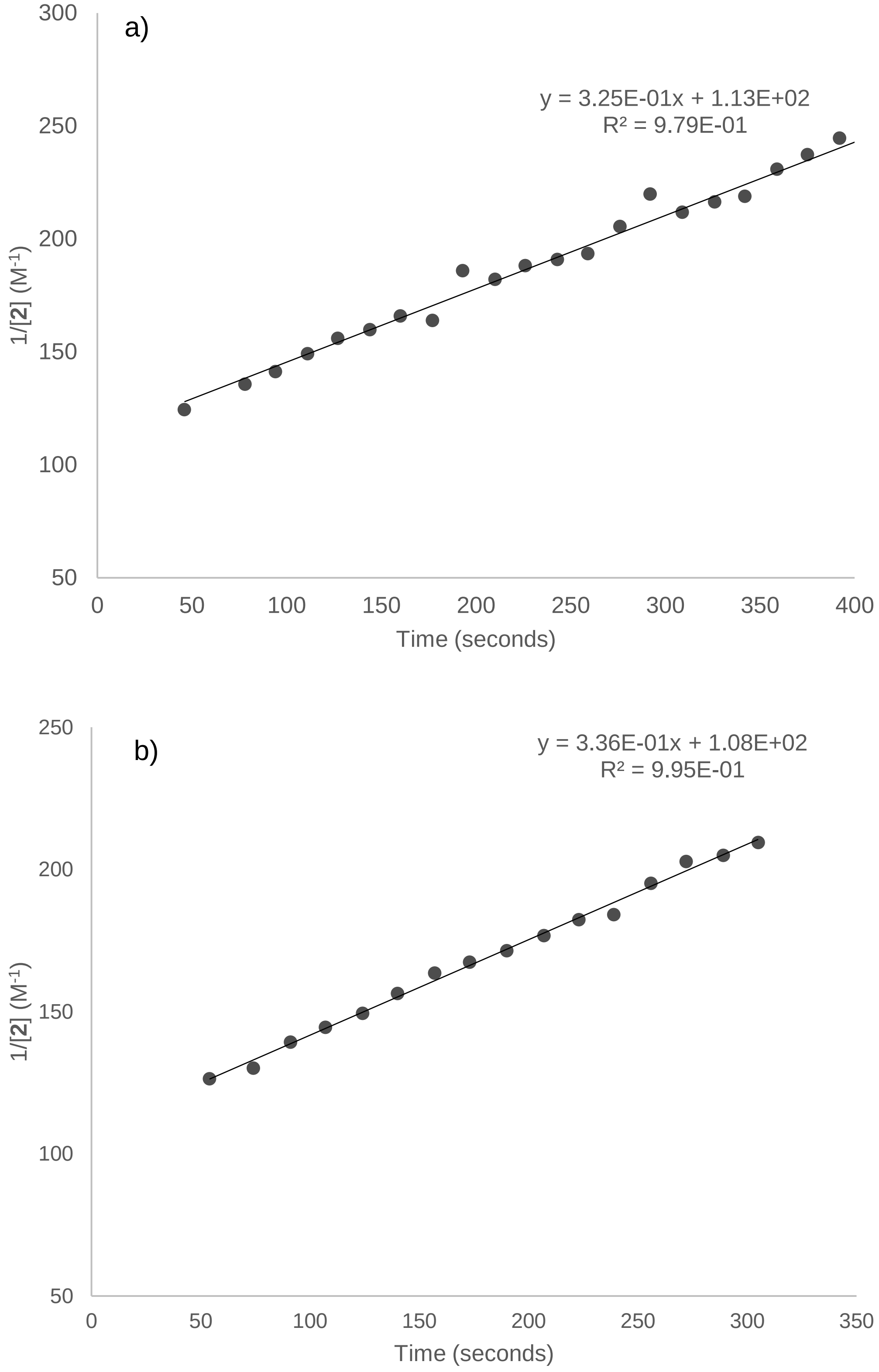

Figure S26. Kinetic data used to calculate second order rate constants for the reaction of Boc-Cys-OMe with vinylpyrimidine **2** in 3:7 CD_3OD/NaPi (pH 8, 25 mM in D_2O). Two measurements were taken (plots a and b) and averaged.

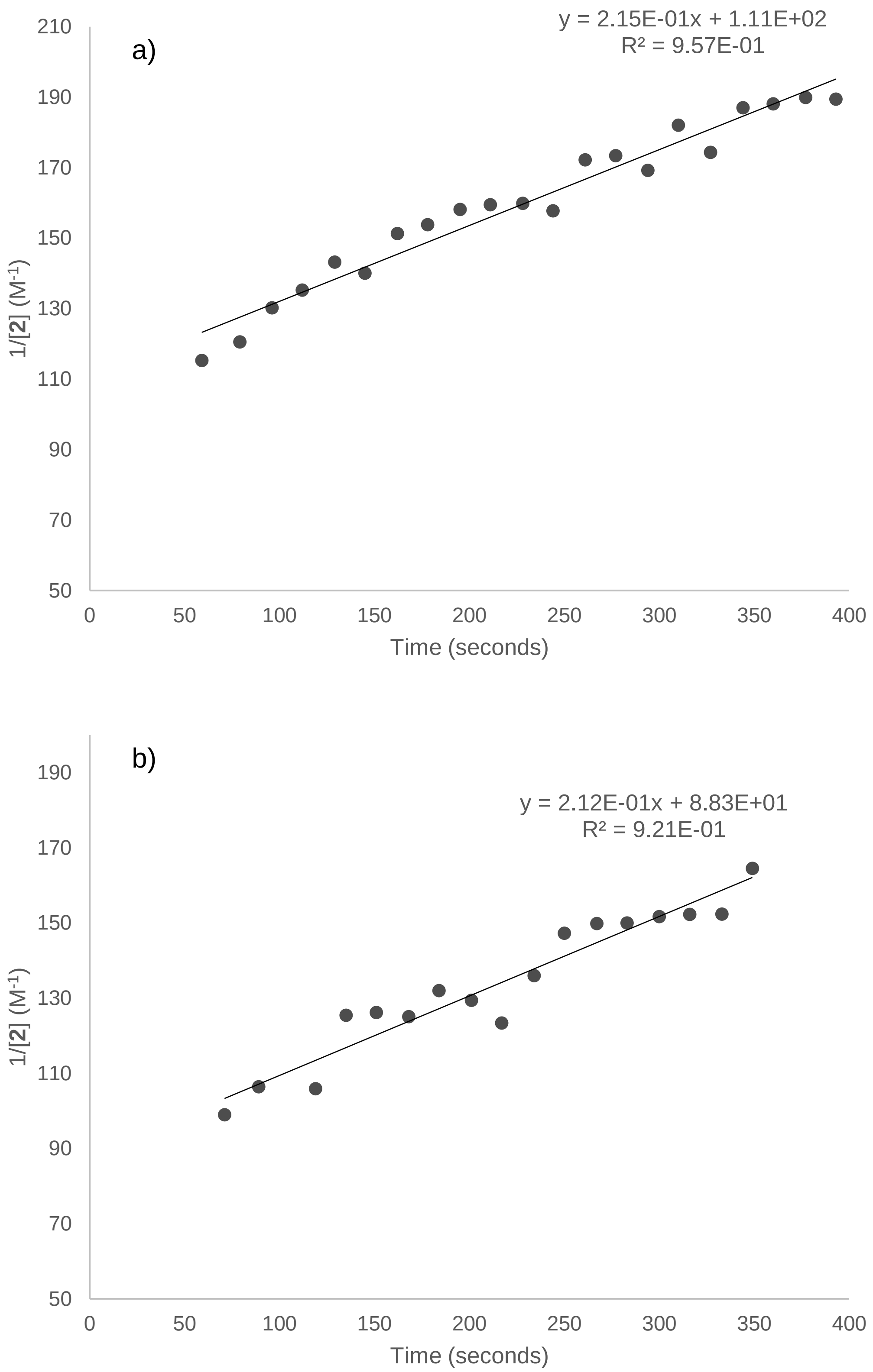

Figure S27. Kinetic data used to calculate second order rate constants for the reaction of Boc-Cys-OMe with vinylpyrimidine **2** in 3:7 CD_3OD/NaPi (pH 8, 10 mM in D_2O). Two measurements were taken (plots a and b) and averaged.

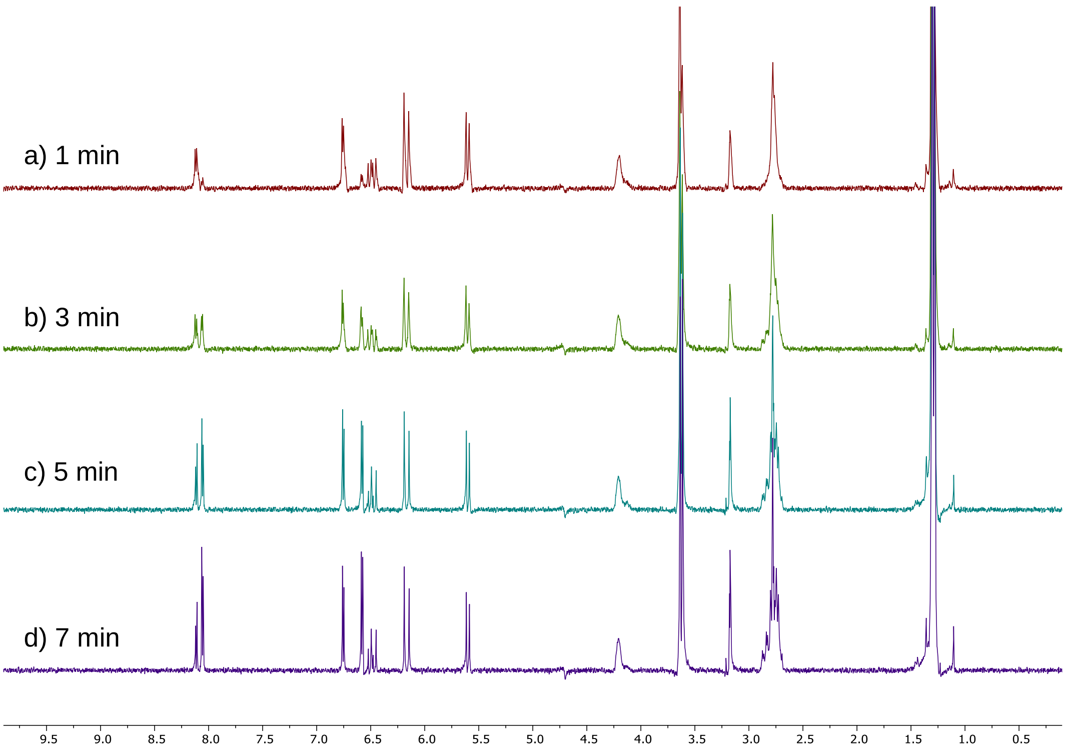

Figure S28. Representative ^{1}H NMR spectra of the reaction of vinylpyrimidine **2** with Boc-Cys-OMe in 3:7 CD_3OD/NaPi (pH 8, 25 mM in D_2O).

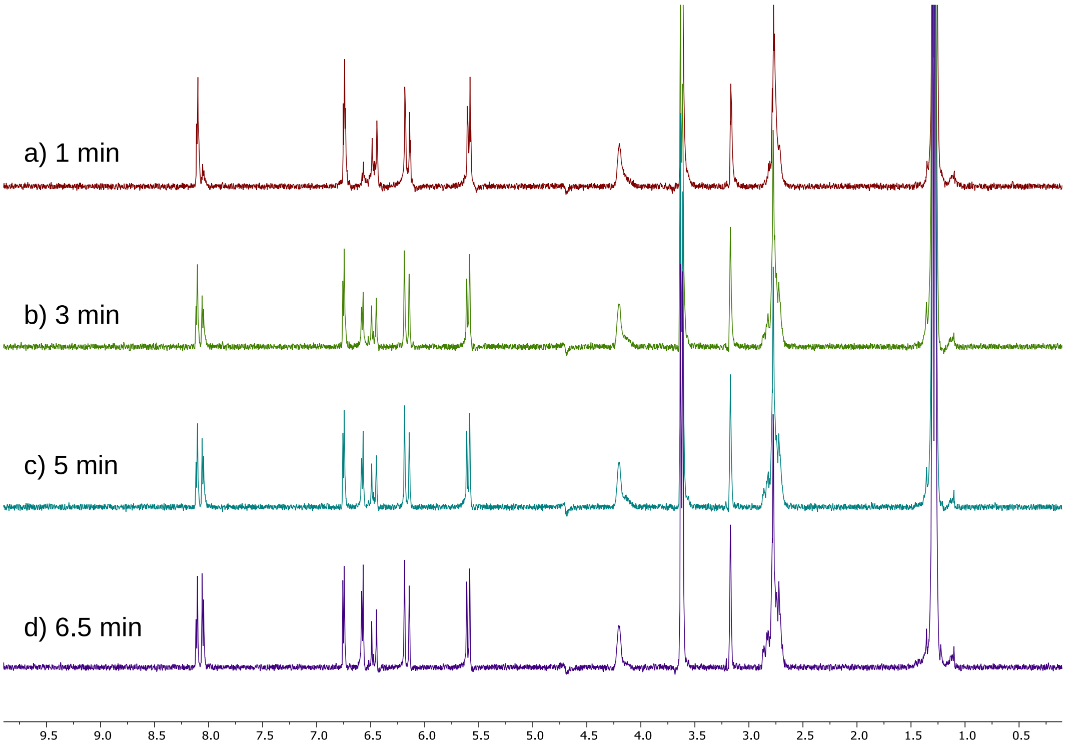

Figure S29. Representative ^{1}H NMR spectra of the reaction of vinylpyrimidine **2** with Boc-Cys-OMe in 3:7 CD_3OD/NaPi (pH 8, 10 mM in D_2O).

3. Thioether stability studies

3.1. General procedure

A solution of linker-difluorobenzyl mercaptan conjugate **5**, **6** or **7** (15 mM), 1-thioglycerol (150 mM), and sodium trifluoroacetate (4.5 mM) in 1:1 (v/v) CD_3CN/NaPi (pH 7.4, 50 mM in H_2O) was prepared. This solution was transferred to an NMR tube, and the atmosphere was purged with argon gas before the tube was sealed. The samples were placed in a water bath at 37 °C for 10 days. ^{19}F NMR spectra was acquired every day, and the integral of **5**, **6** or **7** were compared with that of an internal standard (sodium trifluoroacetate) in order to determine the amount of starting material. The CD_3CN co-solvent was required to ensure solubilisation of substrates in an aqueous solution.

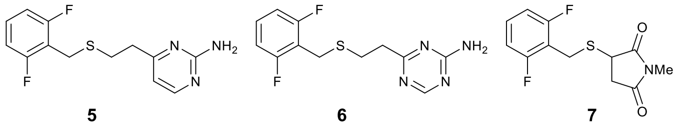

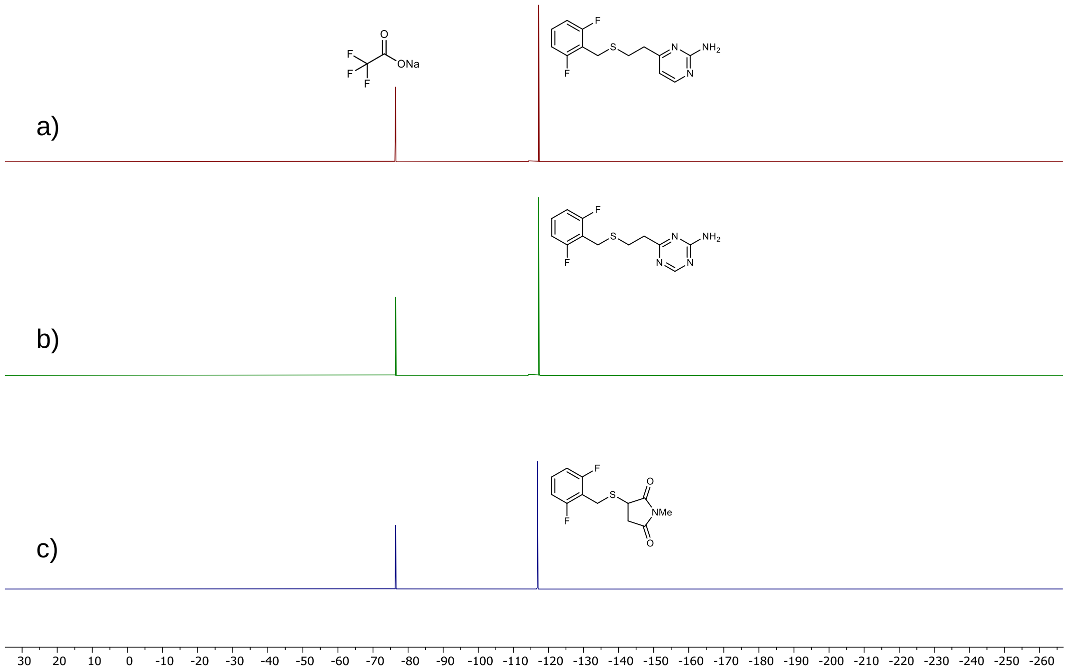

Figure S30. ^{19}F NMR spectra of incubation mixtures on Day 0 for a) pyrimidine **5**; b) triazine **6**; and c) succinimide **7**. The signal for the internal standard (sodium trifluoroacetate) can be observed at -76.5ppm.

3.2. Stability data for pyrimidine 5

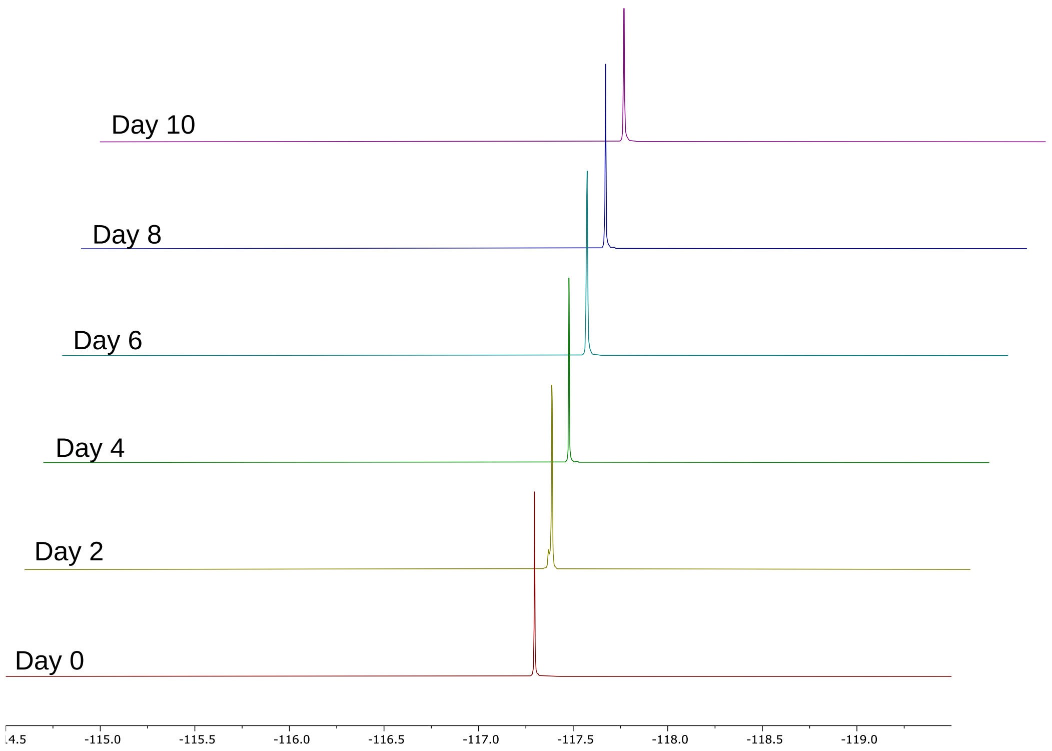

Figure S31. Representative ^{19}F NMR spectra for the stability study of pyrimidine **5**. The stacked spectra are offset by an angle of 10°.

Table S2. Integration data for the stability of pyrimidine **5**.

Day	Replicate 1				Replicate 2			
	Integral of **5**	Integral of CF_3CO_2Na	Ratio **5** vs CF_3CO_2Na	Percentage **5**	Integral of **5**	Integral of CF_3CO_2Na	Ratio **5** vs CF_3CO_2Na	Percentage **5**
0	11406	4630	2.46	100	9085	3697	2.46	100
1	9964	4086	2.44	99.0	9511	3895	2.44	99.4
2	20212	8114	2.49	101.1	19237	7736	2.49	101.2
3	10191	4150	2.46	99.7	8961	3528	2.54	103.4
4	10876	4434	2.45	99.6	10951	4325	2.53	103.0
5	9192	3727	2.47	100.1	11059	4385	2.52	102.6
6	22434	9029	2.48	100.8	22162	8887	2.49	101.5
7	11689	4751	2.46	99.9	11359	4581	2.48	100.9
8	12977	5221	2.49	100.9	13138	5294	2.48	101.0
9	12449	5065	2.46	99.8	13529	5539	2.44	99.4
10	14471	5877	2.46	99.9	11690	4759	2.46	100.0

3.3. Stability data for triazine 6

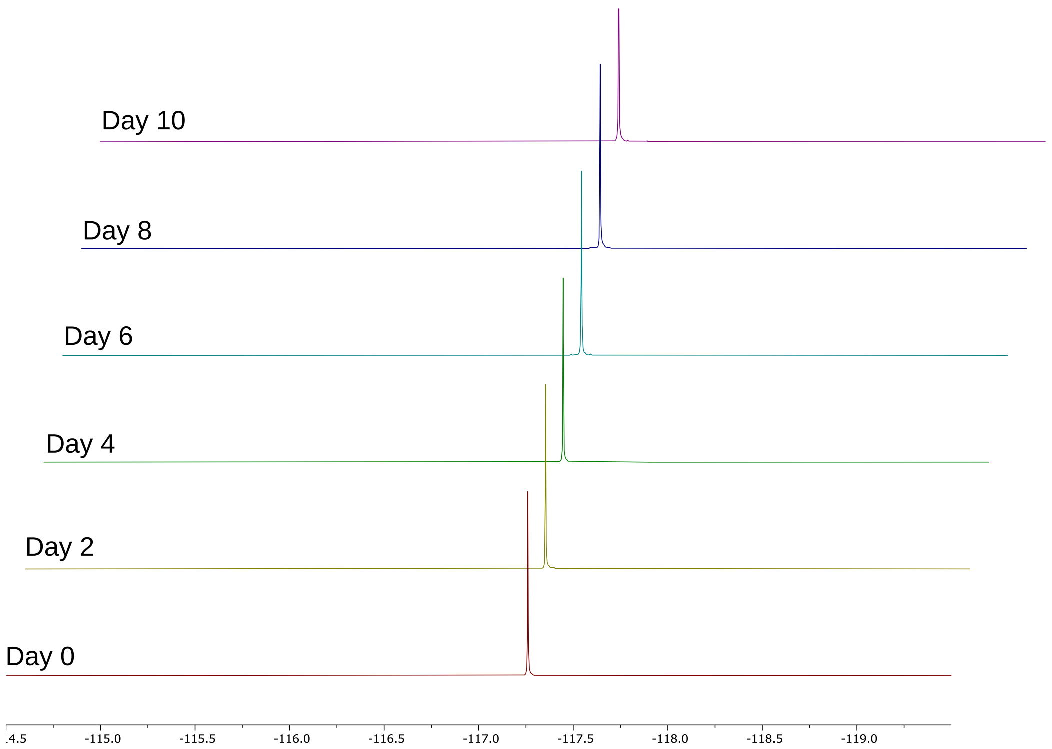

Figure S32. Representative ^{19}F NMR spectra for the stability study of triazine **6** The stacked spectra are offset by an angle of 10°.

Table S3. Integration data for the stability of triazine **6**.

Day	Replicate 1				Replicate 2			
	Integral of **6**	Integral of CF_3CO_2Na	Ratio **6** vs CF_3CO_2Na	Percentage **6**	Integral of **6**	Integral of CF_3CO_2Na	Ratio **6** vs CF_3CO_2Na	Percentage **6**
0	8720	3728	2.34	100	6350	2484	2.56	100
1	8754	3726	2.35	100.4	8928	3588	2.49	97.3
2	7325	3065	2.39	102.2	6385	2457	2.60	101.6
3	8589	3638	2.36	101.0	8476	3381	2.51	98.1
4	7388	3082	2.40	102.5	7685	3053	2.52	98.5
5	9504	4052	2.35	100.3	7573	2931	2.58	101.1
6	8766	3720	2.36	100.8	8921	3615	2.47	96.5
7	8525	3608	2.36	101.0	8992	3636	2.47	96.7
8	7174	3017	2.38	101.7	9028	3609	2.50	97.8
9	14606	6310	2.31	99.0	9687	3947	2.45	96.0
10	14365	6267	2.29	98.0	9635	3968	2.43	95.0

3.4. Stability data for succinimide 7

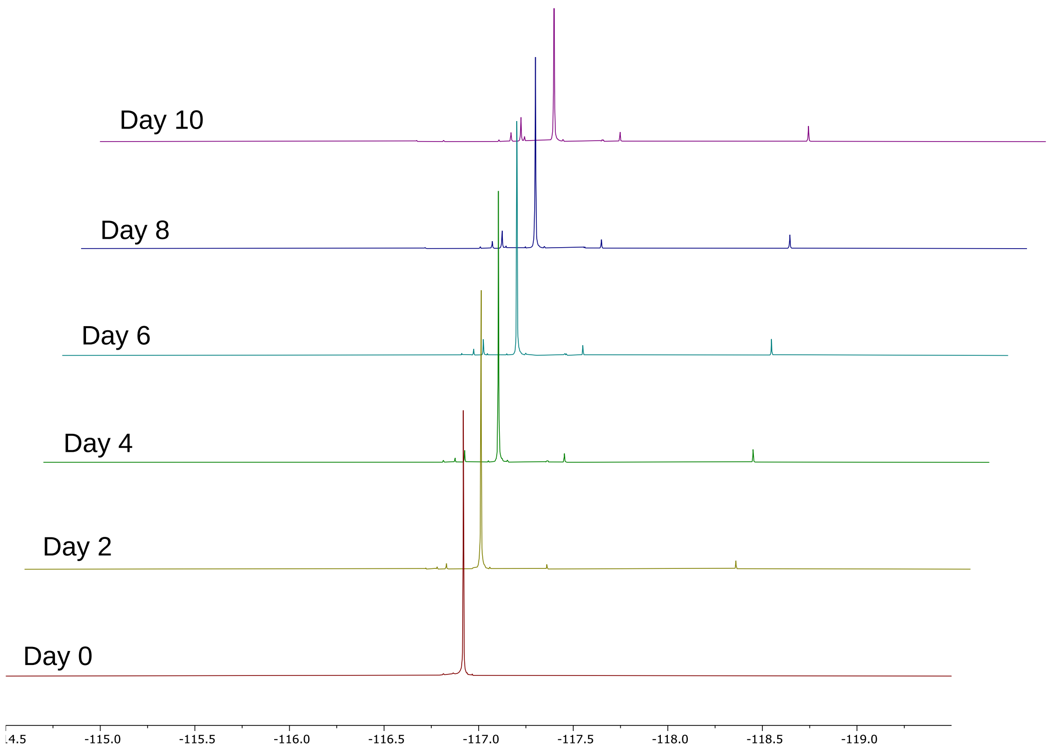

Figure S33. Representative ^{19}F NMR spectra for the stability study of succinimide **7**. The stacked spectra are offset by an angle of 10°. A number of new fluorinated peaks emerged over the ten-day incubation period.

Table S4. Integration data for the stability of succinimide **7**.

Day	Replicate 1				Replicate 2			
	Integral of **7**	Integral of CF_3CO_2Na	Ratio **7** vs CF_3CO_2Na	Percentage **7**	Integral of **7**	Integral of CF_3CO_2Na	Ratio **7** vs CF_3CO_2Na	Percentage **7**
0	7705	3185	2.42	100	8714	3701	2.35	100
1	8487	3694	2.30	95.0	8344	3707	2.25	95.6
2	7809	3593	2.17	89.9	7743	3659	2.12	89.9
3	7215	3520	2.05	84.7	6170	3060	2.02	85.7
4	7059	3599	1.96	81.1	5905	3073	1.92	81.6
5	5741	3085	1.86	76.9	6101	3628	1.68	71.4
6	6576	3687	1.78	73.7	6582	3813	1.73	73.3
7	5255	3031	1.73	71.7	6332	3823	1.66	70.3
8	6133	3702	1.66	68.5	6184	3876	1.60	67.8
9	6320	4038	1.57	64.7	6300	4198	1.50	63.8
10	5962	4012	1.49	61.4	4041	2737	1.48	62.7

4. Modification of human serum albumin

4.1. Reduction of human serum albumin

The purity of commercially available recombinant human serum albumin (HSA; purchased from Sigma Aldrich; product code A9731) was determined by protein LCMS. This revealed a mixture of the free Cys-34 form (66445 Da) and the cysteinylated form (i.e. disulfide bonded with cysteine; 66566 Da).

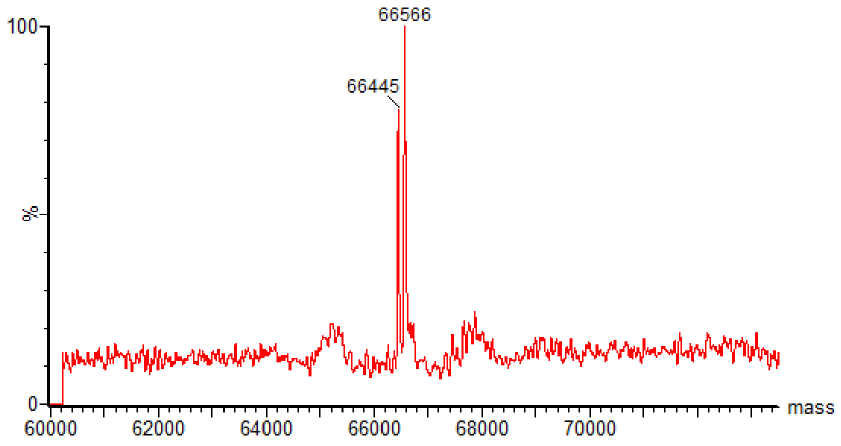

Figure S34. Deconvoluted mass spectrum of commercially available HSA. The mass difference between the two signals corresponds to cysteinylation of the Cys-34 residue. Expected mass difference from cysteinylation: 119 Da; observed mass difference: 121 Da.

A sample of the free Cys-34 HSA was required for bioconjugation. This was achieved *via* reduction with dithiothreitol (DTT).

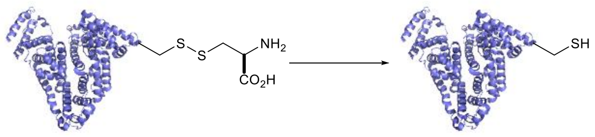

Dithiothreitol (DTT, 1.81 µL, 5 mM in PBS (×1)) was added to a solution of HSA (17.9 µL, 253 µM) in PBS (100 µL, ×1)). The resulting solution was vortexed, and incubated at 37 °C for 2 h. Removal of excess reagents and buffer exchange to the required solvent was achieved by repeated ultracentrifugation into PBS (×1) or Tris·HCl (pH 8, 50 mM) using an Amicon-Ultra centrifugal filter (10k MWCO, Merck Millipore).

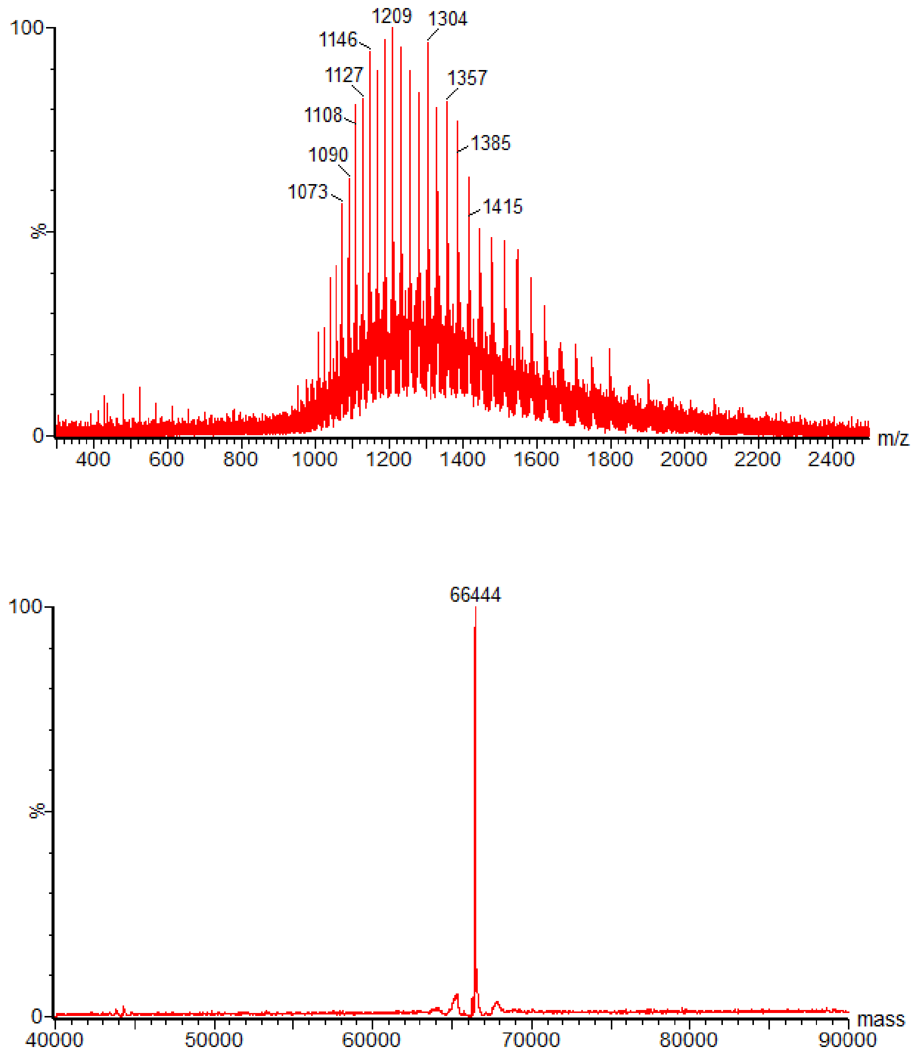

Figure S35. Non-deconvoluted (top) and deconvoluted (bottom) mass spectrum of the free Cys-34 form of HSA.

4.2. Optimisation for the reaction between HSA and vinylpyrimidine 2

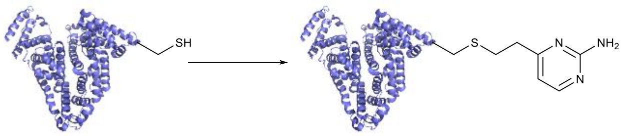

Table S5. Screening of conjugation reactions between HSA (free Cys-34 form) and vinylpyrimidine **2**. Reactions were performed at 37 °C in buffer solutions containing 5% DMSO. Aliquots of the reaction mixture were taken and quenched using 1-thioglycerol at the required timepoints prior to LCMS analysis. Percentage conversion reported to the nearest 5%. Refer to following sections for a representative experimental.

Entry	[HSA]	Linker eq.	Percentage conversion							
			PBS (×1)				Tris·HCl (pH 8, 50 mM)			
			1 h	2 h	3 h	4 h	1 h	2 h	3 h	4 h
1	35 μM	5	30	40	50	60	40	60	70	75
2		10	45	65	70	75	50	70	75	**>95**
3		20	65	75	75	80	75	**>95**	-	-
4	10 μM	20	35	45	55	65	50	70	75	**>95**
5		50	55	70	70	75	75	**>95**	-	-

4.3. Synthesis of HSA-2 using optimised conditions

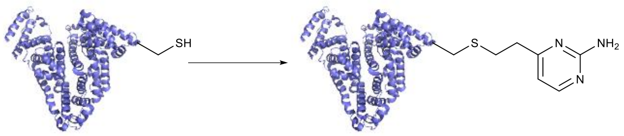

Linker **2** (0.77 µL, 20 mM in DMSO), Tris·HCl (18.8 µL, pH 8, 50 mM) and DMSO (0.33 µL) were added to a solution of HSA (Cys-34 free form, 2.0 µL, 384 µM in Tris·HCl (pH 8, 50mM)). After incubation at 37 °C for 2 h, the solution was purified *via* Zeba Spin desalting column (7k MWCO, ThermoFisher, pre-equilibrated with Tris·HCl (pH 8, 50 mM)) to give **HSA-2**.

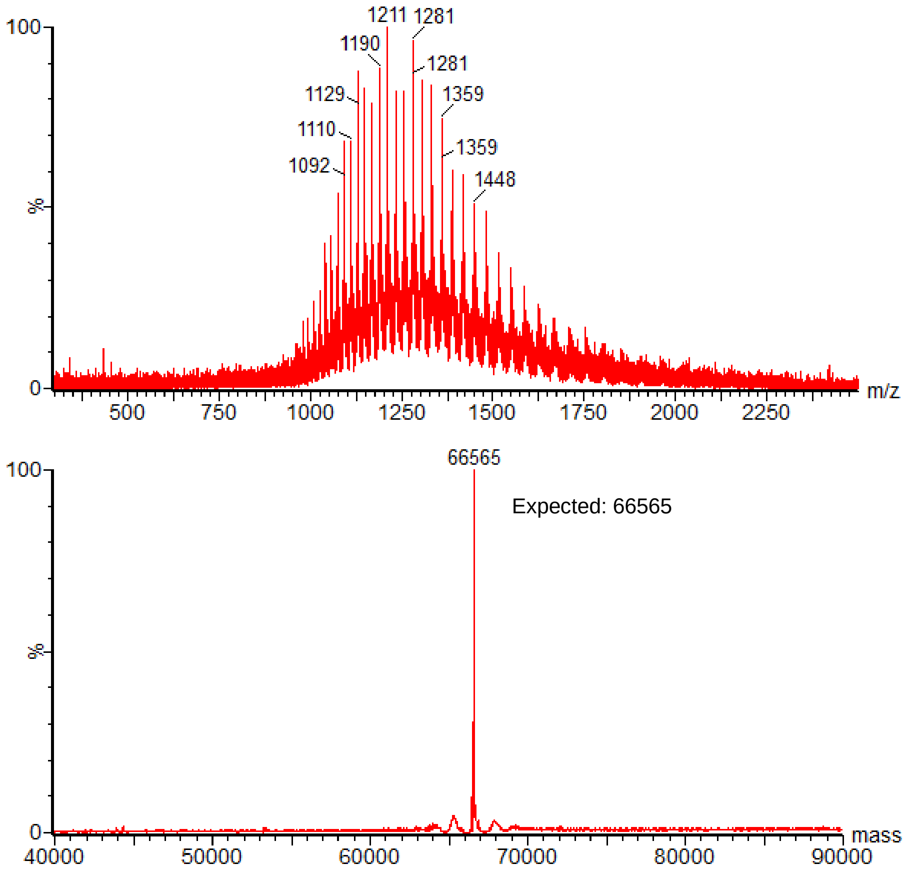

Figure S36. Non-deconvoluted (top) and deconvoluted (bottom) mass spectrum of **HSA-2**. Expected 66565 Da; observed 66565 Da.

4.4. Synthesis of HSA-8

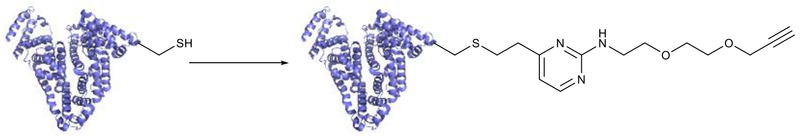

Linker **8** (16.8 µL, 20 mM in DMSO), Tris·HCl (413 µL, pH 8, 50 mM) and DMSO (7.15 µL) were added to a solution of HSA (Cys-34 free form, 43.9 µL, 384 µM in Tris·HCl (pH 8, 50mM)). After incubation at 37 °C for 2 h, the solution was purified *via* Zeba Spin desalting column (7k MWCO, ThermoFisher, pre-equilibrated with Tris·HCl (pH 8, 50 mM)) to give **HSA-8**.

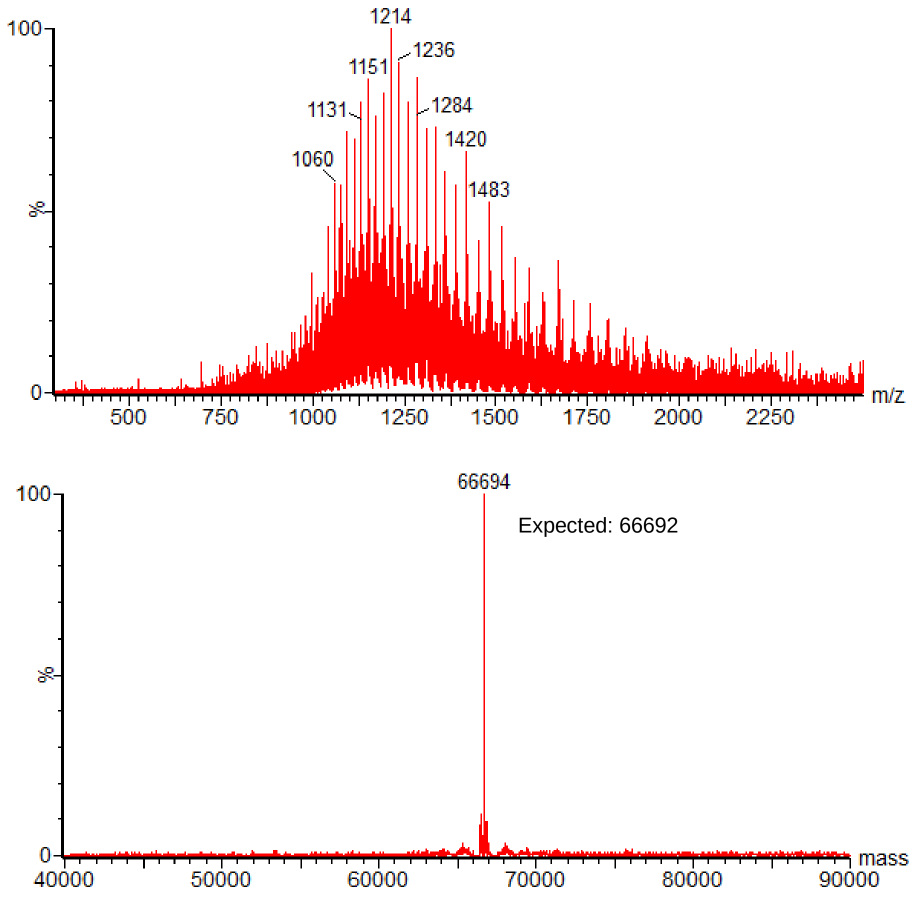

Figure S37. Non-deconvoluted (top) and deconvoluted (bottom) mass spectrum of **HSA-8**. Expected 66692 Da; observed 66694 Da.

4.5. Synthesis of HSA-9

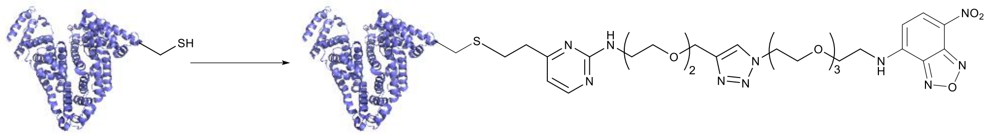

Linker **9** (0.58 µL, 20 mM in DMSO), Tris·HCl (14.1 µL, pH 8, 50 mM) and DMSO (0.25 µL) were added to a solution of HSA (Cys-34 free form, 1.5 µL, 384 µM in Tris·HCl (pH 8, 50 mM)). After incubation at 37 °C for 2 h in the dark, the solution was purified *via* Zeba Spin desalting column (7k MWCO, ThermoFisher, pre-equilibrated with Tris·HCl (pH 8, 50 mM)) to give **HSA-9**.

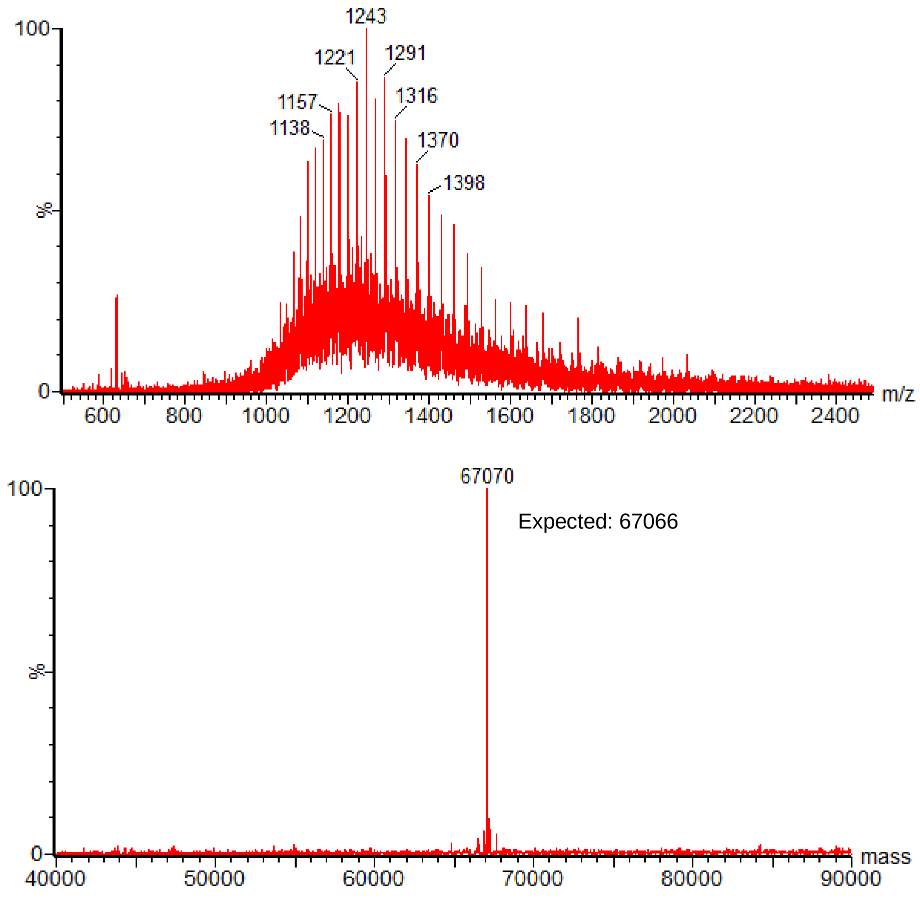

Figure S38. Non-deconvoluted (top) and deconvoluted (bottom) mass spectrum of **HSA-9**. Expected 67066 Da; observed 67070 Da.

4.6. Synthesis of HSA-10

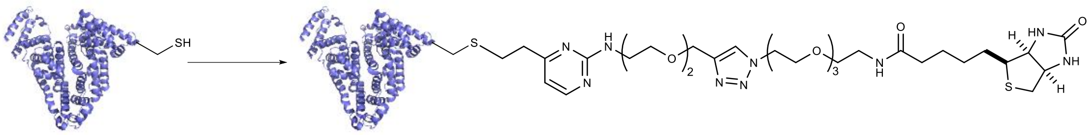

Linker **10** (0.58 µL, 20 mM in DMSO), Tris·HCl (14.1 µL, pH 8, 50 mM) and DMSO (0.25 µL) were added to a solution of HSA (Cys-34 free form, 1.5 µL, 384 µM in Tris·HCl (pH 8, 50mM)). After incubation at 37 °C for 2 h, the solution was purified *via* Zeba Spin desalting column (7k MWCO, ThermoFisher, pre-equilibrated with Tris·HCl (pH 8, 50 mM)) to give **HSA-10**.

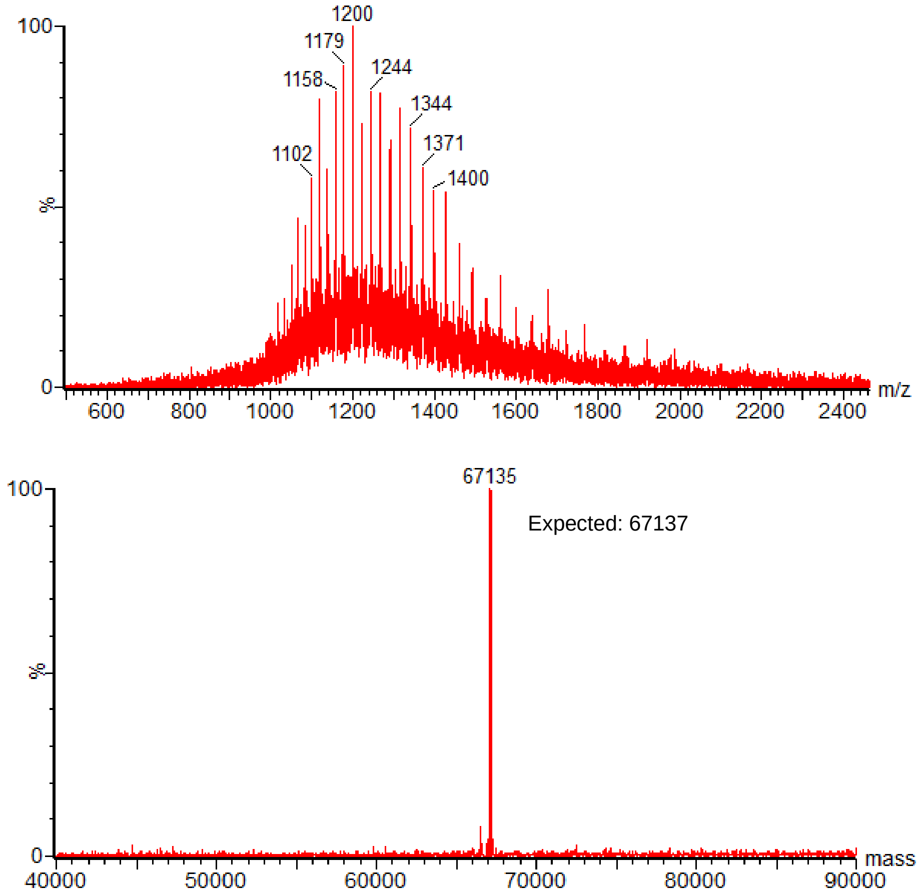

Figure S39. Non-deconvoluted (top) and deconvoluted (bottom) mass spectrum of **HSA-10**. Expected 67137 Da; observed 67135 Da.

4.7. Optimisation for the reaction between HSA and vinyltriazine 3

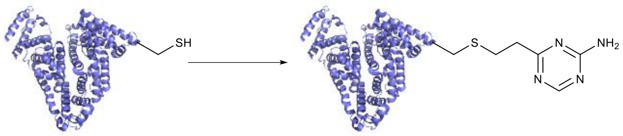

Table S6. Screening of conjugation reactions between HSA (free Cys-34 form) and vinyltriazine **3**. Reactions were performed at 37 °C in buffer solutions containing 5% DMSO. Aliquots of the reaction mixture were taken and quenched using 1-thioglycerol at the required timepoints prior to LCMS analysis. Percentage conversion reported to the nearest 5%. Refer to following sections for a representative experimental.

Entry	[HSA]	Linker eq.	Percentage conversion					
			PBS (×1)		Tris·HCl (pH 8)			
			30 min	1 h	30 min	1 h	2 h	3 h
1	35 μM	1	<5	25	<5	<5	-	-
2		2	<5	40	<5	25	-	-
3		5	<5	45	35	45	-	-
4		10	35	70	55	70	-	-
5		20	50	**>95**	75	**>95**	-	-
6	10 μM	1	<5	<5	<5	<5	-	-
7		2	<5	<5	<5	<5	-	-
8		5	<5	<5	<5	25	-	-
9		10	<5	25	30	40	-	-
10		20	25	35	40	55	70	**>95**
11		50	-	-	-	**>95**	-	-

4.8. Synthesis of HSA-3 under optimised conditions

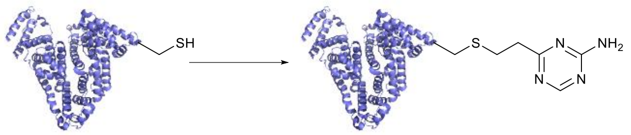

Linker **3** (0.35 µL, 20 mM in DMSO), PBS (8.01 µL, ×1) and DMSO (0.15 µL) were added to a solution of HSA (Cys-34 free form, 1.56 µL, 226 µM in PBS (×1)). After incubation at 37 °C for 1 h, the solution was purified *via* Zeba Spin desalting column (7k MWCO, ThermoFisher, pre-equilibrated with PBS (×1)) to give **HSA-3**.

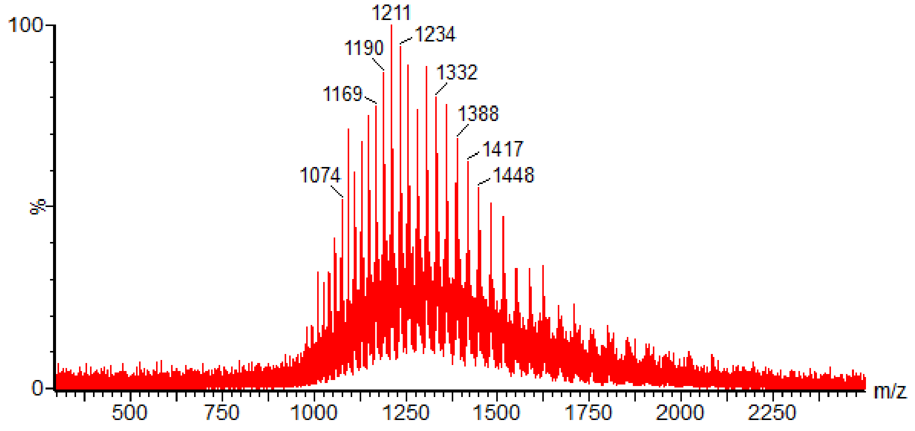

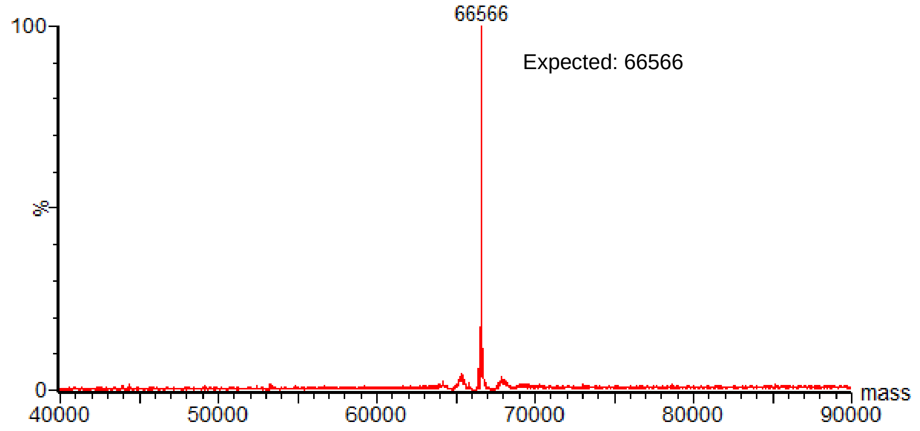

Figure S40. Non-deconvoluted (top) and deconvoluted (bottom) mass spectrum of **HSA-3**. Expected 66567 Da; observed 66566 Da.

4.9. SDS-PAGE analysis of HSA conjugates

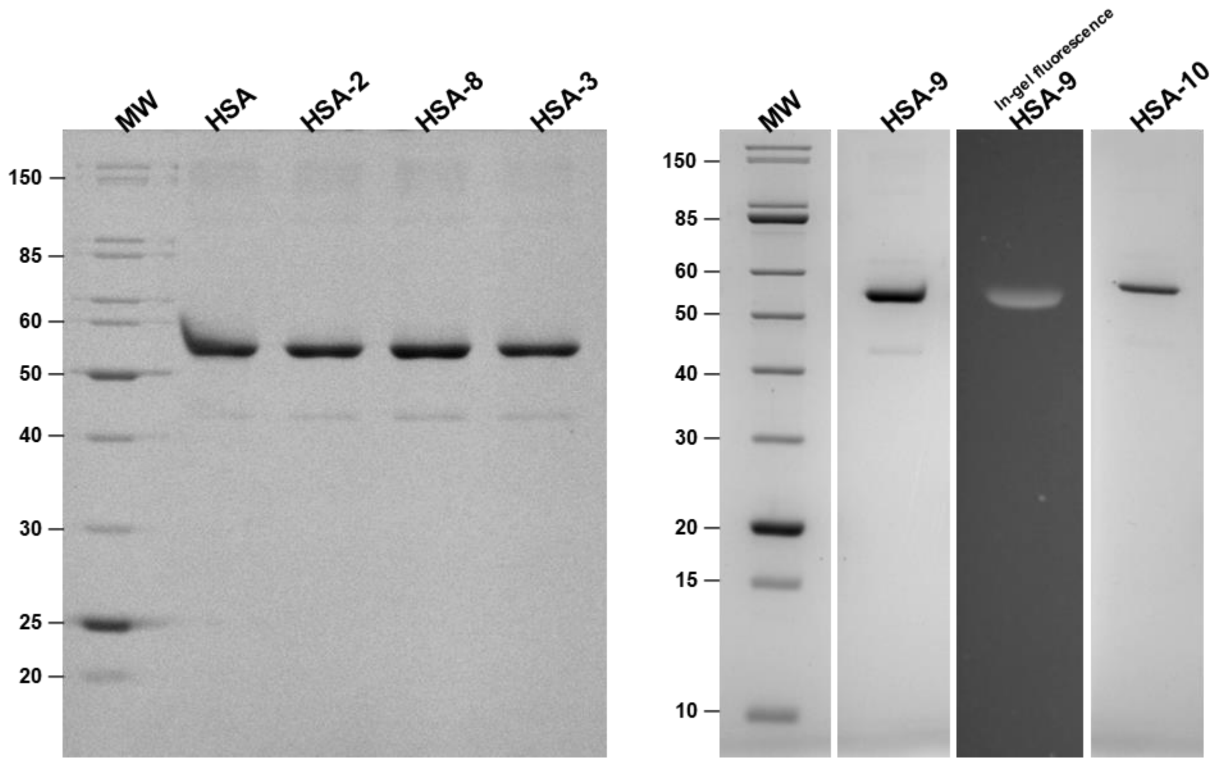

Figure S41. SDS-PAGE analysis with 12% acrylamide gel under non-reducing conditions. SDS-PAGE was analysed by in-gel fluorescence and coomassie brilliant blue staining. MW=molecular weight marker.

5. Modification of cysteine-engineered antibody

5.1. Reduction of cysteine-engineered antibody mAb1

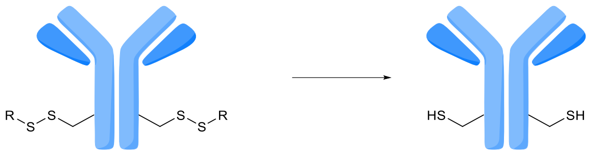

Based on a literature protocol,[5] the engineered cysteines of **mAb1** were uncapped; all subsequent cysteine conjugation reactions were performed on the reduced **mAb1** prepared *via* the following method.

TCEP·HCl (4.5 µL, 50 mM in H_2O) and Tris·HCl (40 µL, pH 8, 50 mM containing 1 mM EDTA) were added to a solution of **mAb1** (40 µL, 70.7 µM in PBS (×1)), and the resulting solution was incubated at 37 °C for 4 h. Removal of excess reagents and buffer exchange to the required solvent was achieved by repeated ultracentrifugation into Tris·HCl (pH 8, 50 mM containing 1 mM EDTA) using an Amicon-Ultra centrifugal filter (10k MWCO, Merck Millipore). To this solution, dehydroascorbic acid (2.26 µL, 25 µM in H_2O) was added, and was left to stand at rt for 2 h, before the solution was purified *via* Zeba Spin desalting column (7k MWCO, ThermoFisher, pre-equilibrated with Tris·HCl (pH 8, 50 mM containing 1 mM EDTA)). Prior to LCMS analysis, samples were deglycosylated and reduced with TCEP·HCl.

[5] Dimasi, N.; Fleming, R.; Zhong, H.; Bezabeh, B.; Kinneer, K.; Christie, R. J.; Fazenbaker, C.; Wu, H.; Gao, C. *Mol. Pharm.* **2017**, *14*, 1501.

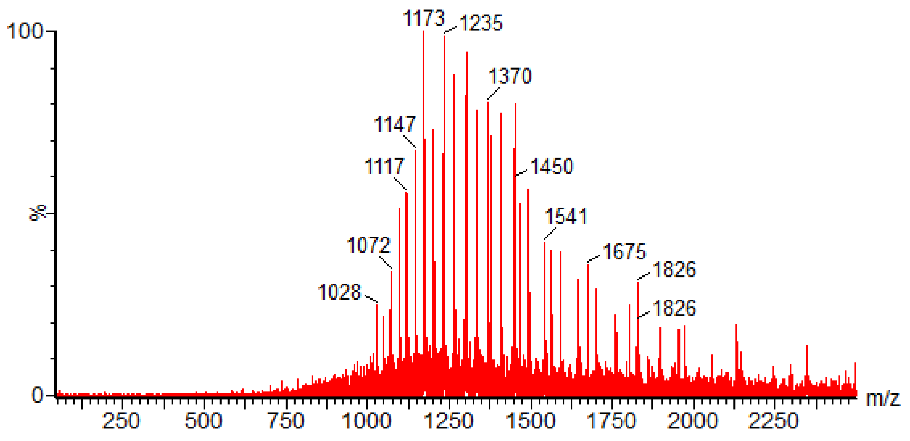

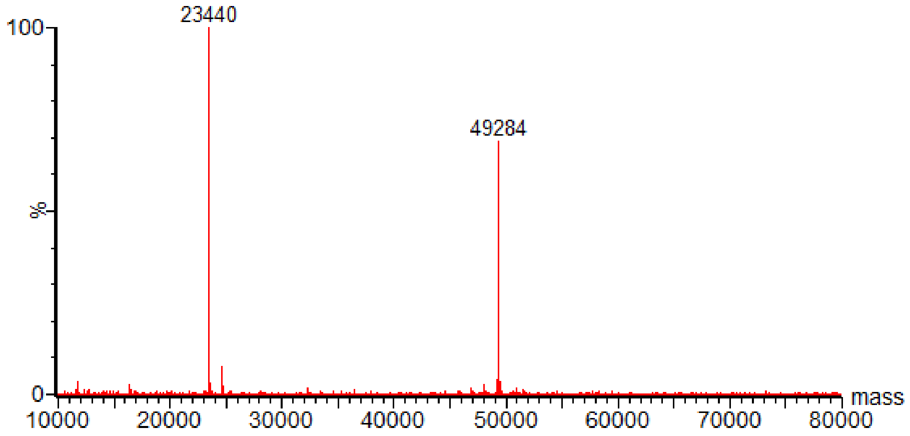

Figure S42. Non-deconvoluted (top) and deconvoluted (bottom) mass spectrum of **mAb$_1$** after deglycosylation with PNGase F and reduction with TCEP·HCl. Observed 23440 Da (LC) and 49284 Da (HC).

5.2. Synthesis of mAb1-2

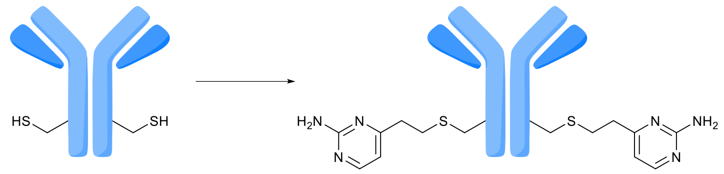

Linker **2** (0.29 µL, 20 mM in DMSO) and DMSO (0.22 µL) were added to a solution of **mAb1** (cysteine uncapped form, 9.5 µL, 31.6 µM in Tris·HCl (pH 8, 50 mM containing 1 mM EDTA)), and the resulting solution was incubated at 37 °C for 4 h. The solution was purified *via* Zeba Spin desalting column (7k MWCO, ThermoFisher, pre-equilibrated with Tris·HCl (pH 8, 50 mM containing 1 mM EDTA)) to give **mAb1-2**. Prior to LCMS analysis, samples were deglycosylated with PNGase F and reduced with TCEP·HCl.

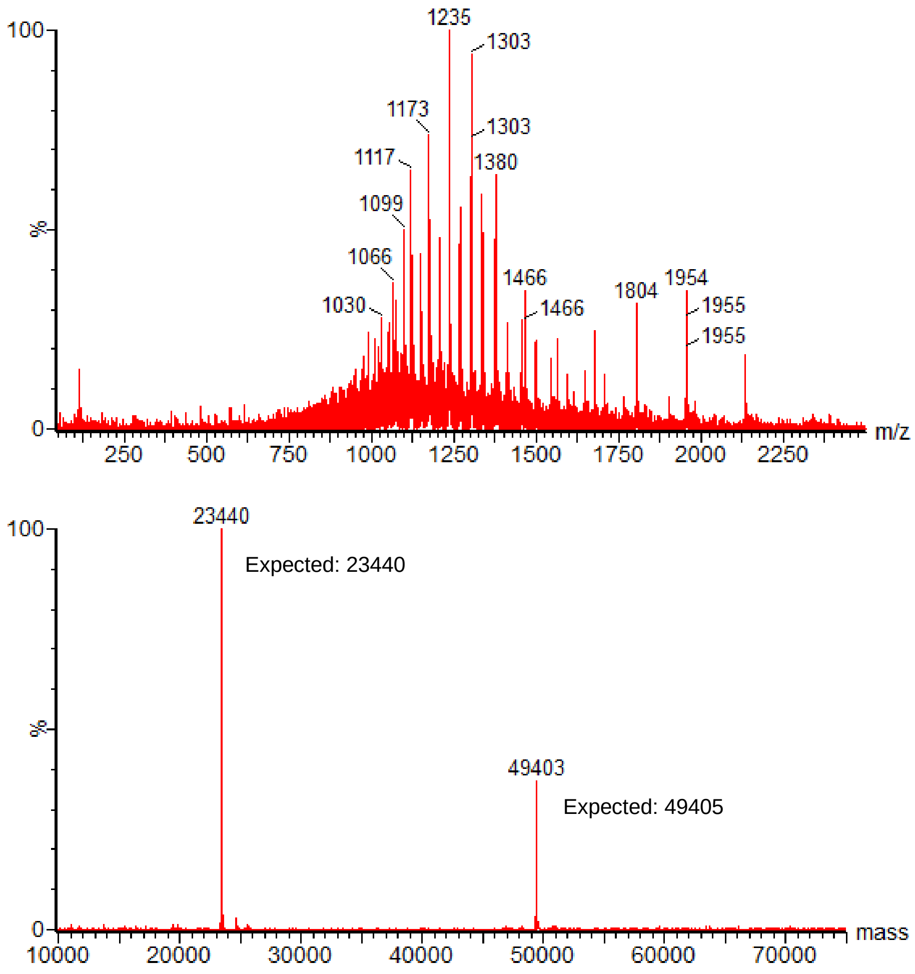

Figure S43. Non-deconvoluted (top) and deconvoluted (bottom) mass spectrum of **mAb1-2**. Expected 23440 Da (LC) and 49405 Da (HC); observed 23440 Da (LC) and 49403 Da (HC). Prior to LCMS analysis, samples were deglycosylated with PNGase F and reduced with TCEP·HCl.

5.3. Synthesis of mAb1-8

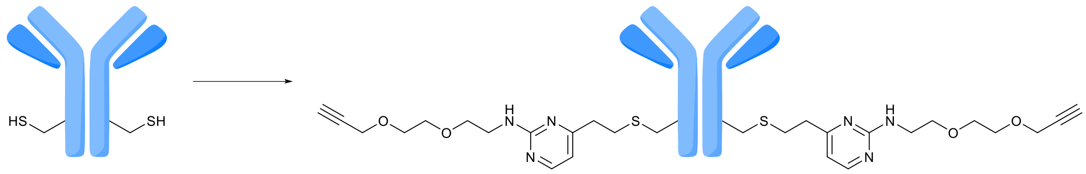

Linker **8** (0.50 µL, 30 mM in DMSO) was added to a solution of **mAb1** (cysteine uncapped form, 9.5 µL, 31.6 µM in Tris·HCl (pH 8, 50 mM containing 1 mM EDTA)), and the resulting solution was incubated at 37 °C for 4 h. The solution was purified *via* Zeba Spin desalting column (7k MWCO, ThermoFisher, pre-equilibrated with Tris·HCl (pH 8, 50 mM)) to give **mAb1-8**. Prior to LCMS analysis, samples were deglycosylated with PNGase F and reduced with TCEP·HCl.

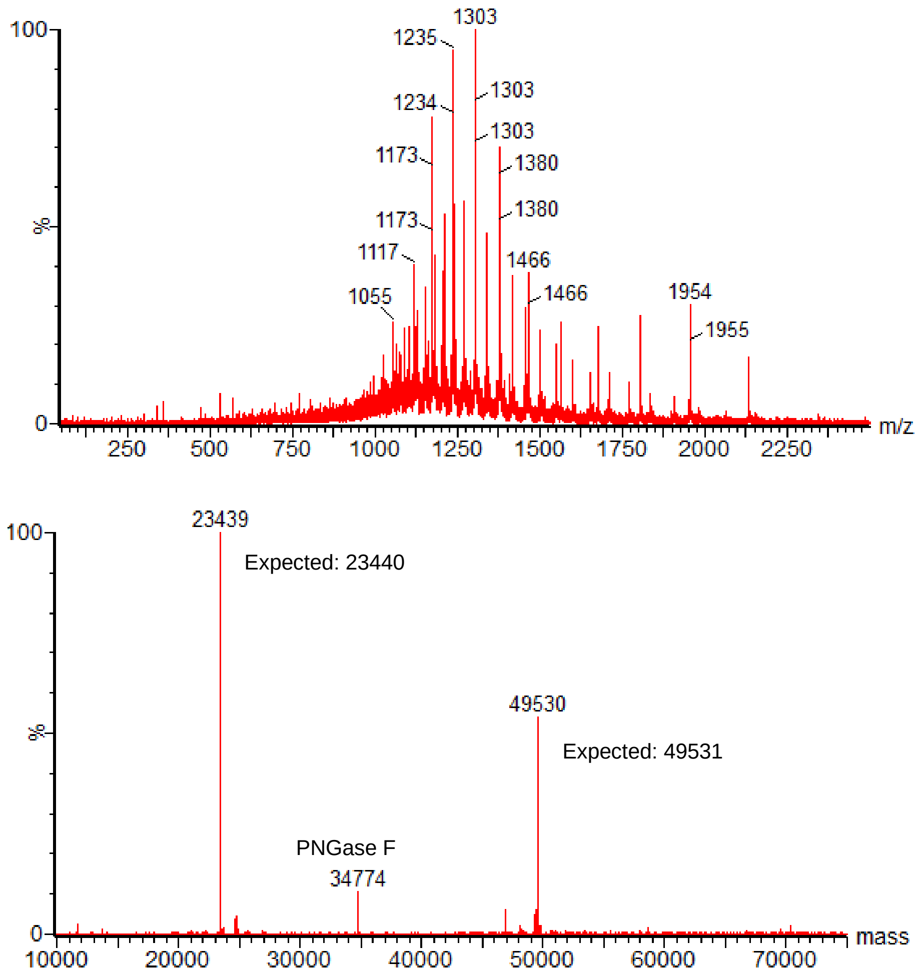

Figure S44. Non-deconvoluted (top) and deconvoluted (bottom) mass spectrum of **mAb1-8**. Expected 23440 Da (LC) and 49531 Da (HC); observed 23439 Da (LC) and 49530 Da (HC). Prior to LCMS analysis, samples were deglycosylated with PNGase F and reduced with TCEP·HCl. The signal at 34774 Da corresponds to PNGase F.

5.4. Synthesis of mAb1-8-12

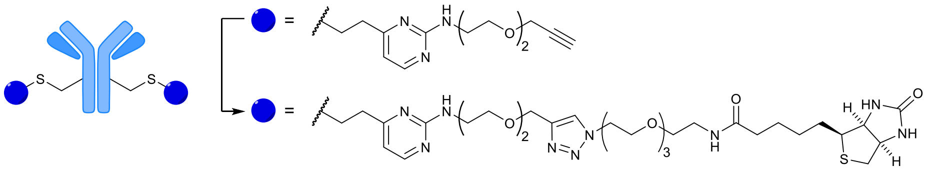

Aqueous THPTA (0.29 µL 20 mM), aqueous $CuSO_4 \cdot 5H_2O$ (0.23 µL, 5 mM), aqueous sodium ascorbate (0.34 µL, 50 mM), DMSO (1 µL) and azido-biotin **12** (0.57 µL, 20 mM in DMSO) were added sequentially to a solution of **mAb1-8** (10 µL, 11.4 µM in PBS (×1)). The resulting solution was incubated at 37 °C for 15 h before purification *via* Zeba Spin desalting column (7k MWCO, ThermoFisher, pre-equilibrated with PBS (×1)) to give **mAb1-8-12** Prior to LCMS analysis, samples were deglycosylated with PNGase F and reduced with TCEP·HCl.

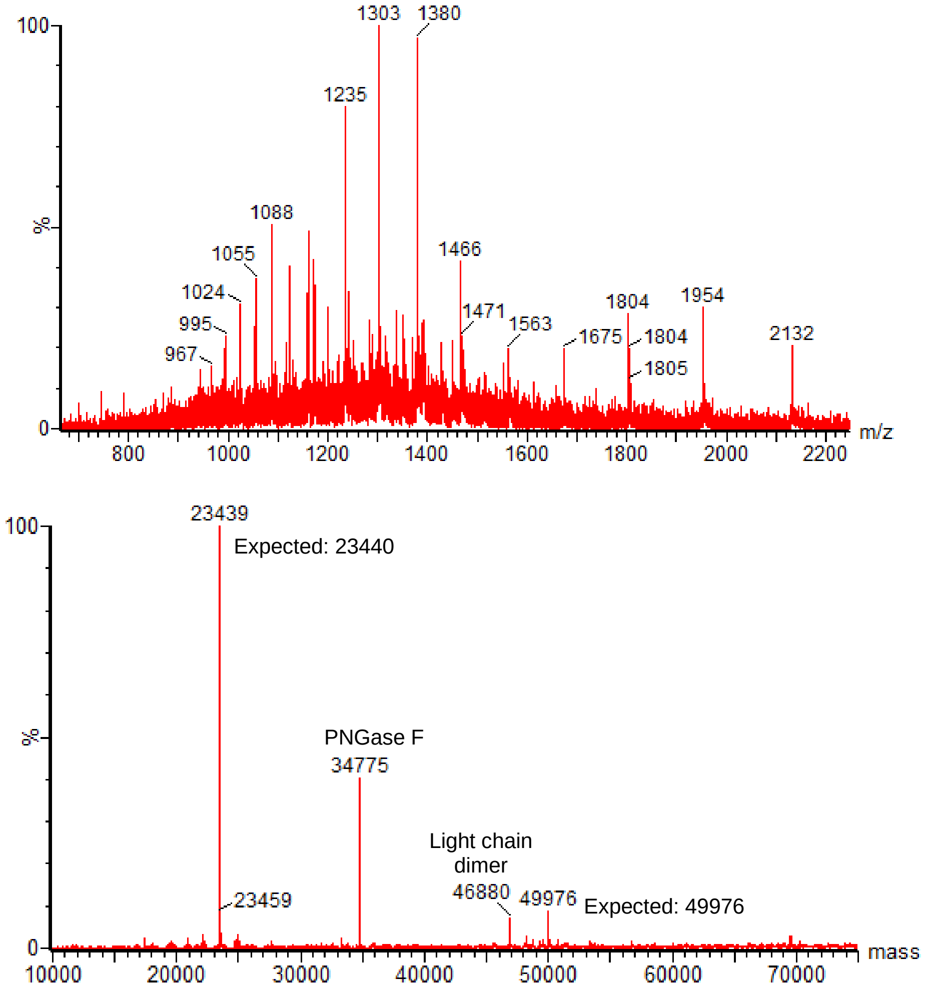

Figure S45. Non-deconvoluted (top) and deconvoluted (bottom) mass spectrum of **mAb1-8-12**. Expected 23440 Da (LC) and 49976 Da (HC); observed 23440 Da (LC) and 49976 Da (HC). Prior to LCMS analysis, samples were deglycosylated with PNGase F and reduced with TCEP·HCl. The signals at 34774 Da and 46880 Da corresponds to PNGase F and light chain dimer, respectively.

5.5. Synthesis of mAb$_1$-8-13

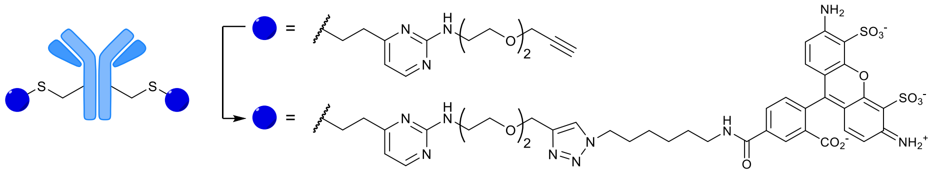

Aqueous THPTA (0.29 µL, 80 mM), aqueous $CuSO_4·5H_2O$ (0.23 µL, 20 mM), aqueous sodium ascorbate (0.34 µL, 200 mM), DMSO (1 µL) and Alexa Fluor 488 azide **13** (0.57 µL, 20 mM in DMSO, purchased from Invitrogen) were added sequentially to a solution of **mAb$_1$-8** (10 µL, 11.4 µM in PBS (×1)). The resulting solution was incubated at 37 °C for 6 h before purification *via* Zeba Spin desalting column (7k MWCO, ThermoFisher, pre-equilibrated with PBS (×1)). The resulting conjugate was analysed by UV-Vis spectroscopy,[6] which revealed conjugate **mAb$_1$-8-13** to have a fluorophore-to-antibody ratio of 1.9.

5.6. Synthesis of mAb$_1$-8-14

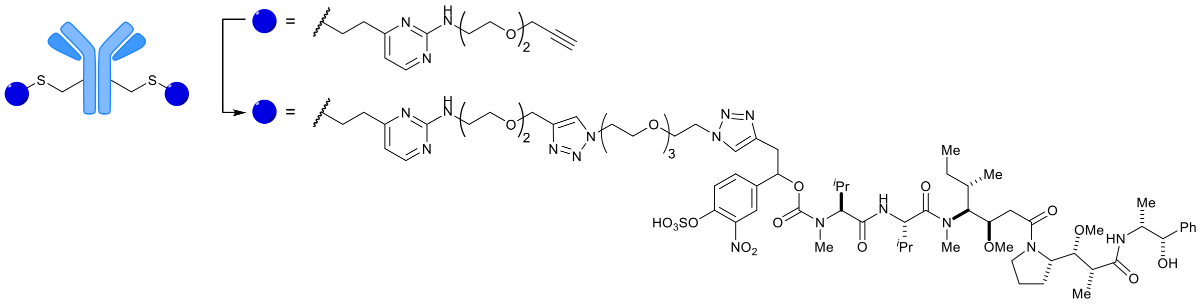

Aqueous THPTA (2.5 µL, 80 mM), aqueous $CuSO_4·5H_2O$ (3.5 µL, 20 mM), aqueous sodium ascorbate (1.7 µL, 200 mM) and azido-MMAE **14** (1.7 µL, 20 mM in DMSO) were added sequentially to a solution of **mAb$_1$-8** (25 µL, 27.3 µM in PBS (×1)). The resulting solution was incubated at 37 °C for 6 h before purification *via* Zeba Spin desalting column (two rounds of purification, 7k MWCO, ThermoFisher, pre-equilibrated with PBS (×1)) and further ultracentrifgation to PBS (×1) using Amicon-Ultra centrifugal filter (10k MWCO, Merck Millipore) to give **mAb$_1$-8-14**. Prior to LCMS analysis, samples were deglycosylated with PNGase F and reduced with TCEP·HCl.

[6] Refer to General Experimental for procedure.

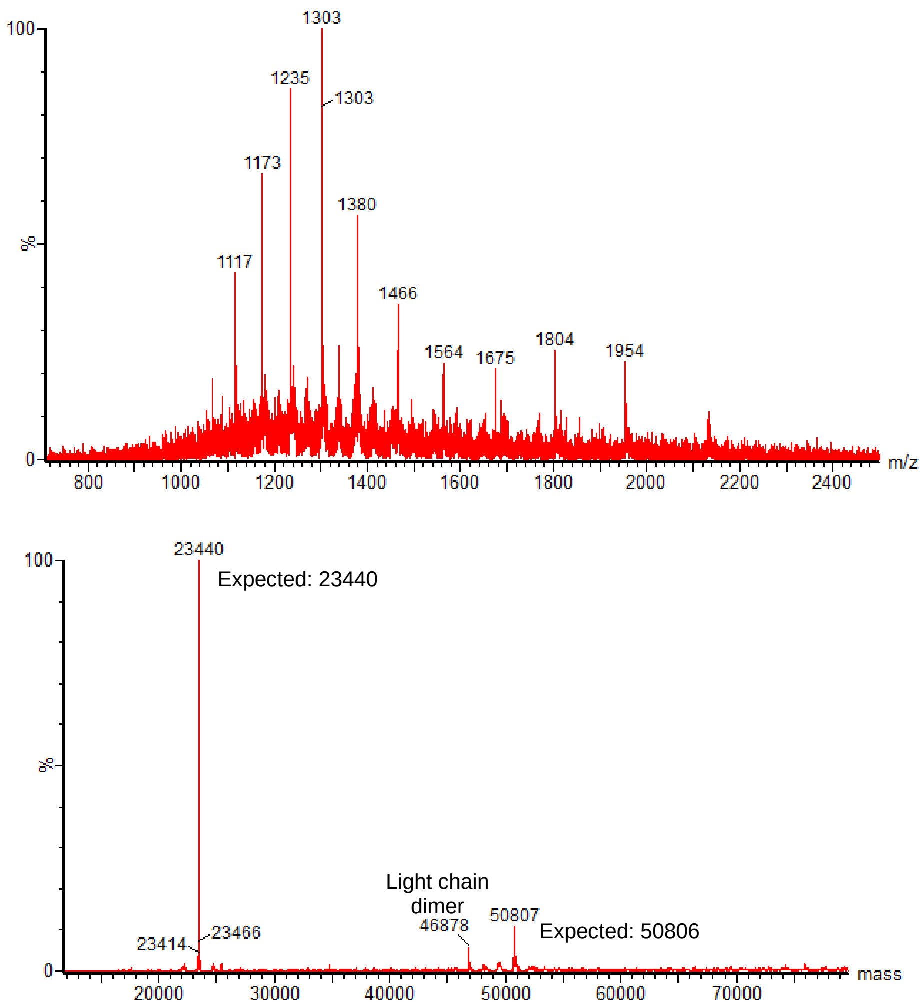

Figure S46. Non-deconvoluted (top) and deconvoluted (bottom) mass spectrum of **mAb₁-8-14**. Expected 23440 Da (LC; no modification) and 50806 Da (HC); observed 23440 Da (LC) and 50707 Da (HC). Prior to LCMS analysis, samples were deglycosylated with PNGase F and reduced with TCEP·HCl. The signal at 46878 Da corresponds to light chain dimer.

5.7. SDS-PAGE analysis of mAb_1 conjugates

SDS-PAGE analysis of **mAb_1** and mAb_1 conjugates under non-reducing (NR) and reducing (R) conditions. Analysis of **mAb_1-8**, **mAb_1-12** and **mAb_1-13** reveals that conjugates are found predominantly as the "full antibody" forms (~146 kDa), with all native interchain disulfide bonds present. In-gel fluorescence of Alexa Fluor 488 conjugate **mAb_1-8-13** under reducing conditions revealed the linker modification to be on the heavy chain (~51 kDa), consistent with mass spectrometry analysis; fluorescence was not observed for the light chain (~23 kDa).

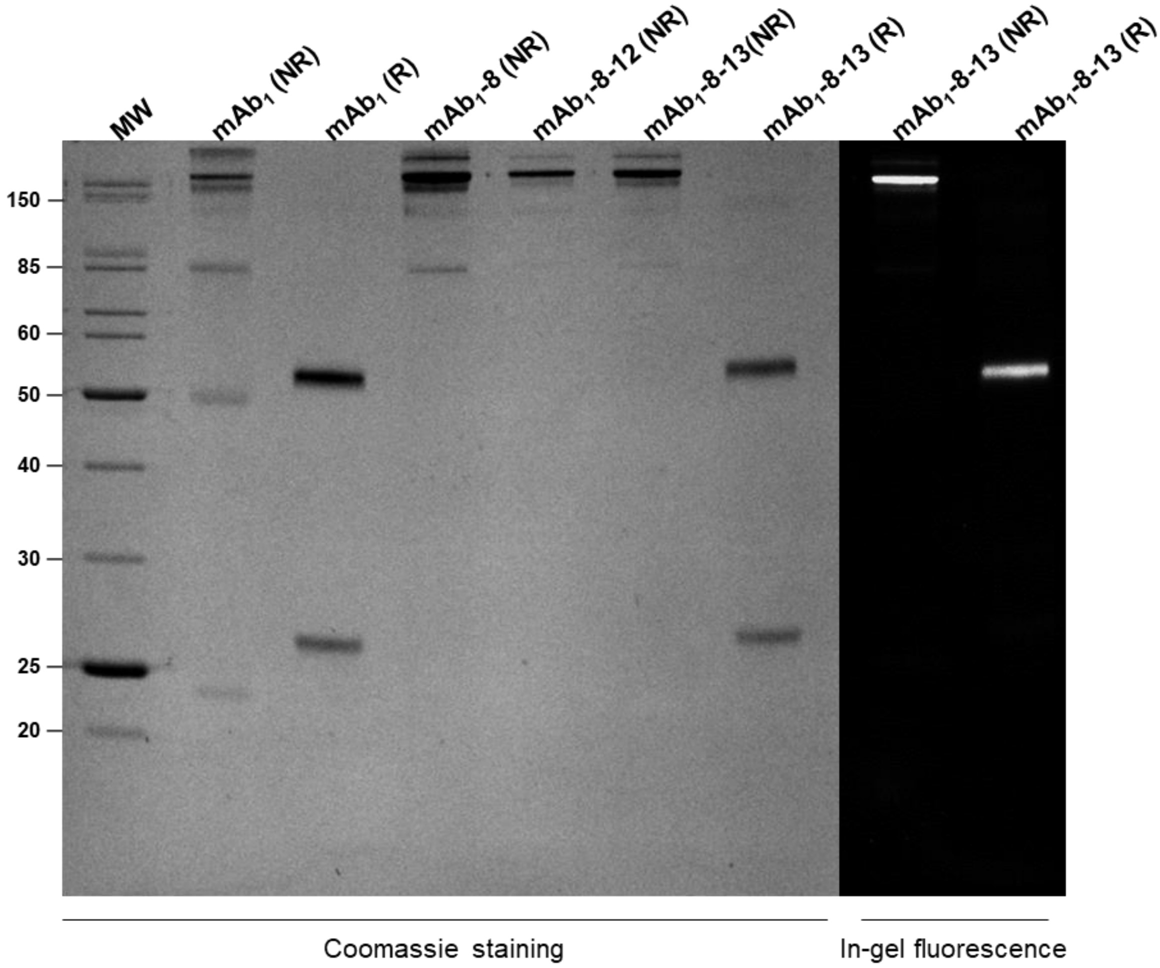

Figure S47. SDS-PAGE analysis with 12% acrylamide gel under non-reducing (NR) and reducing (R) conditions. SDS-PAGE was analysed by in-gel fluorescence and coomassie brilliant blue staining. MW=molecular weight marker.

6. Preparation of fluorophore-modified trastuzumab and plasma stability

6.1. Mass spectra of native trastuzumab

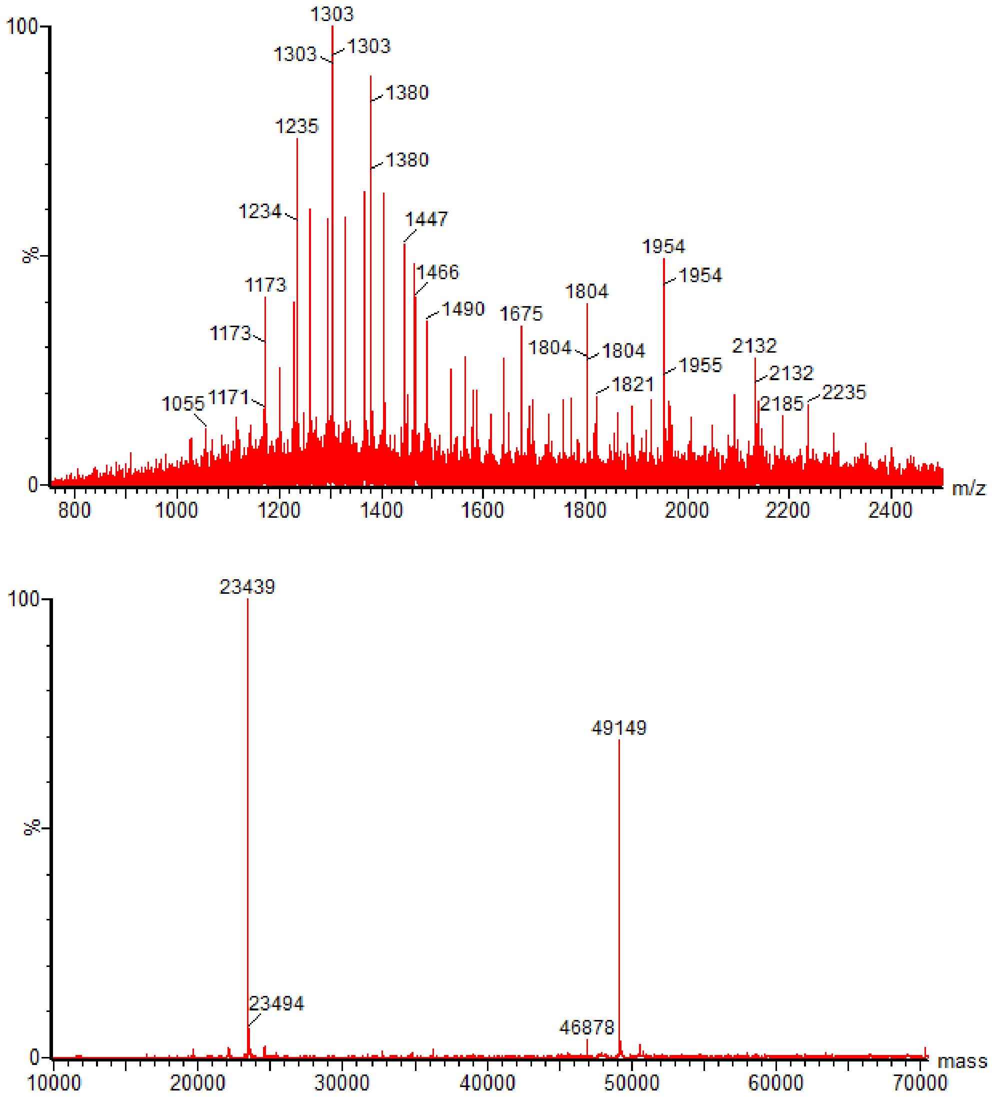

Figure S48. Non-deconvoluted (top) and deconvoluted (bottom) mass spectrum of native trastuzumab **mAb**$_2$. Prior to LCMS analysis, samples were deglycosylated with PNGase F and reduced with TCEP·HCl.

6.2. Synthesis of mAb$_2$-8

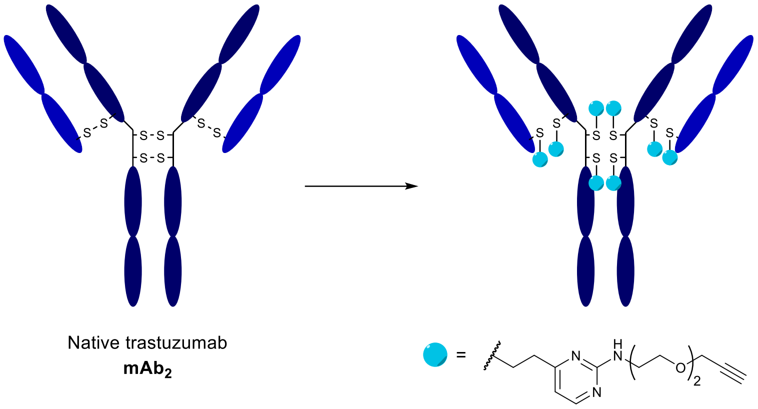

Tris-buffered saline (25 mM pH 8 aqueous Tris·HCl, 25 mM NaCl, 0.5 mM EDTA; 9.0 µL) and aqueous TCEP·HCl (0.4 µL, 5 mM) were added to a solution of native trastuzumab **mAb$_2$** (3.0 µL, 45 µM), and the resulting solution was incubated at 37 °C for 1 h. To this solution, DMSO (0.27 µL) and **8** (1.07 µL, 12.5 mM in DMSO) were added, and the resultant solution was incubated at 37 °C for 6 h, before purification *via* Zeba Spin desalting column (7k MWCO, ThermoFisher, pre-equilibrated with PBS (×1)). Finally, buffer exchange to PBS (×1) was achieved by repeated ultracentrifugation using an Amicon-Ultra centrifugal filter (10k MWCO, Merck Millipore) to give **mAb$_2$-8**. Prior to LCMS analysis, samples were deglycosylated with PNGase F and treated with TCEP·HCl.

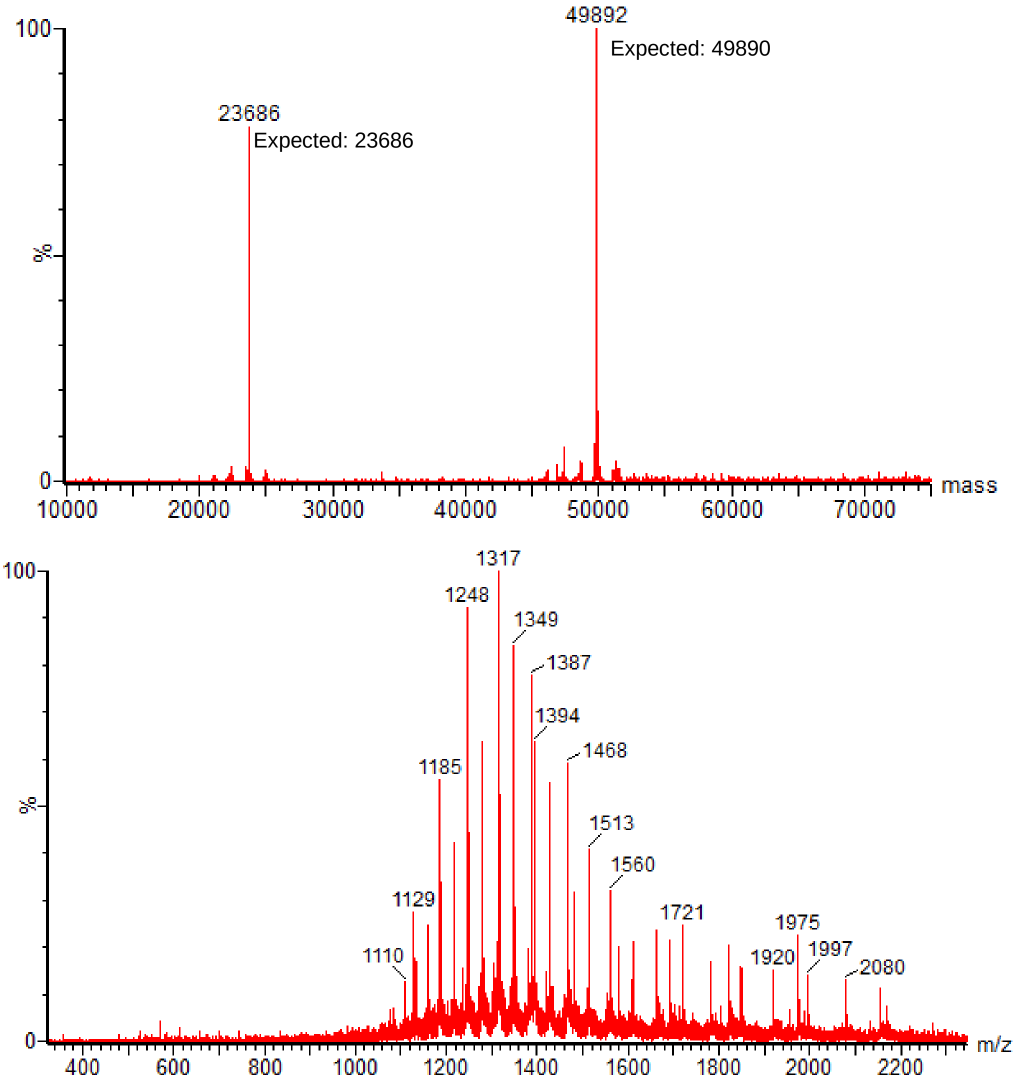

Figure S49. Non-deconvoluted (top) and deconvoluted (bottom) mass spectrum of native trastuzumab **mAb_2** modified with vinylpyrimidine linker **8**. The light chain and heavy chain are modified with one and three linkers, respectively, to give a linker-to-antibody ratio of 8. Expected 23686 Da (LC) and 49890 Da (HC). Found 23686 Da (LC) and 49892 Da (HC). Prior to LCMS analysis, samples were deglycosylated with PNGase F and treated with TCEP·HCl.

6.3. Synthesis of mAb2-8-13

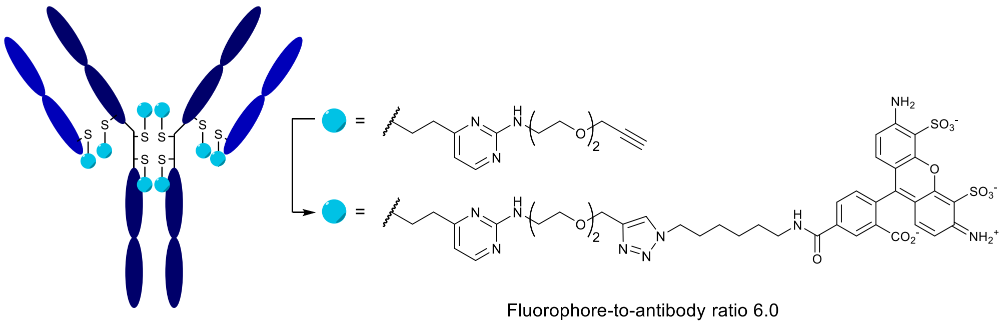

Aqueous THPTA (1.62 µL, 80 mM), aqueous $CuSO_4{\cdot}5H_2O$ (1.08 µL, 20 mM), aqueous sodium ascorbate (1.08 µL, 200 mM) and Alexa Fluor 488 azide **13** (2.16 µL, 20 mM in DMSO, purchased from Invitrogen) were added sequentially to a solution of **mAb2-8** (30 µL, 12.6 µM in PBS (×1)). The resulting solution was incubated at 37 °C for 15 h before purification *via* Zeba Spin desalting column (7k MWCO, ThermoFisher, pre-equilibrated with PBS (×1)). The resulting conjugate was analysed by UV-Vis spectroscopy, which revealed conjugate **mAb2-8-13** to have a fluorophore-to-antibody ratio of 6.0.

6.4. Synthesis of mAb2-15

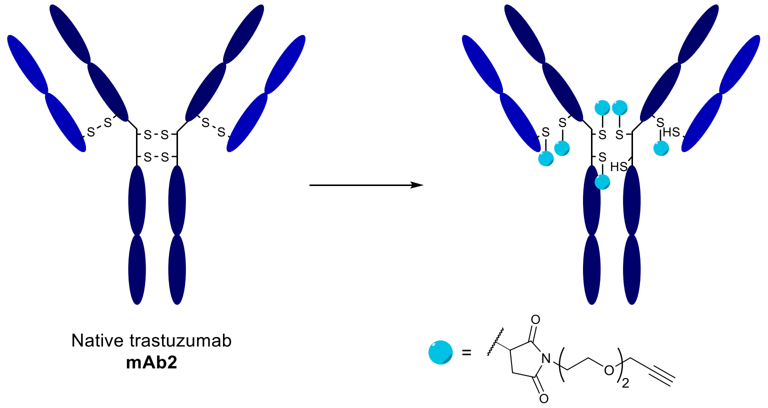

This bioconjugate was prepared based on a literature procedure.[7]

Aqueous TCEP·HCl (1.2 µL, 5 mM) was added to a solution of native trastuzumab **mAb2** (20 µL, 29.7 µM in PBS (×1)) and the solution was incubated at 37 °C for 1 h. To this solution, DMSO (1.7 µL) and maleimide **15** (0.6 µL, 20 mM in DMSO) were added. The resulting solution was left to stand at rt for 1.5 h, before purification *via* Zeba Spin desalting column (7k

[7] Li, W.; Veale, K. H.; Qiu, Q.; Sinkevicius, K. W.; Maloney, E. K.; Costoplus, J. A.; Lau, J.; Evans, H. L.; Setiady, Y.; Ab, O.; et al. *ACS Med. Chem. Lett.* **2019**, *10*, 1386.

MWCO, ThermoFisher, pre-equilibrated with PBS (×1)) to give **mAb$_2$-15**. Prior to LCMS analysis, samples were deglycosylated with PNGase F and treated with TCEP·HCl.

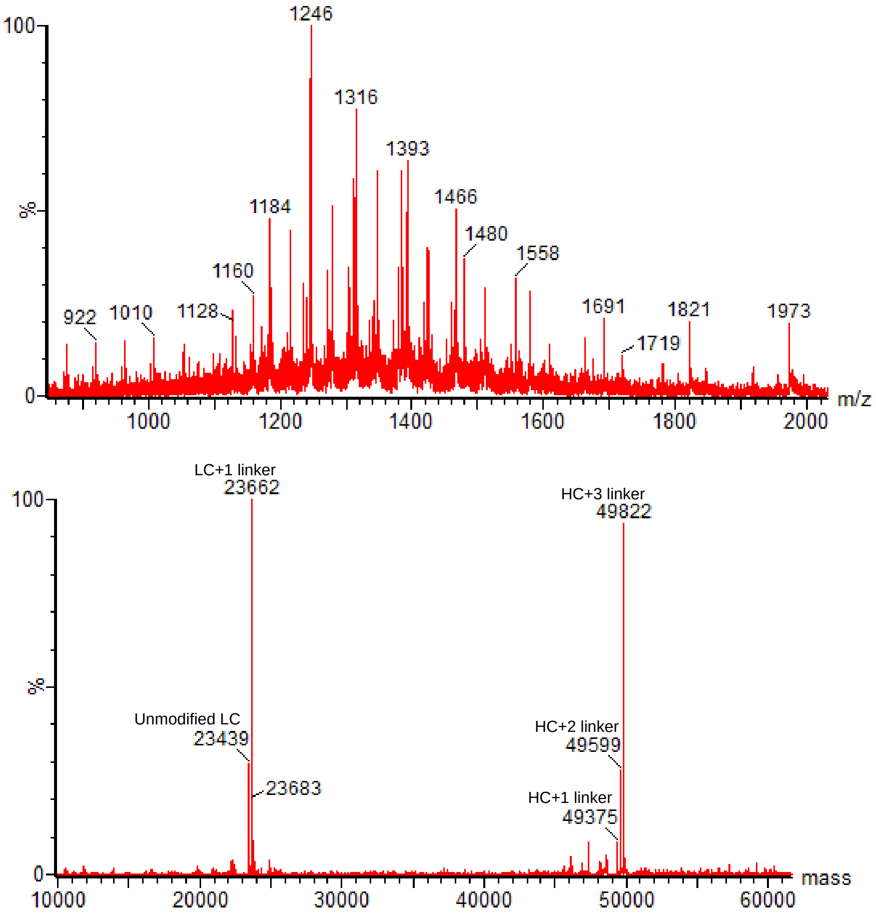

Figure S50. Non-deconvoluted (top) and deconvoluted (bottom) mass spectrum of native trastuzumab **mAb$_2$** with maleimide linker **15**. The reaction gave a mixture of reacted ad unreacted cysteine residues. light chain and heavy chain are modified with one and three linkers, respectively, to give a linker-to-antibody ratio of 8. The observed masses correspond to the following species: 23439 Da (unmodified LC; expected 23439 Da); 23662 Da (LC modified with one linker; expected 23662 Da); 49375 Da (HC modified with one linker; expected 49372 Da); 49599 Da (HC modified with two linkers; expected 49595 Da); 49822 Da (HC modified with three linkers; expected 49818 Da). Prior to LCMS analysis, samples were deglycosylated with PNGase F and treated with TCEP·HCl.

6.5. Synthesis of mAb2-15-13

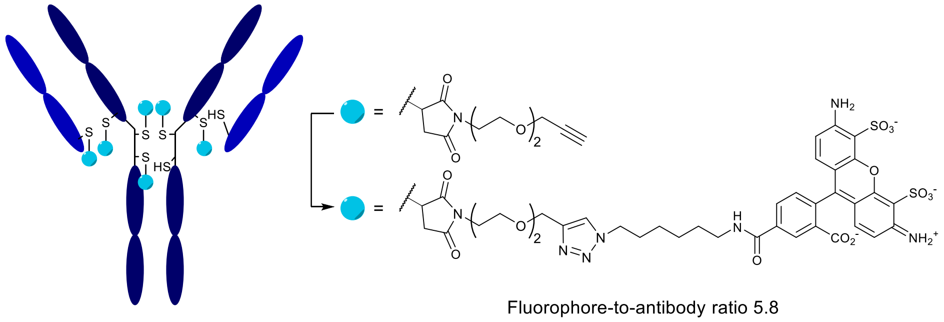

Fluorophore-to-antibody ratio 5.8

Aqueous THPTA (1.62 µL, 80 mM), aqueous $CuSO_4{\cdot}5H_2O$ (1.08 µL, 20 mM), aqueous sodium ascorbate (1.08 µL, 200 mM) and Alexa Fluor 488 azide **13** (2.16 µL, 20 mM in DMSO, purchased from Invitrogen) were added sequentially to a solution of **mAb2-15** (30 µL, 12.6 µM in PBS (×1)). The resulting solution was incubated at 37 °C for 15 h before purification *via* Zeba Spin desalting column (7k MWCO, ThermoFisher, pre-equilibrated with PBS (×1)). The resulting conjugate was analysed by UV-Vis spectroscopy, which revealed conjugate **mAb2-15-13** to have a fluorophore-to-antibody ratio of 5.8.

6.6. Plasma stability studies

Solutions containing 0.5 μM of bioconjugate **mAb$_2$-8-13** (fluorophore-to-antibody ratio = 6.0) or **mAb$_2$-15-13** (fluorophore-to-antibody ratio = 5.8) were prepared in PBS (×1) containing 10% human plasma. These solutions were incubated at 37 °C. On day 0, 2, 4, 6 and 8, aliquots were taken and frozen at -80 °C until analysis. These samples were analysed by sodium dodecyl sulfate–polyacrylamide gel electrophoresis (SDS-PAGE),[8] where 1.25 pmol of antibody was loaded in each lane (Figure S51).

An analogous stability study was also conducted using human serum instead of human plasma (Figure S52).

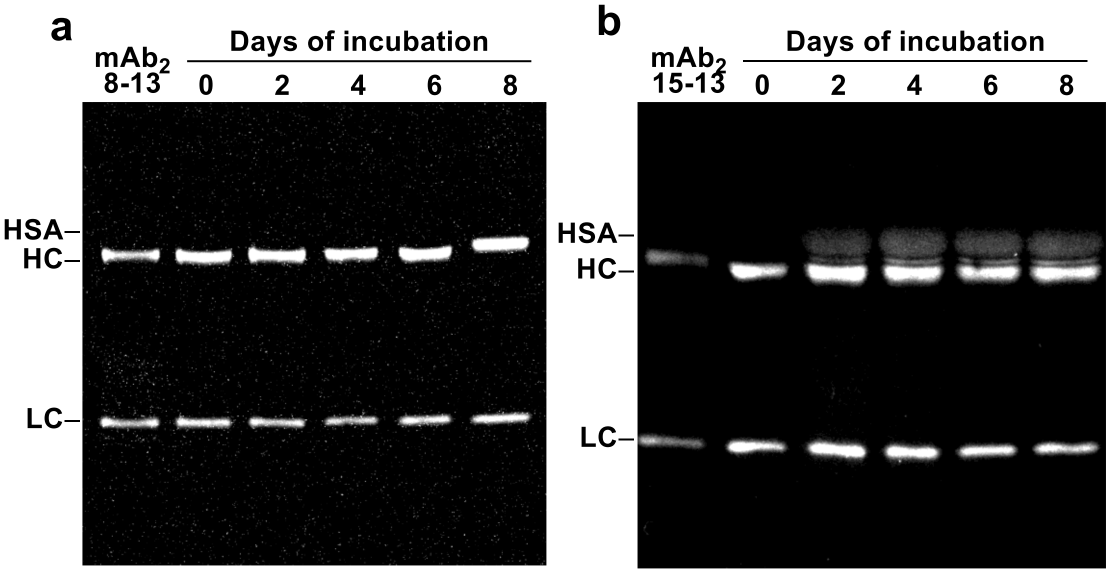

Figure S51. Plasma stability analysis for **a) mAb$_2$-8-13** and **b) mAb$_2$-15-13** In-gel fluorescence analysis displays no transfer of fluorescence for **mAb$_2$-8-13**, whereas fluorescence transfer to serum proteins was observed for **mAb$_2$-15-13**. All lanes were prepared under reducing conditions.

[8] For an example of stability determination using SDS-PAGE, see Kolodych, S.; Koniev, O.; Baatarkhuu, Z.; Bonnefoy, J. Y.; Debaene, F.; Cianférani, S.; Van Dorsselaer, A.; Wagner, A. *Bioconjug. Chem.* **2015**, *26*, 197.

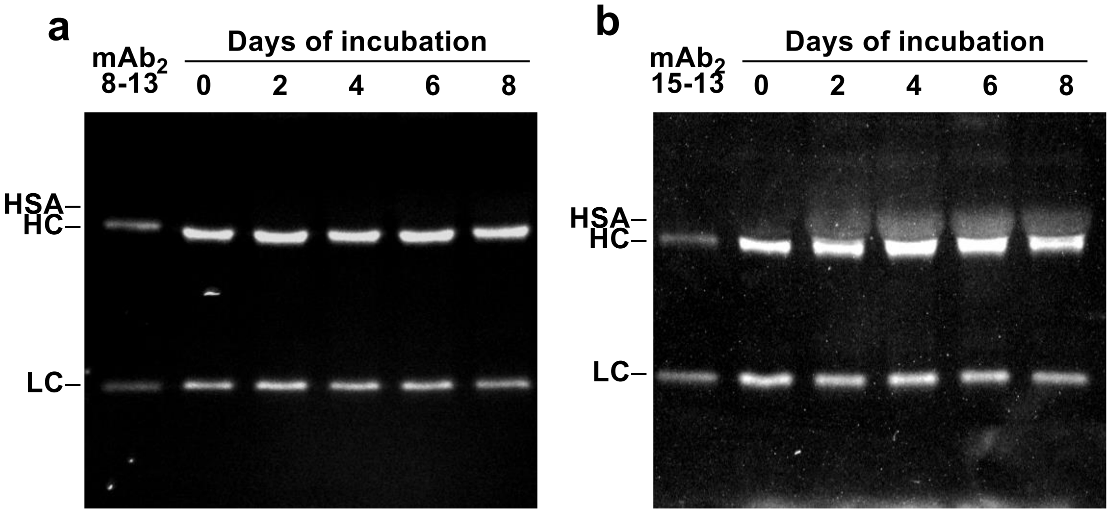

Figure S52. Serum stability analysis for **a) mAb$_2$-8-13** and **b) mAb$_2$-15-13** In-gel fluorescence analysis displays no transfer of fluorescence for **mAb$_2$-8-13**, whereas fluorescence transfer to serum proteins was observed for **mAb$_2$-15-13**. All lanes were prepared under reducing conditions.

7. *In vitro* cytotoxicity studies

SKBR3 cells were obtained from the American Type Culture Collection (ATCC) and MCF7 cells were obtained from the European Collection of Authenticated Cell Cultures (ECACC). SKBR3 cells were maintained in high glucose McCoy's 5A medium, supplemented with 10% heat-inactivated foetal-bovine serum (FBS), 50 U/mL penicillin and 50 µg/mL streptomycin. MCF7 cells were maintained in Dulbecco's Modified Eagle Medium (DMEM) supplemented with 10% FBS, 2 mM L- glutamine, 50 U/mL penicillin and 50 µg/mL streptomycin. All cell lines were incubated at 37 °C with 5% CO2.

Cells were seeded in 96-well plates for 24 h at 37 °C with 5% CO2. SKBR3 cells were seeded at 20,000 cells/well and MCF7 cells seeded at 7,500 cells/well. Serial dilutions of **mAb$_1$-8-14** was added to the cells in complete growth medium and incubated at 37 °C with 5% CO2 for 96 h. Cell viability was measured using CellTiter-Glo viability assay (Promega) according to the manufacturer's instructions. Cell viability was plotted as a percentage of untreated cells. Each measurement was taken in triplicate and three independent repeats were performed.

8. Cell lysate labelling

8.1. Preparation of cell lysate

MCF7 cells (2 × T175 flasks) were trypsinised and washed with PBS (3 × 10 mL). Cell pellets were reconstituted in RIPA buffer (Thermo, 89900) supplemented with protease inhibitors (Roche). Cells were then lysed by sonication (Diagenode, 2 × 30 s cycles) and centrifuged at 21,000 ×g at 4 °C for 15 min. The protein-containing supernatant was removed, and protein concentration (~5 mg/mL) measured using a Direct Detect Spectrometer (Millipore). Samples were diluted to 1 mg/mL with PBS, aliquoted, flash frozen and stored at -20 °C until use.

8.2. Labelling of cell lysate

A ×20 stock solution of vinylpyrimidine-alkyne **8** (2.5 µL; 20µL, 200µL, 1 mM, 2 mM, 4 mM, or 8mM in DMSO) or neat DMSO (2.5 µL; control reaction) was added to MCF7 cell lysate (50 µL, 1 mg/mL), to give final linker concentrations of 1 µM, 10 µM, 50 µM, 100 µM, 200 µM, 400 µM, and 0 µM, respectively. The resulting solutions were left to stand at rt for 2 h, before purification *via* Zeba Spin desalting column (7k MWCO, ThermoFisher, pre-equilibrated with PBS (×1)). To each solution, THPTA (500 mM in H_2O, 1 µL, 500 pmol), $CuSO_4 \cdot 5H_2O$ (50 mM in H_2O, 1 µL, 50 pmol), sodium ascorbate (1 M in H_2O, 1 µL, 1 µmol) and Alexa Fluor 488 azide **13** (5 mM in DMSO, 2 µL, 10 pmol) were added sequentially, and the resulting mixture was left to stand at rt for 1.5 h, before purification *via* Zeba Spin desalting column (7k MWCO, ThermoFisher, pre-equilibrated with PBS (×1)). Material corresponding to a 7.5 µL aliquot of this resulting solution was used for SDS-PAGE analysis. Samples were prepared under reducing conditions and run using methods described in the general experimental.

For cysteine blockade studies, MCF7 cell lysate (50 µL, 1 mg/mL) was pre-incubated with iodoacetamide (20 mM) at rt for 1 h, prior to the addition of vinylpyrimidine-alkyne **8**.

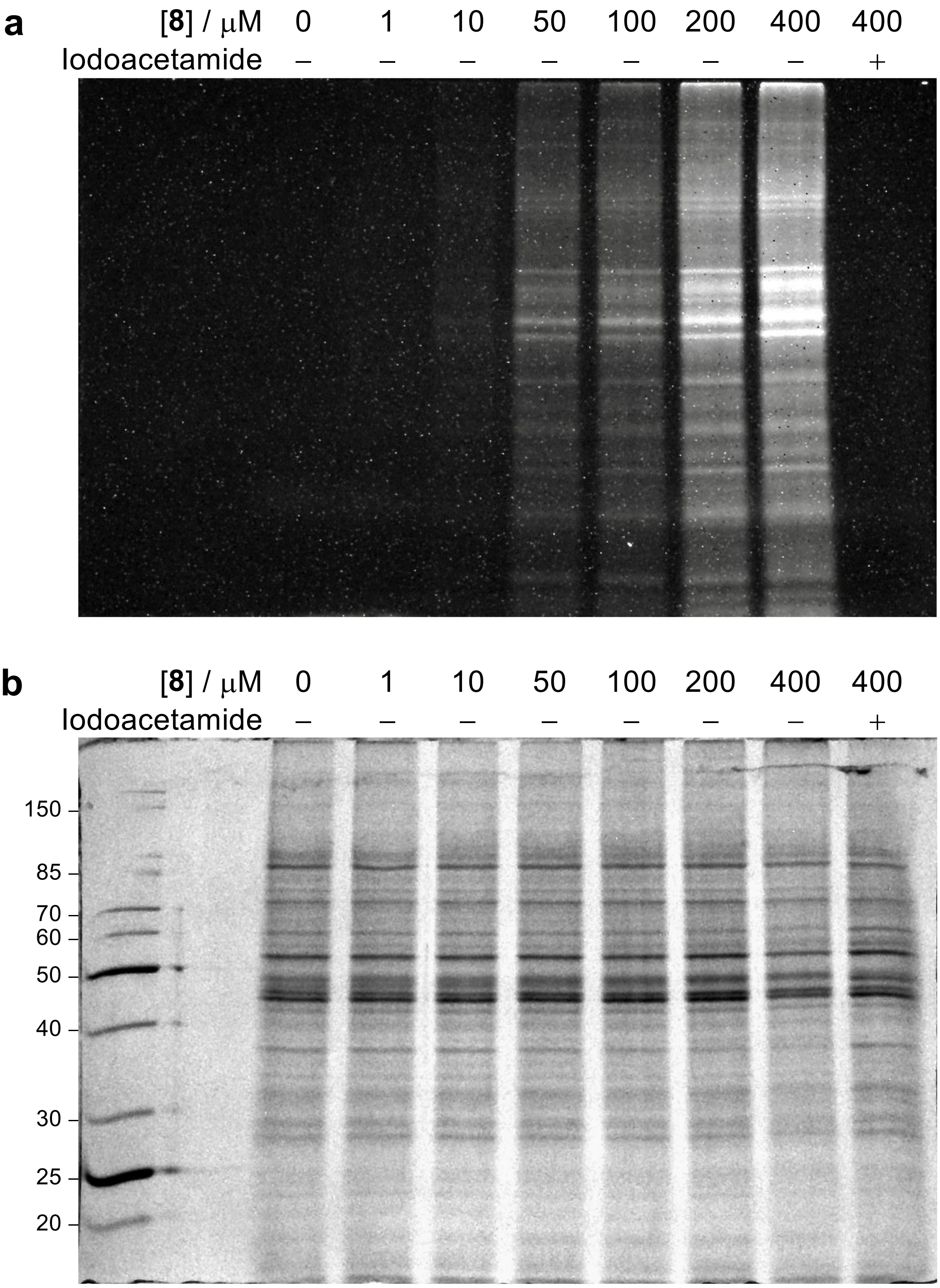

Figure S53. SDS-PAGE analysis of MCF7 cell lysate labelling studies. Cell lysates were first labelled with varying concentrations of probe **8**, and further modified with Alexa Fluor 488 azide **13** *via* a CuAAC reaction. For cysteine blockade studies, cell lysates were first pre-incubated with iodoacetamide. **a)** In-gel fluorescence of cell lysates. **b)** Coomassie staining of cell lysates. All lanes were prepared under reducing conditions. Molecular weight ladder in kDa.

9. Chemical synthesis

4-Vinylpyridin-2-amine 1

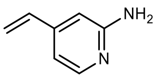

A stirred mixture of 4-bromopyridin-2-amine (250 mg, 1.45 mmol), potassium vinyltrifluoroborate (223 mg, 1.66 mmol), Pd(dppf)Cl_2·CH_2Cl_2 (59 mg, 0.072 mmol) and K_2CO_3 (240 mg, 1.74 mmol) in THF/H_2O (10:1, 5.5 mL) was heated at 70 °C for 14 h. The reaction mixture was filtered through Celite® (eluent EtOAc) and the resultant filtrate was concentrated *in vacuo*. The resulting residue was purified by flash column chromatography (MeOH/CH_2Cl_2, 1:19 with 0.5% Et_3N) to give vinylpyridine **1** (115 mg, 0.957 mmol, 66%) as a dark brown solid.

R_f (SiO_2; MeOH/CH_2Cl_2 1:10 with 1% Et_3N) 0.60; **ν_{max}** (neat/cm^{-1}) 3434, 3291, 3160, 1620, 1598, 1541; **^{1}H NMR** (CD_3OD, 400 MHz) δ 7.82 (1H, d, *J* = 5.6 Hz), 6.70 (1H, dd, *J* = 5.6, 1.6 Hz), 6.65 – 6.54 (2H, m), 5.93 (1H, dd, *J* = 17.6, 0.8 Hz), 5.42 (1H, d, *J* = 10.8 Hz); **^{13}C NMR** (CD_3OD, 101 MHz) δ 161.3, 148.5, 147.9, 136.5, 118.5, 111.1, 107.7; **HRMS** (ESI) *m/z* found $[M+H]^+$ 121.0763, $C_7H_9N_2^+$ required 121.0760.

4-Vinylpyrimidin-2-amine 2

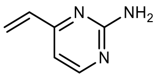

A stirred mixture of 4-chloropyrimidin-2-amine (257 mg, 1.98 mmol), potassium vinyltrifluoroborate (798 mg, 5.96 mmol), Pd(dppf)Cl_2·CH_2Cl_2 (162 mg, 0.198 mmol) and K_2CO_3 (1.65 g, 11.9 mmol) in THF/H_2O (10:1, 6.6 mL) was heated to 70 °C for 16 h. The reaction mixture was filtered through Celite® (eluent EtOAc) and the filtrate was concentrated *in vacuo*. The resulting residue was purified by flash column chromatography (EtOAc/petroleum ether, 2:1) to give vinylpyrimidine **2** (175 mg, 1.44 mmol, 73%) as a light brown solid.

R_f (SiO_2; EtOAc/petroleum ether, 2:1) 0.28; **ν_{max}** (neat/cm^{-1}) 3335, 3173, 1655, 1565; **^{1}H NMR** (CD_3OD, 400 MHz) δ 8.20 (1H, d, *J* = 5.2 Hz), 6.72 (1H, d, *J* = 5.2 Hz), 6.60 (1H, dd, *J* = 17.5, 10.7 Hz), 6.35 (1H, dd, *J* = 17.4, 1.3 Hz), 5.62 (1H, dd, *J* = 10.7, 1.3 Hz); **^{13}C NMR** (CD_3OD,

101 MHz) δ 165.4. 164.7, 159.6, 136.7, 123.0, 108.7; **HRMS** (ESI) *m/z* found $[M+H]^+$ 122.0715, $C_6H_8N_3^+$ required 122.0713.

Data in accordance with literature.[9]

4-Chloro-1,3,5-triazin-2-amine S1

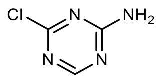

2,4-Dichloro-1,3,5-triazine (500 mg, 3.33 mmol) was added to a stirred solution of 35% aqueous NH_3 (22.2 mL, 402 mmol) at –20 °C, and the resulting solution was stirred for 20 min. The reaction mixture was then extracted with EtOAc/^{i}PrOH (10:1, ×5). The combined organic extracts were washed with brine (×1), dried with Na_2SO_4, and concentrated *in vacuo*. The residue was purified by flash column chromatography (EtOAc/petroleum ether, 2:3) to give aminotriazine **S1** (162 mg, 1.24 mmol, 37%) as a white solid.

R_f (SiO_2; EtOAc/petroleum ether, 1:1) 0.46; **ν_{max}** (neat/cm^{-1}) 3239; 2480, 2396, 2322, 1632, 1500; **^{1}H NMR** (CD_3OD, 400 MHz) δ 8.31 (1H, s); **^{13}C NMR** (CD_3OD, 101 MHz) δ 171.2, 168.7, 168.2; **HRMS** (ESI) *m/z* found $[M+H]^+$ 131.0121, $C_3H_4{}^{35}ClN_4^+$ required 131.0119.

4-Vinyl-1,3,5-triazin-2-amine 3

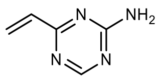

A mixture of 4-chloro-1,3,5-triazin-2-amine **S1** (148 mg, 1.20 mmol), potassium vinyltrifluoroborate (243 mg, 1.82 mmol), Pd(dppf)Cl_2·CH_2Cl_2 (50 mmol, 0.061 mmol) and K_2CO_3 (200 mg, 1.45 mmol) in 1,4-dioxane/H_2O (10:1, 4.5 mL) was stirred at 90 °C for 15 h. The resulting reaction mixture was filtered through Celite® (eluent EtOAc) and the filtrate was concentrated *in vacuo*. The resulting residue was purified by flash column chromatography (EtOAc/petroleum ether, 2:1) to give vinyltriazine **3** (107 mg, 0.876 mmol, 72%) as a white solid.

[9] Walsh, S. J.; Omarjee, S.; Galloway, W. R. J. D.; Kwan, T. T.-L.; Sore, H. F.; Parker, J. S.; Hyvönen, M.; Carroll, J. S.; Spring, D. R. *Chem. Sci.* **2019**, *10*, 694.

R_f (SiO_2; EtOAc/petroleum ether, 4:1) 0.47; **ν_{max}** (neat/cm^{-1}) 3291, 3142, 1689, 1537, 1504, 1433; **^{1}H NMR** (CD_3OD, 400 MHz) δ 8.43 (1H, s), 6.67 (1H, dd, *J* = 17.3, 1.9 Hz), 6.53 (1H, dd, *J* = 17.3, 10.4 Hz), 5.81 (1H, dd, *J* = 10.4, 1.9 Hz); **^{13}C NMR** (CD_3OD, 101 MHz) δ 171.8, 168.1, 167.2, 136.3, 127.6; **HRMS** (ESI) *m/z* found $[M+H]^+$ 123.0668, $C_5H_7N_4^+$ required 123.0665.

6-Vinyl-1,2,4,5-tetrazin-3-amine 4

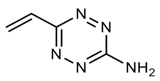

This compound was synthesised based on a literature procedure.[10,11]

To a stirred solution of 3,6-dichloro-1,2,4,5-tetrazine (100 mg, 0.662 mmol) in ^{t}BuOMe (3 mL) at rt, ammonia gas was continuously bubbled from a cannister for 10 min. The reaction mixture was then filtered through Celite®, and the resulting solution was concentrated *in vacuo*. The residue was dissolved in 1,4-dioxane/H_2O (5:1, 4 mL), and potassium vinyltrifluoroborate (124 mg, 0.927 mmol), Brettphos Pd G3 (30 mg, 0.033 mmol) and Cs_2CO_3 (647 mg, 2.00 mmol) was added. The resulting mixture was stirred at 70 °C over 15 h, filtered through Celite®, and the filtrate was concentrated *in vacuo*. The resulting residue was purified by flash column chromatography (petroleum ether/EtOAc, 3:1) to give vinyltetrazine **4** (26 mg, 0.21 mmol, 31% over two steps) as a bright red solid.

R_f (SiO_2; EtOAc/petroleum ether, 1:1) 0.52; **ν_{max}** (neat/cm^{-1}) 3291, 3152, 1618, 1553, 1505; **^{1}H NMR** (CD_3OD, 400 MHz) δ 6.94 (1H, dd, *J* = 17.6, 11.1 Hz), 6.51 (1H, dd, *J* = 17.6, 1.3 Hz), 5.68 (1H, dd, *J* = 11.0, 1.3 Hz); **^{13}C NMR** (CD_3OD, 101 MHz) δ 164.3, 161.1, 131.8, 121.2; **LRMS** (ESI) *m/z* found $[M+H]^+$ 124, $C_4H_6N_5^+$ required 124.

[10] Novák, Z.; Bostai, B.; Csékei, M.; Lőrincz, K.; Kotschy, A. *Heterocycles* **2003**, *60*, 2653.
[11] Bender, A. M.; Chopko, T. C.; Bridges, T. M.; Lindsley, C. W. *Org. Lett.* **2017**, *19*, 5693.

4-(2-((2,6-Difluorobenzyl)thio)ethyl)pyrimidin-2-amine 5

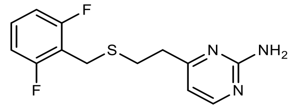

A solution of vinylpyrimidine **2** (112 mg, 0.938 mmol) and (2,6-difluorophenyl)methanethiol (300 mg, 1.88 mmol) in MeOH (5 mL) and aqueous NaPi (pH 8, 500 mM, 0.5 mL) was stirred at rt for 15 h. The reaction mixture was concentrated *in vacuo*, and the resulting residue was diluted with H_2O. The aqueous phase was extracted with CH_2Cl_2 (×3), and the combined organic extracts were dried with Na_2SO_4, and concentrated *in vacuo*. The resulting residue was purified *via* flash column chromatography (EtOAc/petroleum ether, 2:1) to give thioether **5** (248 mg, 0.883 mmol, 94%) as a white solid.

R$_f$ (SiO_2; EtOAc/petroleum ether, 2:1) 0.36; **ν$_{max}$** (neat/cm^{-1}) 3333, 3158, 1662, 1646, 1563; **^{1}H NMR** ($CDCl_3$, 400 MHz) δ 8.18 (1H, d, *J* = 5.0 Hz), 7.21 (1H, tt, *J* = 8.4, 6.4 Hz), 6.95 – 6.83 (2H, m), 6.48 (1H, d, *J* = 5.1 Hz), 5.07 (2H, s), 3.79 (2H, t, *J* = 1.2 Hz), 2.92 – 2.80 (4H, m); **^{13}C NMR** ($CDCl_3$, 101 MHz) δ 169.7, 163.1, 161.3 (dd, *J* = 248.6, 7.9 Hz), 158.3, 128.8 (t, *J* = 10.3 Hz), 115.4 (t, *J* = 19.3 Hz), 111.5 (dd, *J* = 19.0, 6.6 Hz), 111.0, 37.6, 30.5, 22.9 (t, *J* = 2.9 Hz); **^{19}F NMR** ($CDCl_3$, 376 MHz) δ -114.96 (t, *J* = 6.8 Hz); **HRMS** (ESI) $[M+H]^+$ required for $C_{13}H_{14}F_2N_3S^+$ 282.0876, found 282.0886.

4-(2-((2,6-Difluorobenzyl)thio)ethyl)-1,3,5-triazin-2-amine 6

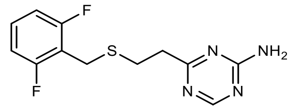

A solution of vinyltriazine **3** (114 mg, 0.938 mmol) and (2,6-difluorophenyl)methanethiol (300 mg, 1.88 mmol) in MeOH (5 mL) and aqueous NaPi (pH 8, 500 mM, 0.5 mL) was stirred at rt for 15 h. The reaction mixture was concentrated *in vacuo*, and the resulting residue was diluted with H_2O. The aqueous phase was extracted with CH_2Cl_2 (×3), and the combined organic extracts were dried with Na_2SO_4, and concentrated *in vacuo*. The resulting residue was purified *via* flash column chromatography (EtOAc/petroleum ether, 2:1) to give thioether **6** (132 mg, 0.468 mmol, 50%) as a white solid.

R$_f$ (SiO_2; EtOAc/petroleum ether, 2:1) 0.29; **ν$_{max}$** (neat/cm^{-1}) 3302, 3171, 3171, 1673, 1626, 1578; **^{1}H NMR** ($CDCl_3$, 400 MHz) δ 8.52 (1H, s), 7.20 (1H, tt, *J* = 8.4, 6.5 Hz), 6.94 – 6.83

(2H, m), 5.42 (2H, s), 3.81 (2H, t, *J* = 1.2 Hz), 3.04 – 2.92 (4H, m); **^{13}C NMR** ($CDCl_3$, 101 MHz) δ 177.8, 166.6, 166.3, 161.3 (dd, *J* = 248.7, 7.9 Hz), 128.7 (t, *J* = 10.3 Hz), 115.4 (t, *J* = 19.3 Hz), 111.5 (dd, *J* = 19.0, 6.6 Hz), 38.4, 29.0, 22.84 (t, *J* = 2.9 Hz); **^{19}F NMR** ($CDCl_3$, 376 MHz) δ -114.93 (t, *J* = 6.7 Hz); **HRMS** (ESI) $[M+H]^+$ required for $C_{12}H_{13}F_2N_4S^+$ 283.0829, found 283.0818.

3-((2,6-Difluorobenzyl)thio)-1-methylpyrrolidine-2,5-dione 7

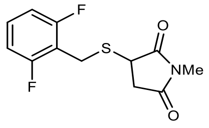

A solution of 1-methyl-1*H*-pyrrole-2,5-dione (100 mg, 0.901 mmol) and (2,6-difluorophenyl)methanethiol (216 mg, 1.35 mmol) in MeOH (5 mL) and aqueous NaPi (pH 8, 500 mM, 0.5 mL) was stirred at rt for 15 h. The reaction mixture was concentrated *in vacuo*, and the resulting residue was purified *via* flash column chromatography (EtOAc/petroleum ether, 1:5) to give thioether **7** (241 mg, 0.888 mmol, 99%) as a light yellow oil.

R_f (SiO_2; EtOAc/petroleum ether, 1:5) 0.14; **ν_{max}** (neat/cm^{-1}) 1694; **^{1}H NMR** ($CDCl_3$, 400 MHz) δ 7.31 – 7.19 (1H, m), 6.97 – 6.86 (2H, m), 4.25 (1H, dt, *J* = 13.6, 1.1 Hz), 3.99 (1H, dt, *J* = 13.6, 1.3 Hz), 3.80 (1H, dd, *J* = 9.1, 4.1 Hz), 3.11 (1H, dd, *J* = 18.7, 9.2 Hz), 3.02 (3H, s), 2.50 (1H, dd, *J* = 18.7, 4.1 Hz); **^{13}C NMR** ($CDCl_3$, 101 MHz) δ 176.5, 174.7, 161.3 (dd, *J* = 249.3, 7.7 Hz), 129.5 (t, *J* = 10.3 Hz), 114.2 (t, *J* = 19.3 Hz), 111.7 (dd, *J* = 19.0, 6.4 Hz), 39.7, 36.2, 25.3, 23.1 (t, *J* = 3.1 Hz); **^{19}F NMR** ($CDCl_3$, 376 MHz) δ -114.46 (t, *J* = 6.8 Hz); **HRMS** (ESI) $[M+H]^+$ required for $C_{12}H_{12}F_2NO_2S^+$ 272.0557, found 272.0546.

4-Chloro-*N*-(2-(2-(prop-2-yn-1-yloxy)ethoxy)ethyl)pyrimidin-2-amine S2

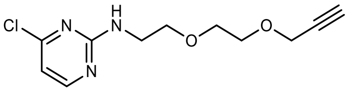

A mixture of 2,4-dichloropyrimidine (458 mg, 3.07 mmol), 2-(2-(prop-2-yn-1-yloxy)ethoxy)ethan-1-amine (400 mg, 2.79 mmol), and Cs_2CO_3 (2.73 g, 8.37 mmol) in dioxane (8 mL) was heated under reflux for 15 h. The resulting suspension was filtered through cotton wool, and the filtrate was concentrated *in vacuo*. The resulting residue was purified *via* flash

column chromatography (EtOAc/petroleum ether, 2:3) to give aminopyrimidine **S2** (181 mg, 0.708 mmol, 25%) as a colourless oil.

R$_f$ (SiO_2; EtOAc/petroleum ether, 1:1) 0.45; **ν$_{max}$** (neat/cm^{-1}) 3286, 1573, 1522; **^{1}H NMR** ($CDCl_3$, 400 MHz) δ 8.14 (1H, d, *J* = 5.2 Hz), 6.55 (1H, d, *J* = 5.1 Hz), 5.80 (1H, br s), 4.21 (2H, d, *J* = 2.4 Hz), 3.72 – 3.59 (8H, m), 2.46 (1H, t, *J* = 2.4 Hz); **^{13}C NMR** ($CDCl_3$, 101 MHz) δ 162.4, 161.4, 159.2, 110.1, 79.7, 74.8, 70.3, 69.8, 69.2, 58.6, 41.3; **HRMS** (ESI) $[M+H]^+$ required for $C_{11}H_{15}{}^{35}ClN_3O_2^+$ 256.1505, found 255.9449.

***N*-(2-(2-(Prop-2-yn-1-yloxy)ethoxy)ethyl)-4-vinylpyrimidin-2-amine 8**

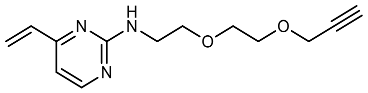

A mixture of **S2** (592 mg, 2.31 mmol), potassium vinyltrifluoroborate (930 mg, 6.94 mmol), K_2CO_3 (1.92 g, 13.9 mmol) and $Pd(dppf)Cl_2{\cdot}CH_2Cl_2$ (189 mg, 0.232 mmol) in THF/H_2O (10:1, 8 mL) was stirred at 70 °C for 15 h. The resulting suspension was cooled to rt, and EDTA (300 mg, 0.721 mmol) was added. The solution was stirred at rt for 1 h, filtered through Celite®, and the filtrate was concentrated *in vacuo*. The resulting residue was purified *via* flash column chromatography (EtOAc/petroleum ether, 1:2) to give vinylpyrimidine **8** (97 mg, 0.39 mmol, 17%) as a colourless oil.

R$_f$ (SiO_2; EtOAc/petroleum ether, 1:1) 0.40; **ν$_{max}$** (neat/cm^{-1}) 3290, 1571; **^{1}H NMR** ($CDCl_3$, 500 MHz) δ 8.24 (1H, d, *J* = 5.0 Hz), 6.62 – 6.51 (2H, m), 6.34 (1H, dd, *J* = 17.4, 1.5 Hz), 5.57 (1H, dd, *J* = 10.6, 1.5 Hz), 5.50 (1H, s), 4.22 (2H, d, *J* = 2.4 Hz), 3.74 – 3.62 (8H, m), 2.45 (1H, t, *J* = 2.4 Hz); **^{13}C NMR** ($CDCl_3$, 126 MHz) δ 163.2, 162.5, 158.6, 136.0, 121.8, 108.0; 79.7, 74.8, 70.3, 70.2, 69.2, 58.6, 41.2; **HRMS** (ESI) $[M+H]^+$ required for $C_{13}H_{18}N_3O_2^+$ 248.2051, found 248.1396.

N-(2-(2-(2-(2-Azidoethoxy)ethoxy)ethoxy)ethyl)-7-nitrobenzo[*c*][1,2,5]oxadiazol-4-amine S3

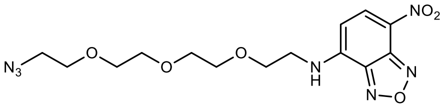

A solution of 4-chloro-7-nitrobenzo[*c*][1,2,5]oxadiazole (200 mg, 1.00 mmol), 2-(2-(2-(2-azidoethoxy)ethoxy)ethoxy)ethan-1-amine (238 µL, 1.20 mmol) and Et_3N (139 µL, 1.00 mmol) in DMF (6 mL) was stirred at rt for 4 h. The resulting solution was diluted with 3 M LiCl, and extracted with EtOAc (×3). The combined organic extracts were washed sequentially with 1 M aqueous HCl (×3), 3 M aqueous LiCl (×3), saturated aqueous Na_2CO_3 (×3), and brine (×2). The organic extracts were dried with Na_2SO_4, and concentrated *in vacuo* to provide NBD-azide **S3** (280 mL, 0.734 mmol, 73%) as an intensely coloured brown oil.

ν_{max} (neat/cm^{-1}) 2097, 1579; **^{1}H NMR** (CD_3OD, 400 MHz) δ 8.38 (1H, d, J = 8.9 Hz), 6.32 (1H, d, J = 8.9 Hz), 3.79 (2H, t, J = 5.1 Hz), 3.70 – 3.53 (12H, m), 3.37 – 3.27 (1H, app m, overlapping with solvent peak); **^{13}C NMR** (CD_3OD, 101 MHz) δ 146.7, 145.7, 145.4, 138.3, 123.1, 100.1, 71.65 , 71.59, 71.54, 71.47 , 71.1, 69.8, 51.7, 44.8; **HRMS** (ESI) $[M+H]^+$ required for $C_{14}H_{20}N_7O_6^+$ 382.1470, found 382.1429.

7-Nitro-N-(2-(2-(2-(2-(4-((2-(2-((4-vinylpyrimidin-2-yl)amino)ethoxy)ethoxy)methyl)-1H-1,2,3-triazol-1-yl)ethoxy)ethoxy)ethoxy)ethyl)benzo[*c*][1,2,5]oxadiazol-4-amine 9

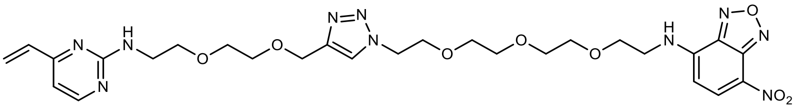

A solution of **8** (24 mg, 0.097 mmol), **S3** (56 mg, 0.147 mmol), $CuSO_4 \cdot 5H_2O$ (30 mg, 0.12 mmol), sodium ascorbate (100 mg, 0.500 mmol) and THPTA (130 mg, 0.300 mmol) in $^{t}BuOH/H_2O/CH_2Cl_2$ (1:1:1, 6 mL) was stirred at rt for 15 h. To this solution, EDTA (100 mg, 0.240 mmol) was added, and the solution was stirred at rt for 1 h. The resulting solution was concentrated under a stream of N_2, and the resulting residue was purified by reverse phase flash column chromatography (10 – 90% solvent B in solvent A. Solvent A: 0.1 M aqueous ammonium hydroxide. Solvent B: MeCN) and lyophilised to give vinylpyrimidine-NBD **9** (25 mg, 0.040 mmol, 41%) as a brown oil.

^{1}H NMR (CD_3OD, 700 MHz) δ 8.48 (1H, d, *J* = 8.7 Hz), 8.16 (1H, d, *J* = 5.1 Hz), 8.01 (1H, s), 6.61 (1H, d, *J* = 5.1 Hz), 6.55 (1H, dd, *J* = 17.4, 10.7 Hz), 6.38 (1H, d, *J* = 8.8 Hz), 6.33 (1H, d, *J* = 17.4 Hz), 5.57 (1H, dd, *J* = 10.6, 1.4 Hz), 4.60 (2H, s), 4.53 (2H, t, *J* = 5.1 Hz), 3.84 (2H, t, *J* = 5.0 Hz), 3.78 (2H, t, *J* = 5.2 Hz), 3.71 – 3.62 (10H, m), 3.58 – 3.52 (8H, m); **^{13}C NMR** (CD_3OD, 176 MHz) δ 164.8, 163.6, 159.4, 146.7, 145.9, 145.7, 145.5, 138.5, 136.9, 125.9, 123.2, 122.6, 108.5, 100.2, 71.6, 71.44, 71.39, 71.3, 70.84, 70.78, 70.3, 69.8, 65.0, 51.4, 44.8, 42.0; **HRMS** (ESI) $[M+H]^+$ required for $C_{27}H_{37}N_{10}O_8^+$ 629.2769, found 629.2819.

_N_-(2-(2-(2-(2-azidoethoxy)ethoxy)ethoxy)ethyl)-5-((3a*R*,4*R*,6a*S*)-2-oxohexahydro-1*H*-thieno[3,4-d]imidazol-4-yl)pentanamide 12

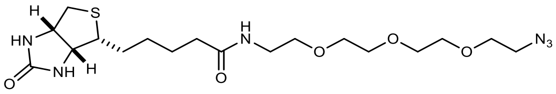

A solution of biotin (460 mmol, 1.89 mmol), EDC·HCl (725 mg, 3.78 mmol), DMAP (460 mg, 3.76 mmol) and $^i Pr_2EtN$ (983 µL, 5.65 mmol) in DMF (15 mL) was stirred at rt for 20 min. To this solution, 2-(2-(2-(2-azidoethoxy)ethoxy)ethoxy)ethan-1-amine (557 µL, 2.81 mmol) was added before stirring at rt for 15 h. The solution was concentrated *in vacuo* and the resulting residue was purified *via* flash column chromatography (neat CH_2Cl_2 with 1% AcOH → 1:10 MeOH/CH_2Cl_2 with 1% AcOH) to give biotin-azide **12** (686 mg, 1.54 mmol, 84%) as a white solid.

R_f (SiO_2; CH_2Cl_2/MeOH, 10:1) 0.30; **ν_{max}** (neat/cm^{-1}) 2103, 1682, 1639; **^{1}H NMR** (CD_3OD, 400 MHz) δ 4.50 (1H, ddd, *J* = 7.9, 5.0, 1.0 Hz), 4.31 (1H, dd, *J* = 7.9, 4.4 Hz), 3.71 – 3.60 (10H, m), 3.55 (2H, t, *J* = 5.5 Hz), 3.40 – 3.34 (4H, m), 3.21 (1H, ddd, *J* = 8.8, 5.9, 4.5 Hz), 2.93 (1H, dd, *J* = 12.8, 5.0 Hz), 2.71 (1H, d, *J* = 12.7 Hz), 2.22 (2H, t, *J* = 7.4 Hz), 1.80 – 1.53 (4H, m), 1.44 (2H, qn, *J* = 7.5 Hz); **^{13}C NMR** (CD_3OD, 101 MHz) δ 174.7, 164.7, 70.24, 70.21, 70.1, 69.9, 69.7, 69.2, 62.0, 60.2, 55.6, 50.4, 39.7, 39.0, 35.3, 28.4, 28.1, 25.5; **HRMS** (ESI) $[M+Na]^+$ required for $C_{18}H_{32}N_6O_5SNa^+$ 467.2047, found 467.1966.

5-((3a*S*,4*S*,6a*R*)-2-Oxohexahydro-1*H*-thieno[3,4-*d*]imidazol-4-yl)-N-(2-(2-(2-(2-(4-((2-(2-((4-vinylpyrimidin-2-yl)amino)ethoxy)ethoxy)methyl)-1*H*-1,2,3-triazol-1-yl)ethoxy)ethoxy)ethoxy)ethyl)pentanamide 10

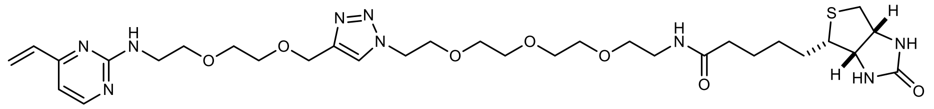

A solution of **8** (30 mg, 0.12 mmol), **12** (107 mg, 0.243 mmol) $CuSO_4{\cdot}5H_2O$ (37 mg, 0.15 mmol), THPTA (158 mg, 0.364 mmol) and sodium ascorbate (121 mg, 0.607 mmol) in $^{t}BuOH/CH_2Cl_2/H_2O$ (1:1:1, 6 mL) was stirred at rt for 15 h. To this solution, EDTA (100 mg, 0.240 mmol) was added, and the solution was stirred at rt for 1 h. The resulting solution was concentrated under a stream of N_2, and the resulting residue was purified by reverse phase flash column chromatography (10 – 90% solvent B in solvent A. Solvent A: 0.1 M aqueous ammonium hydroxide. Solvent B: MeCN) and lyophilised to give vinylpyrimidine-biotin **10** (19 mg, 0.028 mmol, 23%) as a white solid.

^{1}H NMR (CD_3OD, 400 MHz) δ 8.20 (1H, d, *J* = 5.1 Hz), 8.04 (1H, s), 6.66 (1H, d, *J* = 5.2 Hz), 6.60 (1H, dd, *J* = 17.4, 10.6 Hz), 6.37 (1H, dd, *J* = 17.5, 1.5 Hz), 5.60 (1H, dd, *J* = 10.7, 1.5 Hz), 4.64 (2H, s), 4.58 (2H, dd, *J* = 5.6, 4.6 Hz), 4.48 (1H, ddd, *J* = 7.9, 5.0, 1.0 Hz), 4.29 (1H, dd, *J* = 7.9, 4.4 Hz), 3.89 (2H, dd, *J* = 5.6, 4.6 Hz), 3.70 – 3.57 (16H, m), 3.52 (2H, t, *J* = 5.5 Hz), 3.34 (2H, t, *J* = 5.5 Hz), 3.19 (1H, ddd, *J* = 8.9, 5.9, 4.4 Hz), 2.91 (1H, dd, *J* = 12.7, 5.0 Hz), 2.69 (1H, d, *J* = 12.7 Hz), 2.19 (2H, t, *J* = 7.5 Hz), 1.79 – 1.52 (4H, m), 1.49 – 1.34 (2H, m); **^{13}C NMR** (CD_3OD, 101 MHz) δ 176.1, 166.1, 164.9, 163.7, 159.5, 145.8, 137.0, 125.9, 122.6, 108.5, 71.55, 71.46, 71.4, 71.3, 71.2, 70.84, 70.82, 70.6, 70.4, 65.1, 63.4, 61.6, 57.0, 51.4, 42.0, 41.0, 40.3, 36.7, 29.8, 29.5, 26.8; **HRMS** (ESI) $[M+H]^+$ required for $C_{31}H_{50}N_9O7S^+$ 692.3549, found 692.3698.

4-Chloro-*N*-(2-(2-(prop-2-yn-1-yloxy)ethoxy)ethyl)-1,3,5-triazin-2-amine S4

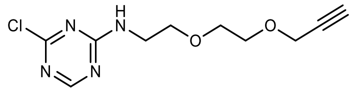

2-(2-(Prop-2-yn-1-yloxy)ethoxy)ethan-1-amine (99 µL, 0.698 mmol) was added to a solution of 2,4-dichloro-1,3,5-triazine (104 mg, 0.698 mmol) and $^{i}Pr_2EtN$ (365 µL, 0.838 mmol) in THF (5 mL) at 0 °C. The resulting solution was stirred at 0 °C for 2 h. The solution was concentrated *in vacuo*, and the resulting residue was purified *via* flash column chromatography

(EtOAc/petroleum ether, 1:2) to give aminotriazine **S4** (90 mg, 0.362 mmol, 52%) as a colourless oil.

R$_f$ (SiO_2; EtOAc/petroleum ether, 1:1) 0.22; **ν$_{max}$** (neat/cm^{-1}) 3279, 2118, 1587, 1561; **^{1}H NMR** ($CDCl_3$, 400 MHz, mixture of rotamers) δ 8.37 (1H, app d, *J* = 29.6 Hz), 6.40 (1H, app d, *J* = 73.1 Hz), 4.22 (2H, app dd, *J* = 2.4, 1.3 Hz), 3.76 – 3.62 (8H, m), 2.46 (1H, app td, *J* = 2.4, 1.0 Hz); **^{13}C NMR** ($CDCl_3$, 101 MHz, mixture of rotamers) δ 170.9, 170.1, 167.4, 166.7, 165.7, 165.6, 79.54, 79.53, 74.9, 70.5, 70.4, 69.31, 69.29, 69.17, 69.16, 58.58, 58.58, 41.2, 41.0; **HRMS** (ESI) $[M+H]^+$ required for $C_{10}H_{14}{}^{35}ClN_4O_2{}^+$ 257.0800, found 257.0795.

N-(2-(2-(Prop-2-yn-1-yloxy)ethoxy)ethyl)-4-vinyl-1,3,5-triazin-2-amine 11

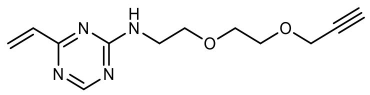

A mixture of **S4** (369 mg, 1.44 mmol), potassium vinyltrifluoroborate (630 mg, 4.71 mmol), Cs_2CO_3 (3.07 g, 9.42 mmol) and $Pd(dppf)Cl_2{\cdot}CH_2Cl_2$ (128 mg, 0.157 mmol) in dioxane/H_2O (10:1, 8 mL) was heated to 90 °C for 15 h. The resulting mixture was cooled to rt, and EDTA (300 mg, 0.721 mmol) was added. The suspension was stirred at rt for 1 h, filtered through Celite®, and the filtrate was concentrated *in vacuo*. The resulting residue was purified *via* flash column chromatography (EtOAc/petroleum ether, 1:1) to give vinyltriazine **11** (200 mg, 0.805 mmol, 56%) as a colourless oil.

R$_f$ (SiO_2; EtOAc) 0.58; **ν$_{max}$** (neat/cm^{-1}) 1591; **^{1}H NMR** ($CDCl_3$, 400 MHz, mixture of rotamers) δ 8.53 (1H, app d, *J* = 31.0 Hz), 6.73 – 6.47 (2H, m), 6.11 (1H, app d, *J* = 44.5 Hz, N*H*), 5.79 (1H, dd, *J* = 10.3, 2.2 Hz), 4.22 (2H, d, *J* = 2.4 Hz), 3.69 (8H, ddd, *J* = 8.6, 6.2, 3.4 Hz), 2.46 (1H, t, *J* = 2.4 Hz); **^{13}C NMR** ($CDCl_3$, 101 MHz, mixture of rotamers) δ 170.8, 170.5, 166.4, 166.1, 165.4, 165.3, 136.0, 135.6, 126.9, 79.6, 74.9, 70.4, 69.7, 69.2, 58.6, 40.8, 40.6.

Azide-PEG3-arylsulfate-MMAE 14

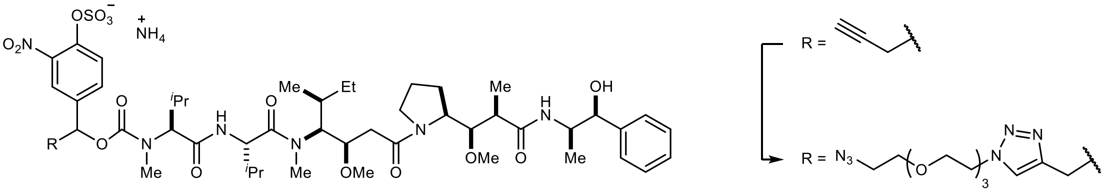

A degassed solution of $CuSO_4{\cdot}5H_2O$ (0.24 mg, 0.95 µmol), THPTA (0.83 mg, 1.9 µmol) and sodium ascorbate (0.76 mg, 3.8 µmol) in H_2O/*t*BuOH (0.2 mL, 1:1) was added to a degassed solution of alkynyl-arylsulfate-MMAE[12] (2.00 mg, 1.91 µmol) and N_3-PEG3-N_3 (1.99 µL, 9.54 µmol) in H_2O/*t*BuOH (0.1 mL, 1:1). The reaction mixture was stirred at rt for 15 min before being purified by reverse-phase flash column chromatography (25-100% solvent B in solvent A. Solvent A: 0.1 M NH_4OH (aq). Solvent B: MeCN) and lyophilised to yield azide **14** (1.56 mg, 1.21 µmol, 63%) as a white solid.

LRMS (ESI) *m/z* found [M-H$^+$] 1274.0 $C_{58}H_{89}N_{12}O_{18}{}^{32}S^{-}$ required 1273.6; **HPLC** (5-95% MeCN/H_2O over 20 min) retention time 12.180 min.

1-(2-(2-(Prop-2-yn-1-yloxy)ethoxy)ethyl)-1*H*-pyrrole-2,5-dione 15

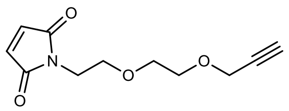

A solution of 2-(2-(prop-2-yn-1-yloxy)ethoxy)ethan-1-amine (122 mg, 0.852 mmol) and maleic anhydride (84 mg, 0.85 mmol) was heated under reflux for 15 h. The resulting solution was concentrated *in vacuo* and purified *via* flash column chromatography (EtOAc/petroleum ether, 1:2) to give maleimide **15** (94 mg, 0.42 mmol, 49%) as a light yellow oil.

R_f (SiO_2; EtOAc/petroleum ether, 1:1) 0.47; **ν_{max}** (neat/cm^{-1}) 1699; **^{1}H NMR** ($CDCl_3$, 400 MHz) δ 6.72 (2H, s), 4.17 (2H, d, J = 2.4 Hz), 3.74 (2H, app td, J = 5.6, 0.9 Hz), 3.67 – 3.62 (6H, m), 2.44 (1H, t, J = 2.4 Hz); **^{13}C NMR (**$CDCl_3$, 101 MHz) δ 170.7, 134.2, 79.7, 74.6, 69.9, 69.1, 67.9, 58.4, 37.1; **HRMS** (ESI) [M+H]$^+$ required for $C_{11}H_{14}NO_4{}^+$ 224.0918, found 224.1000.

[12] This compound was synthesised following a literature procedure.
Bargh, J. D.; Walsh, S. J.; Isidro-Llobet, A.; Omarjee, S.; Carroll, J. S.; Spring, D. R. *Chem. Sci.* **2020**, *11*, 2375.

10. Spectra of small molecules

4-Vinylpyridin-2-amine 1

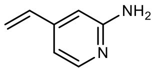

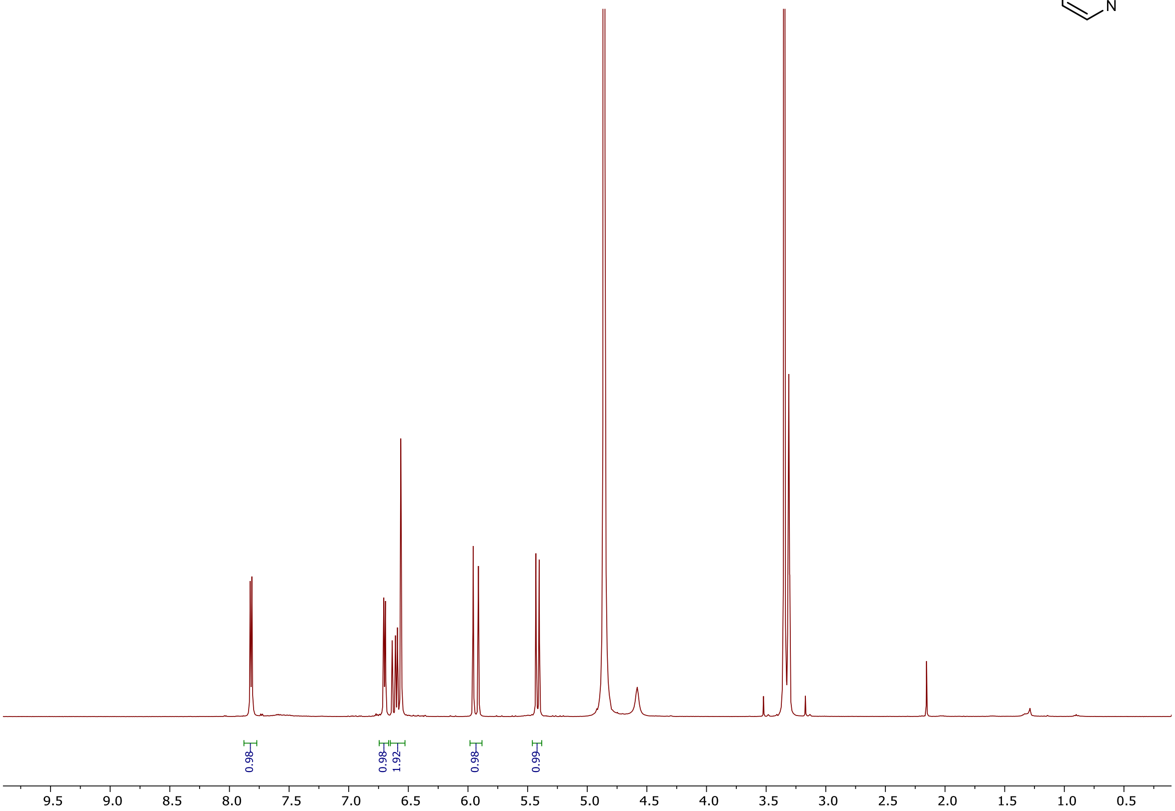

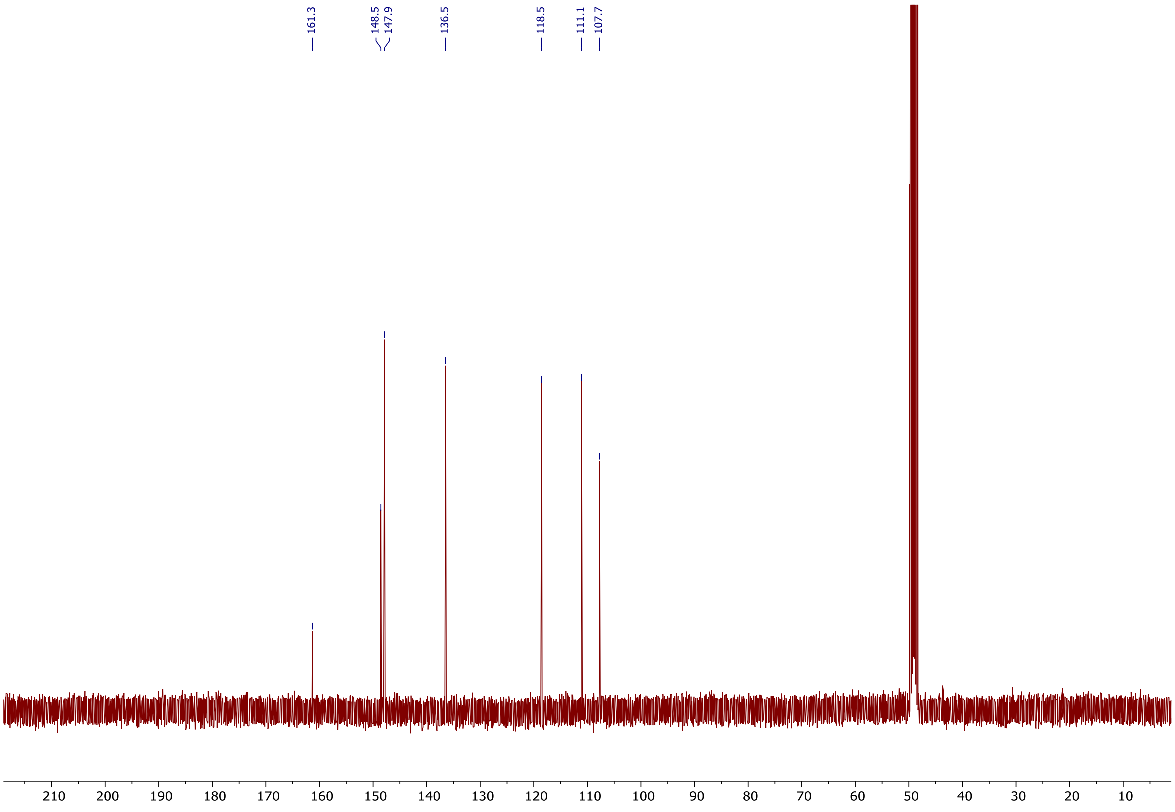

4-Vinylpyrimidin-2-amine 2

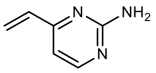

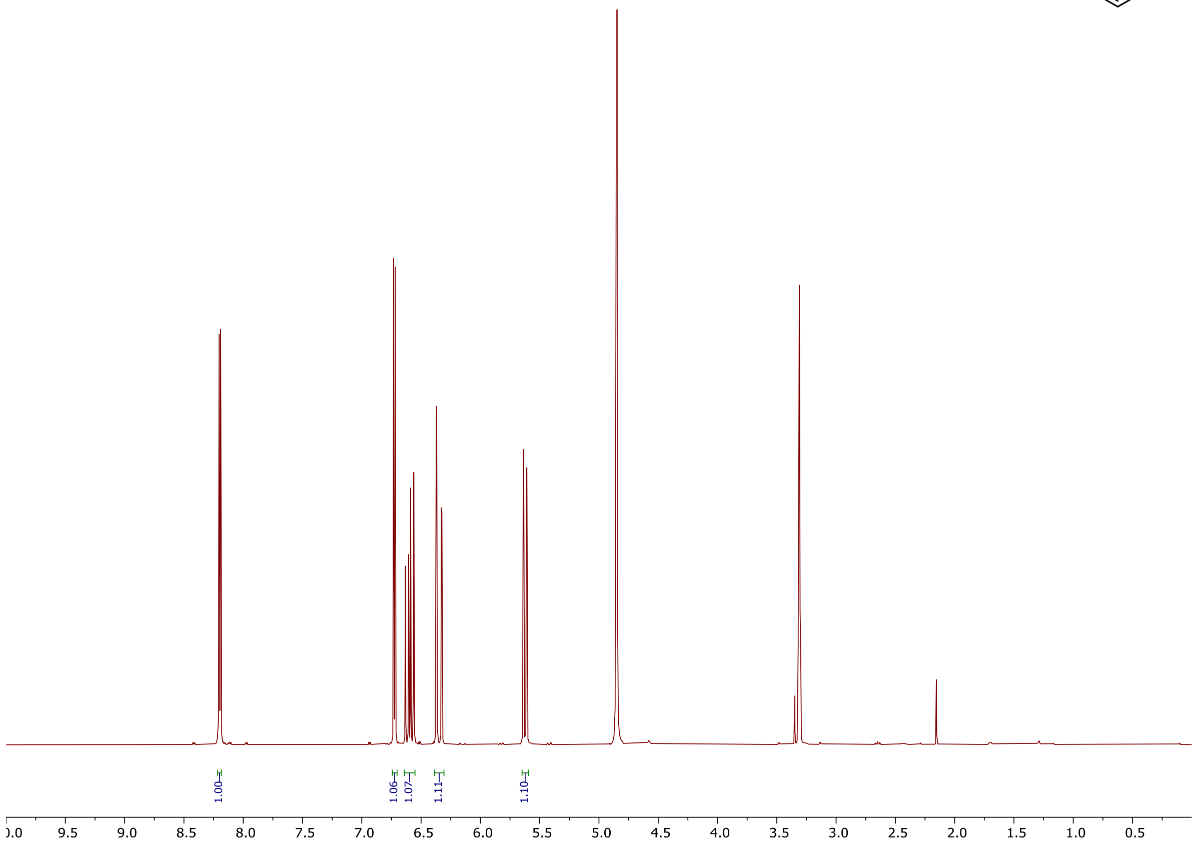

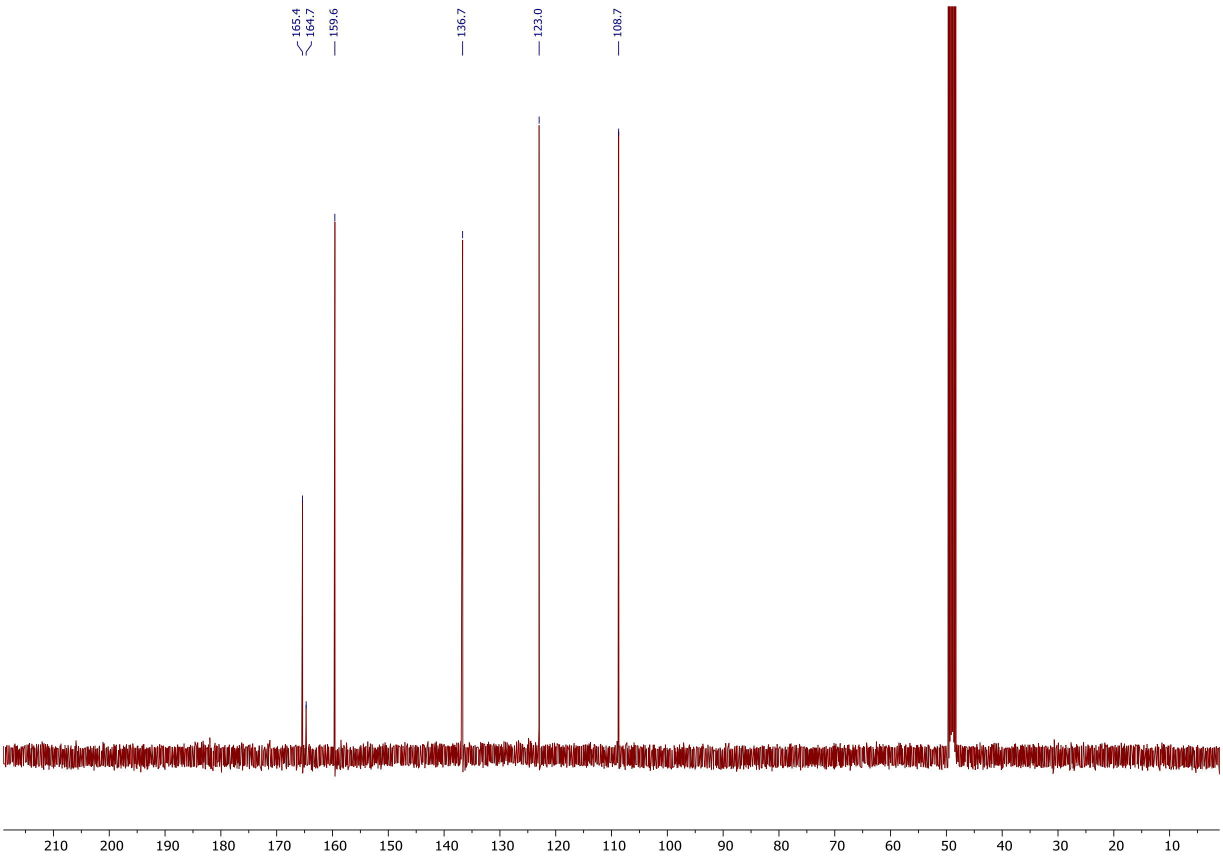

4-Chloro-1,3,5-triazin-2-amine S1

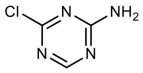

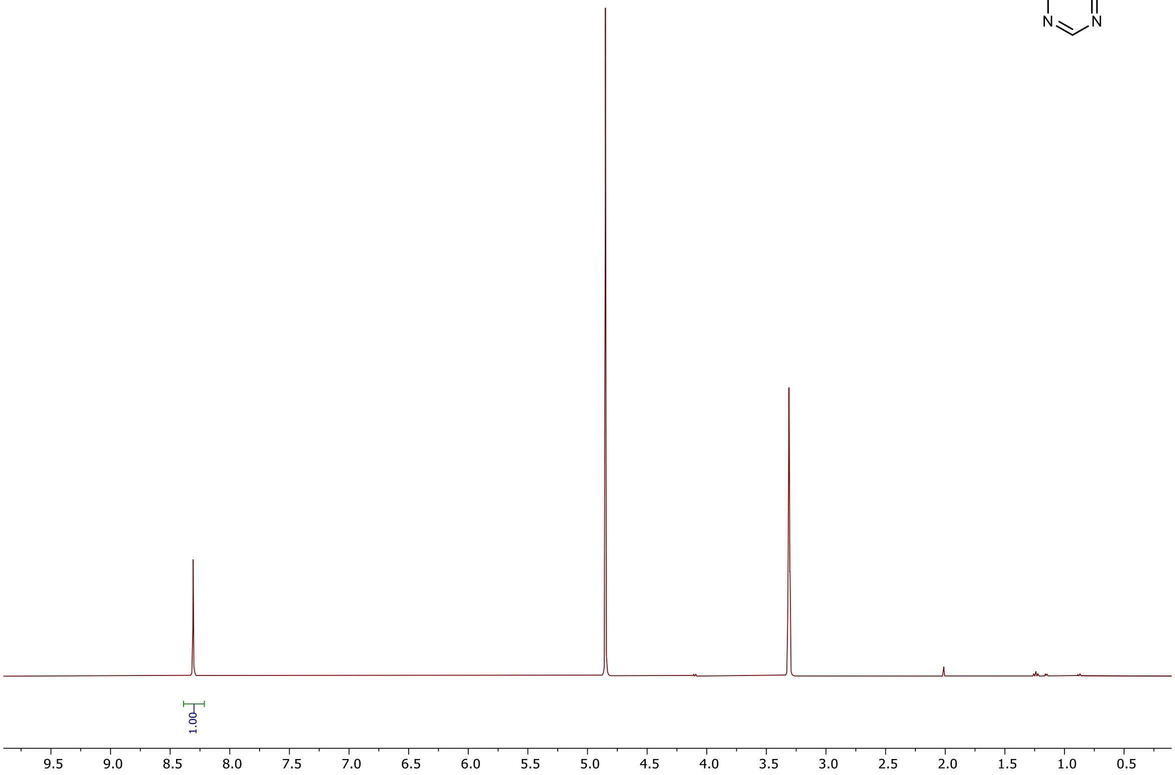

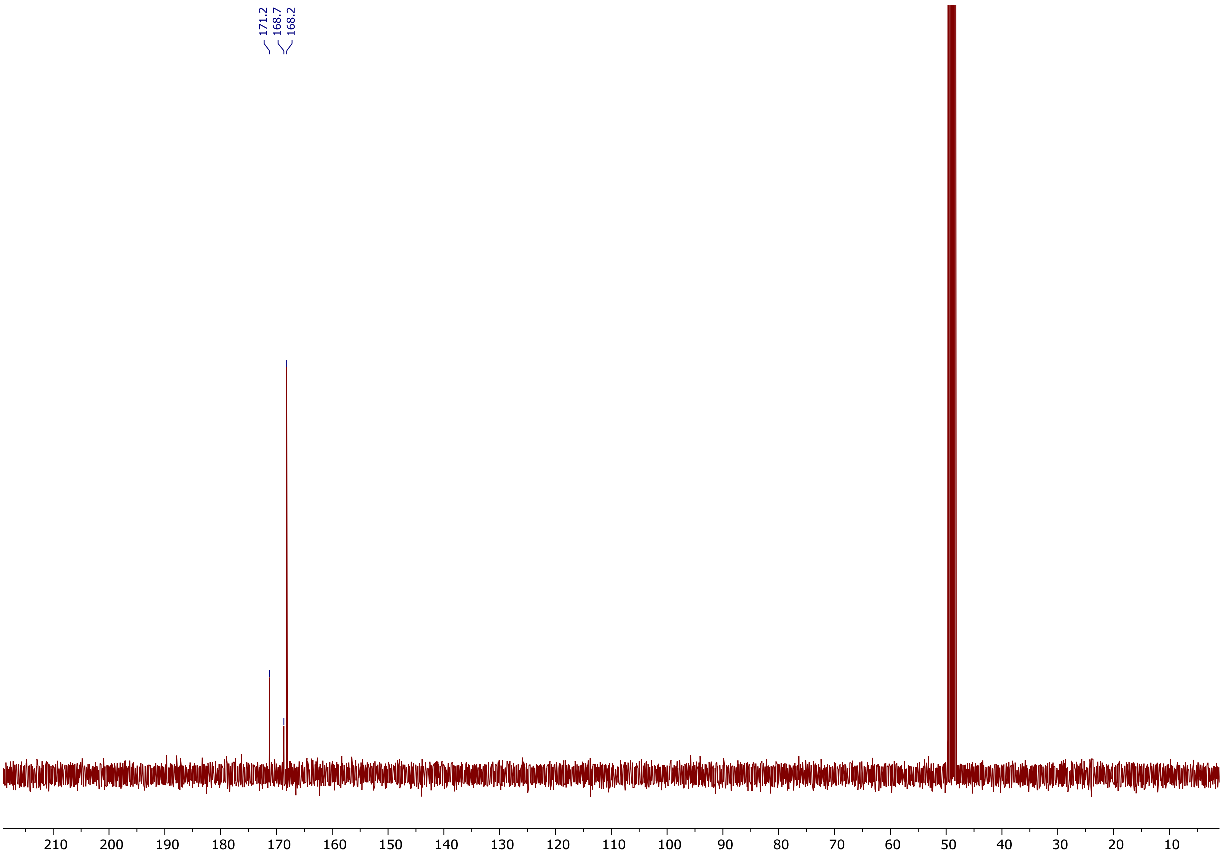

4-Vinyl-1,3,5-triazin-2-amine 3

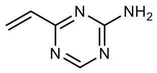

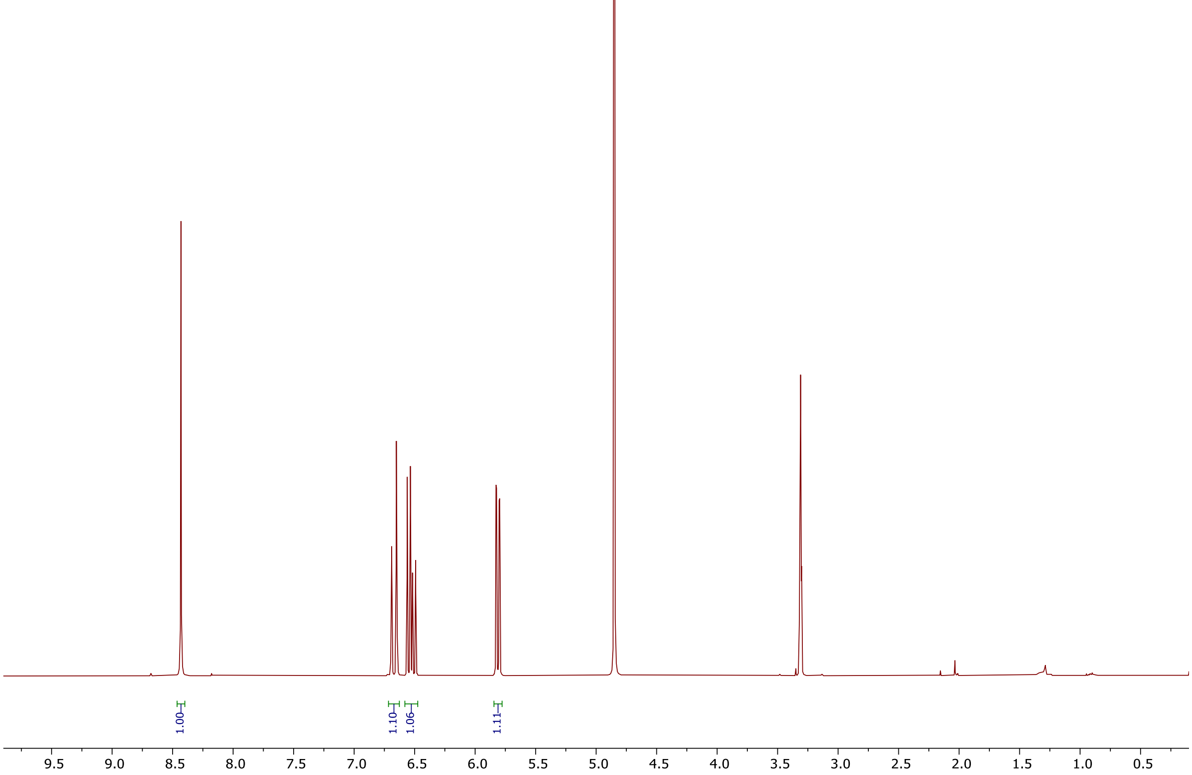

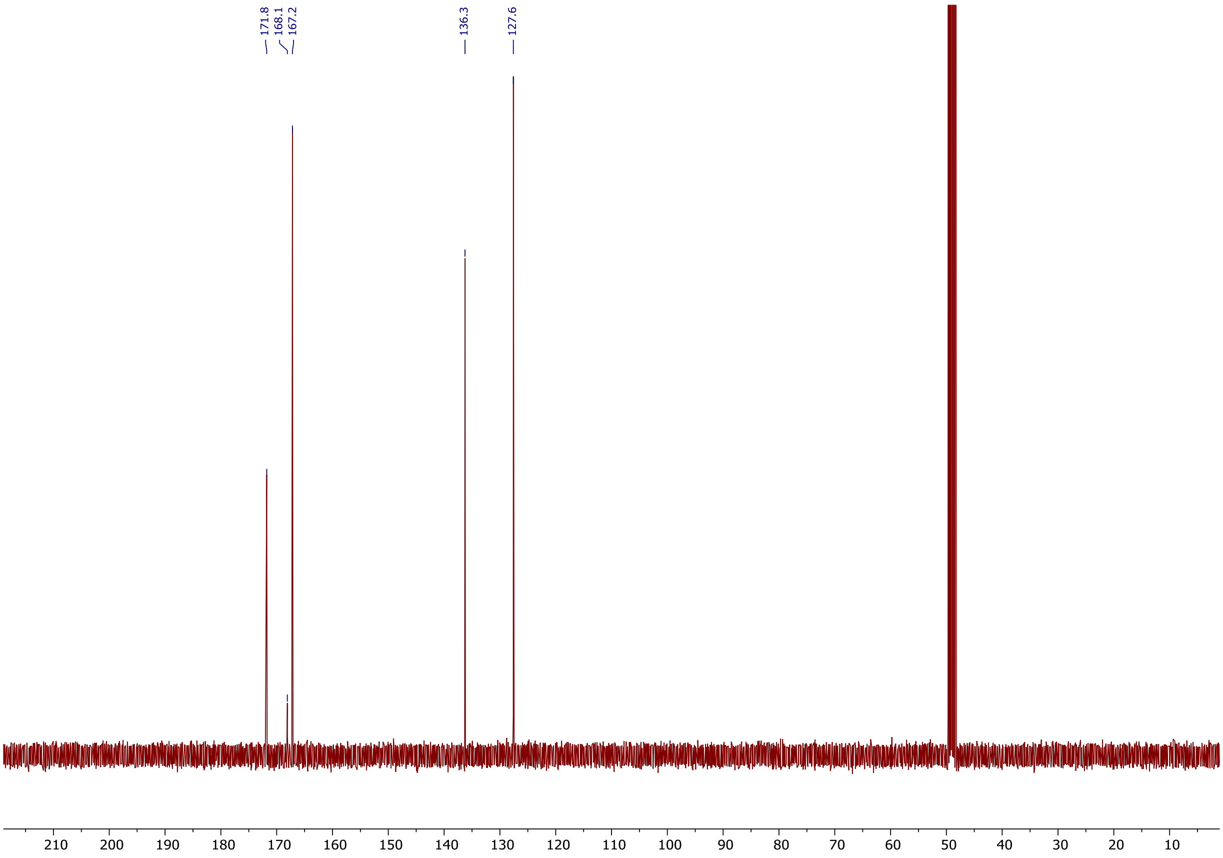

6-Vinyl-1,2,4,5-tetrazin-3-amine 4

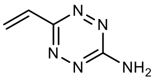

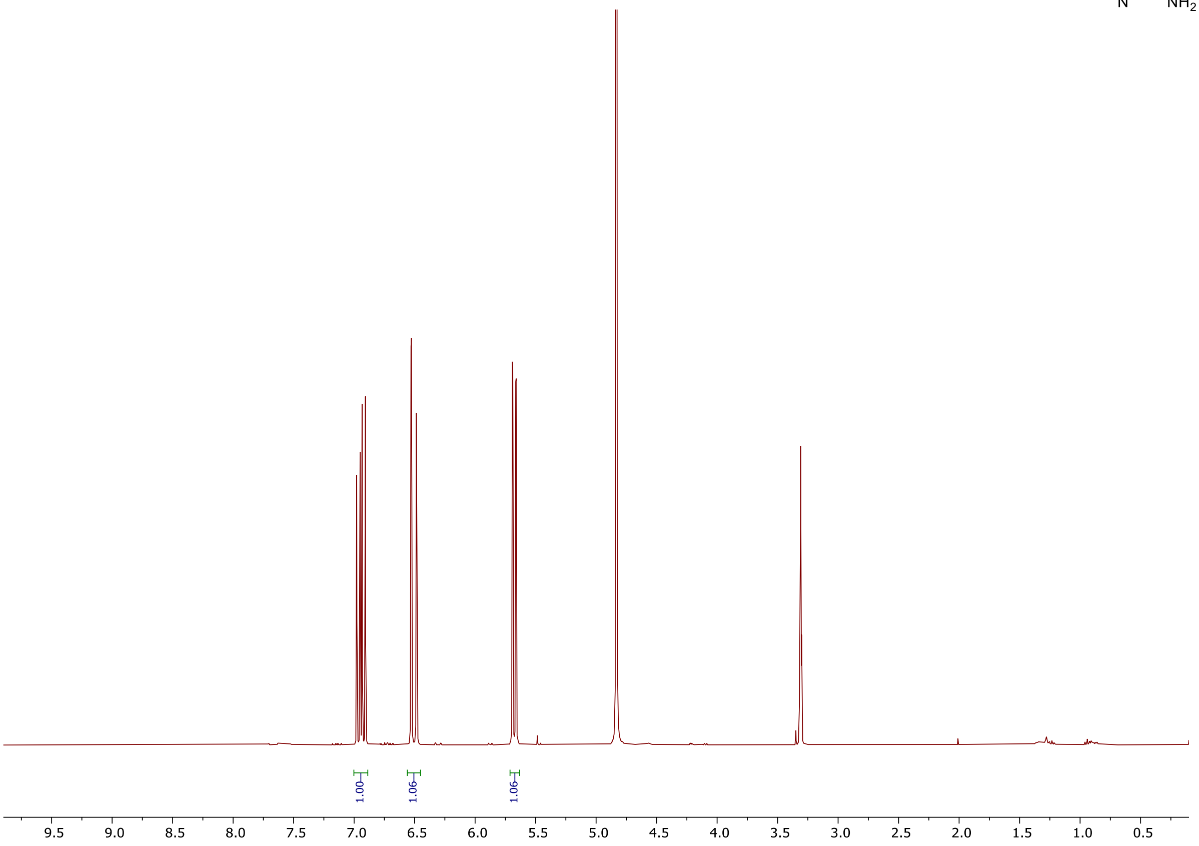

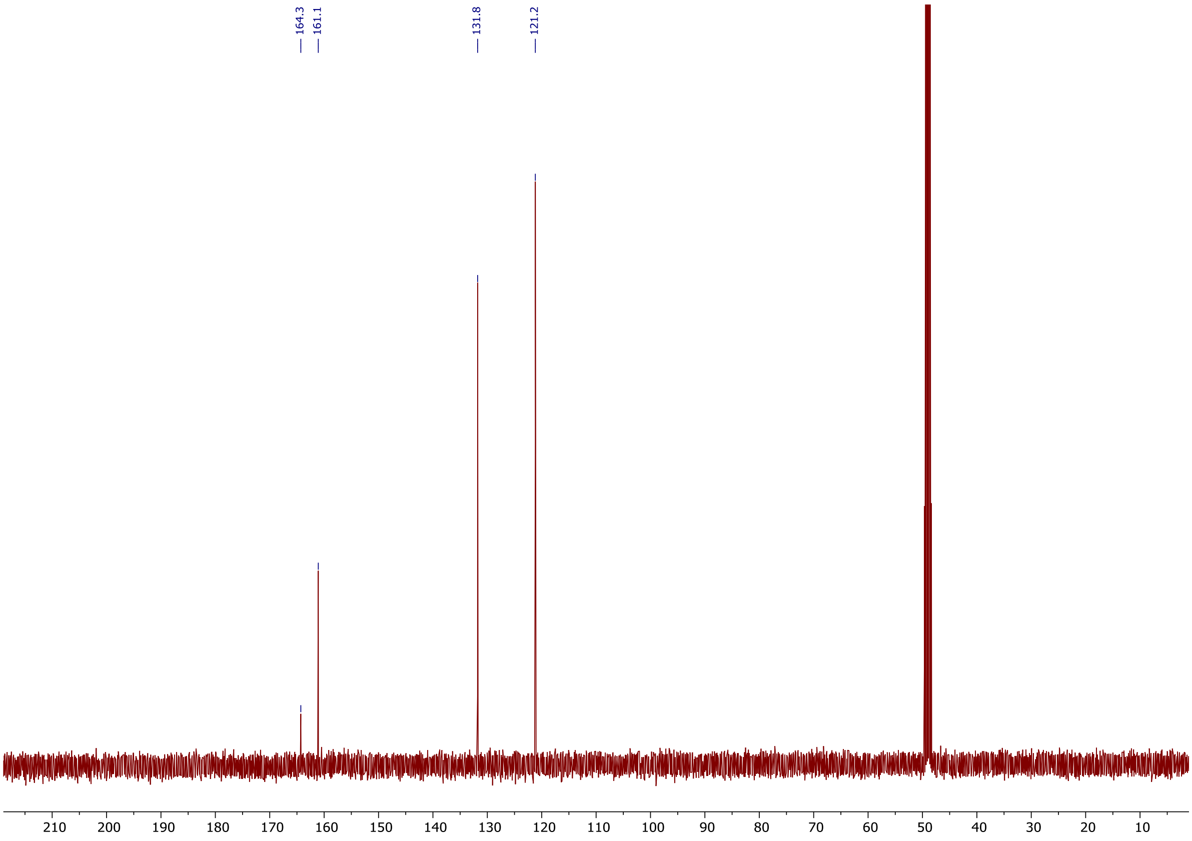

4-(2-((2,6-Difluorobenzyl)thio)ethyl)pyrimidin-2-amine 5

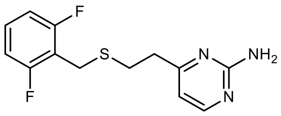

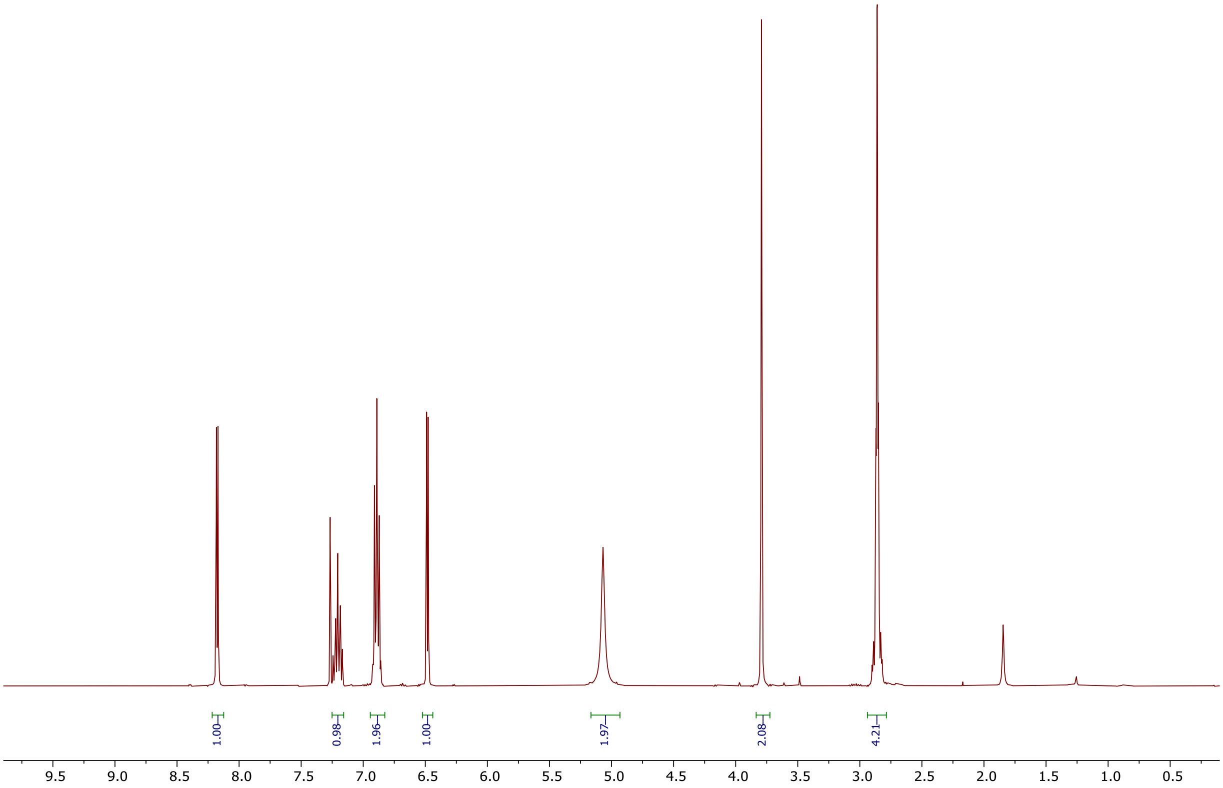

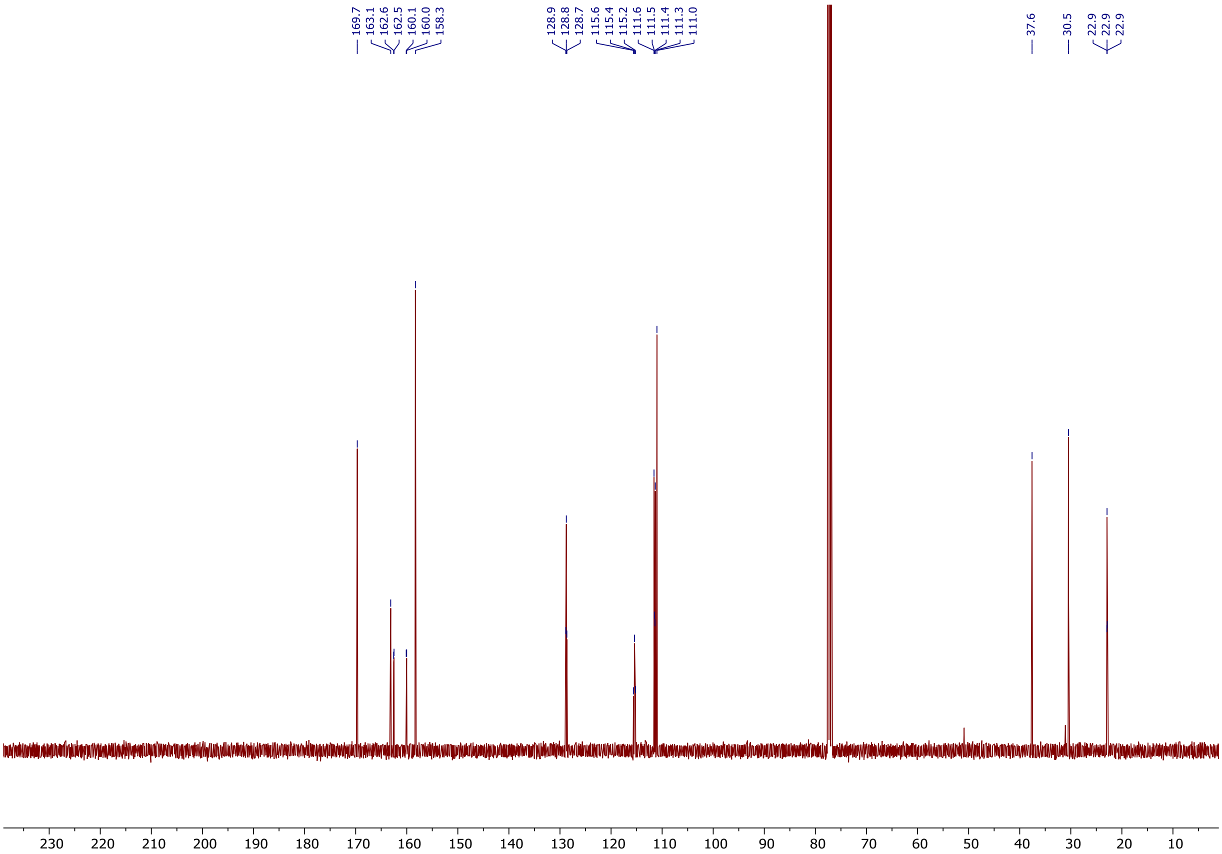

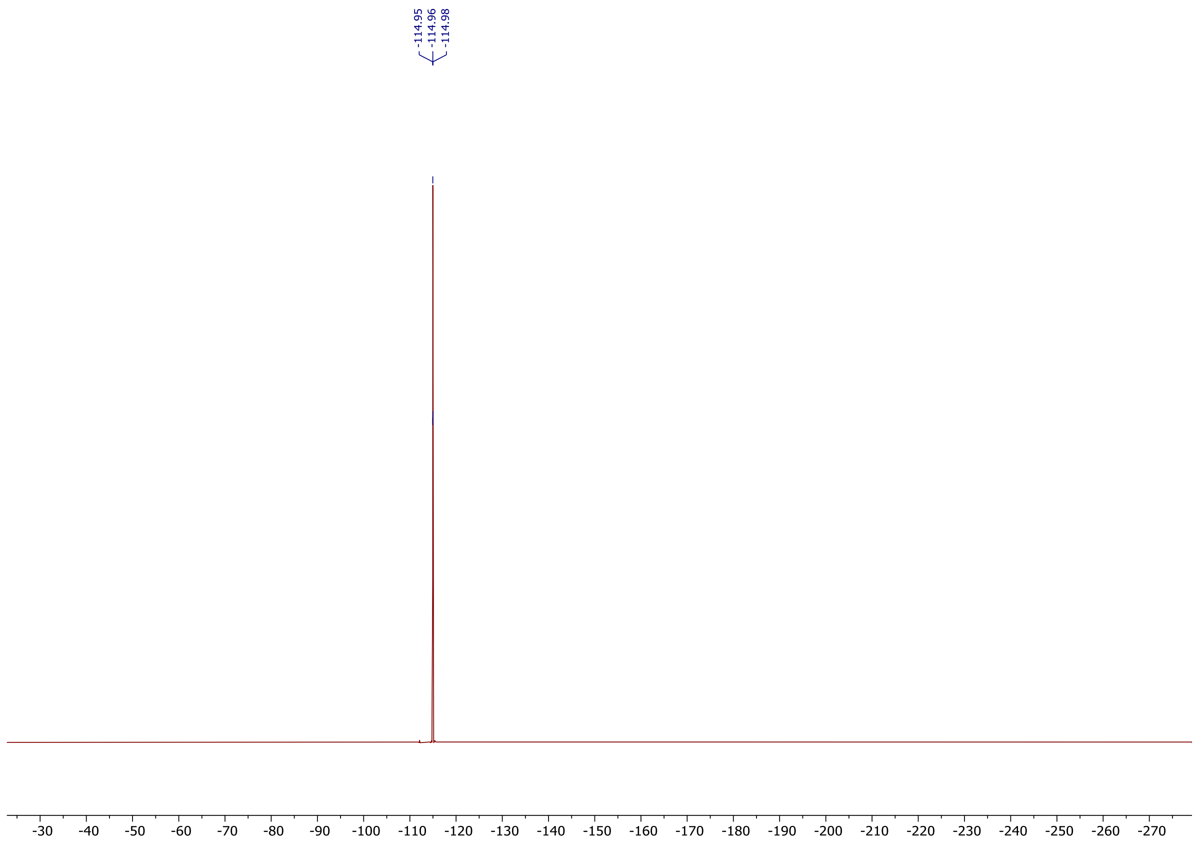

-114.95
-114.96
-114.98
-30 -40 -50 -60 -70 -80 -90 -100 -110 -120 -130 -140 -150 -160 -170 -180 -190 -200 -210 -220 -230 -240 -250 -260 -270

4-(2-((2,6-Difluorobenzyl)thio)ethyl)-1,3,5-triazin-2-amine 6

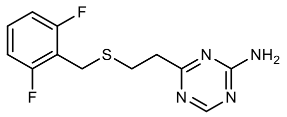

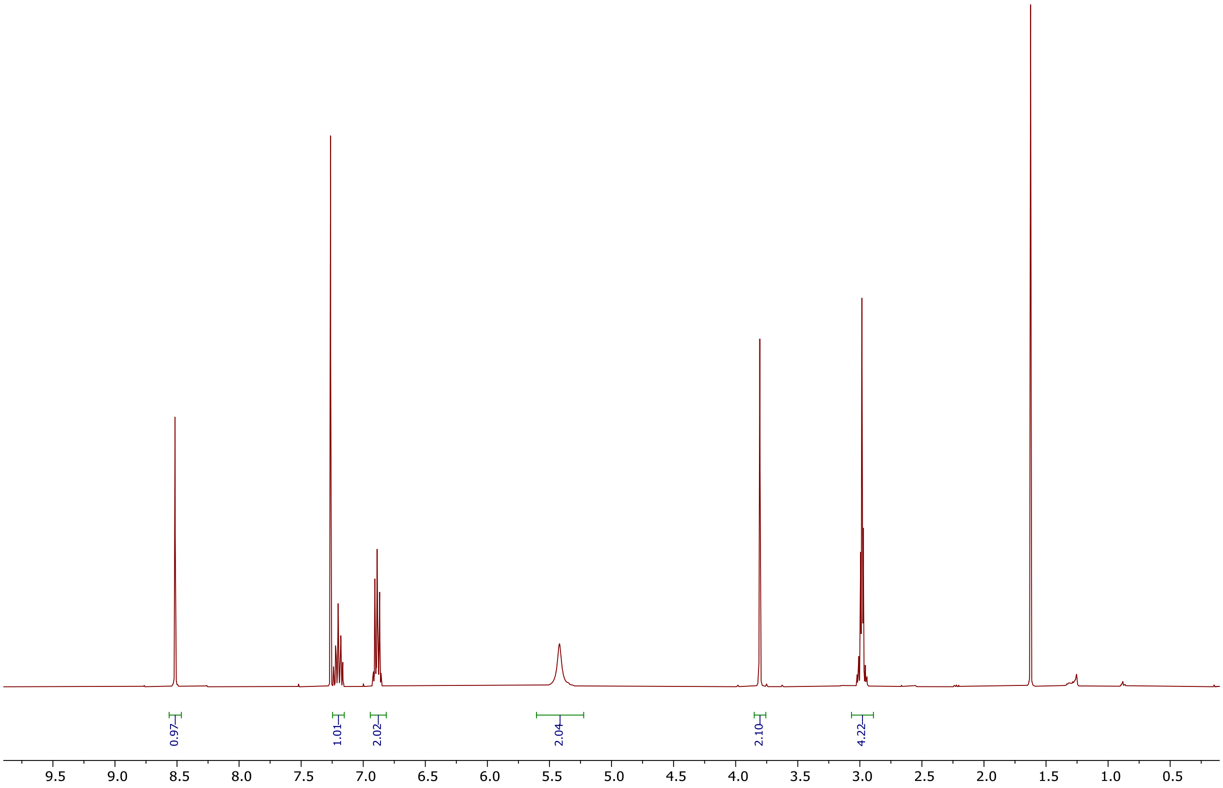

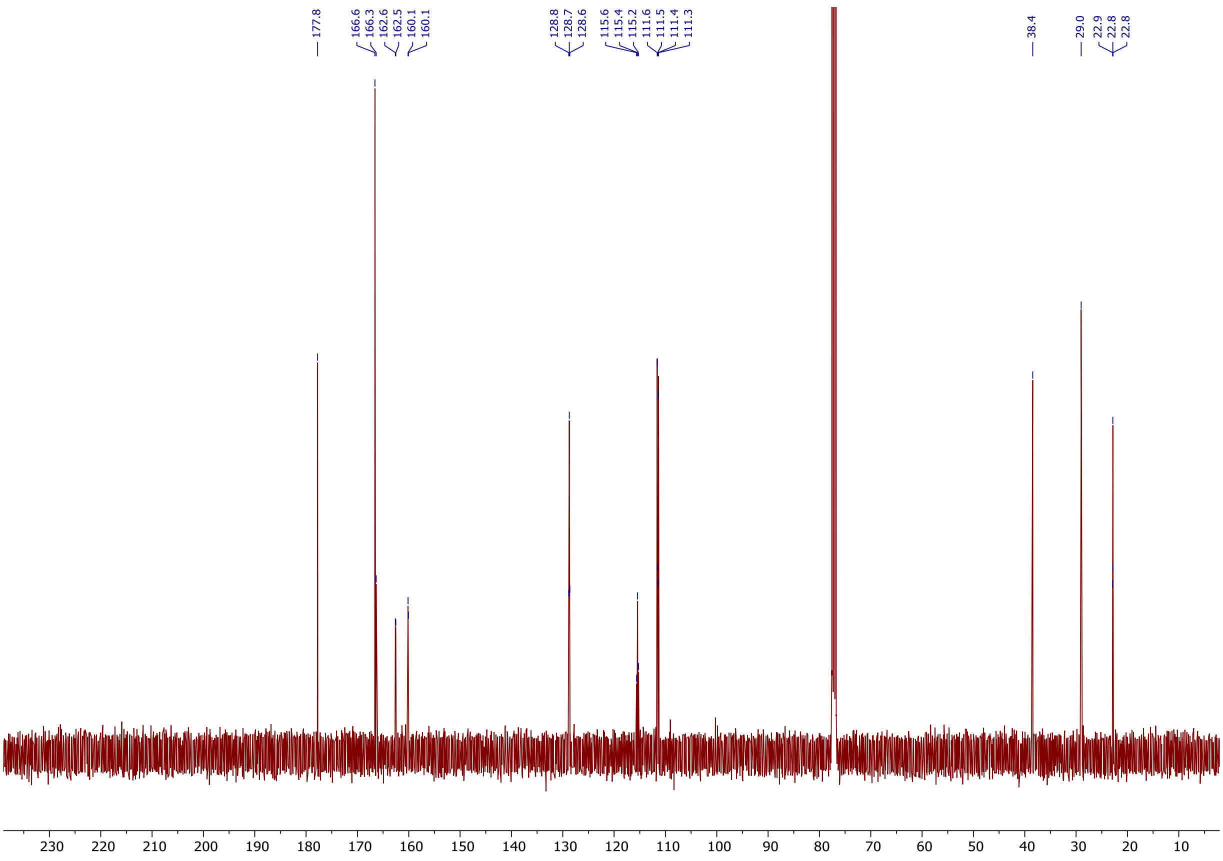

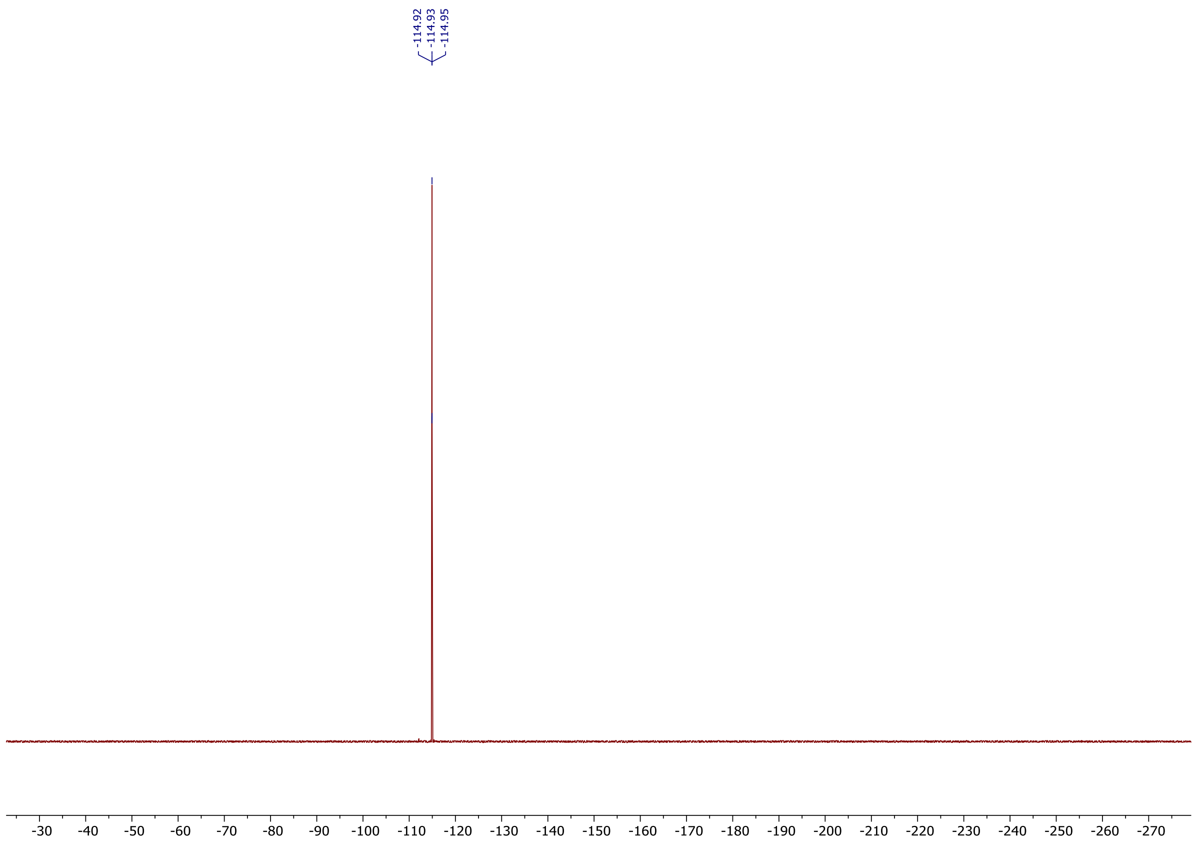
-114.92
-114.93
-114.95
-30 -40 -50 -60 -70 -80 -90 -100 -110 -120 -130 -140 -150 -160 -170 -180 -190 -200 -210 -220 -230 -240 -250 -260 -270

3-((2,6-Difluorobenzyl)thio)-1-methylpyrrolidine-2,5-dione 7

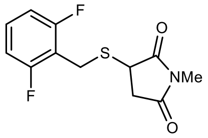

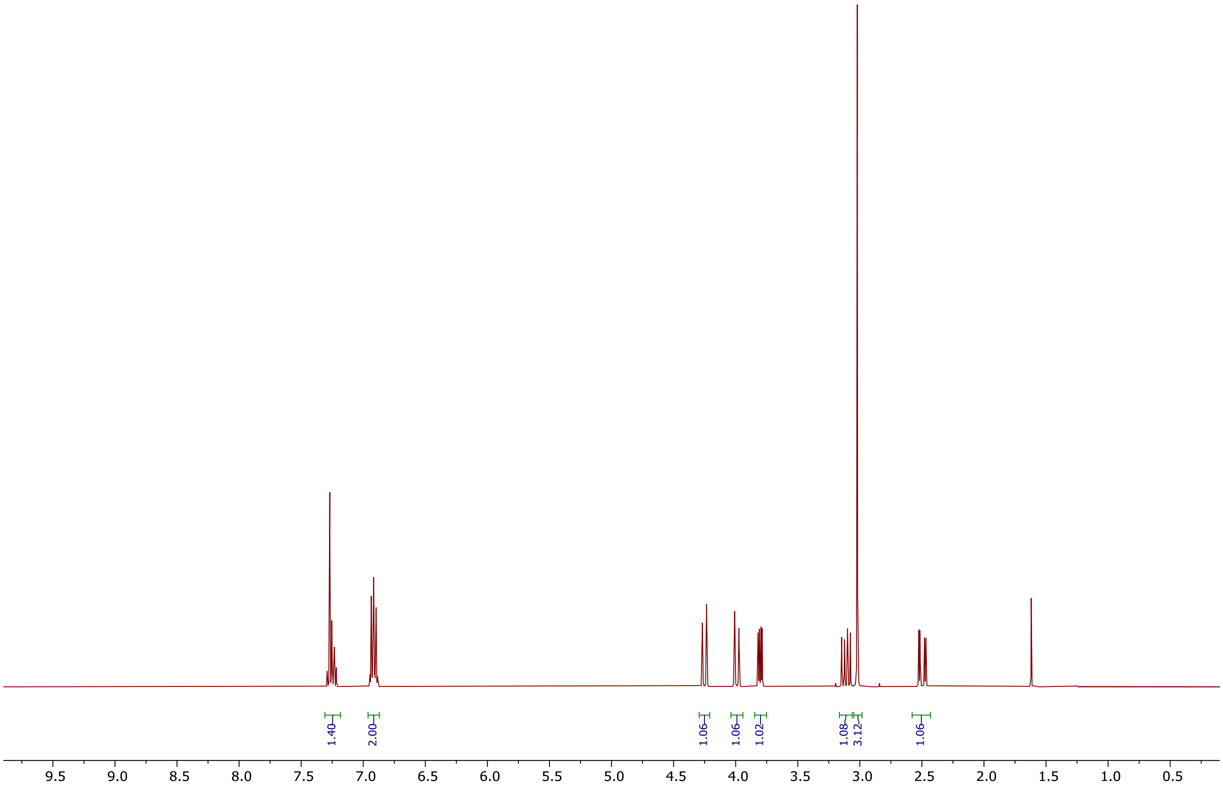

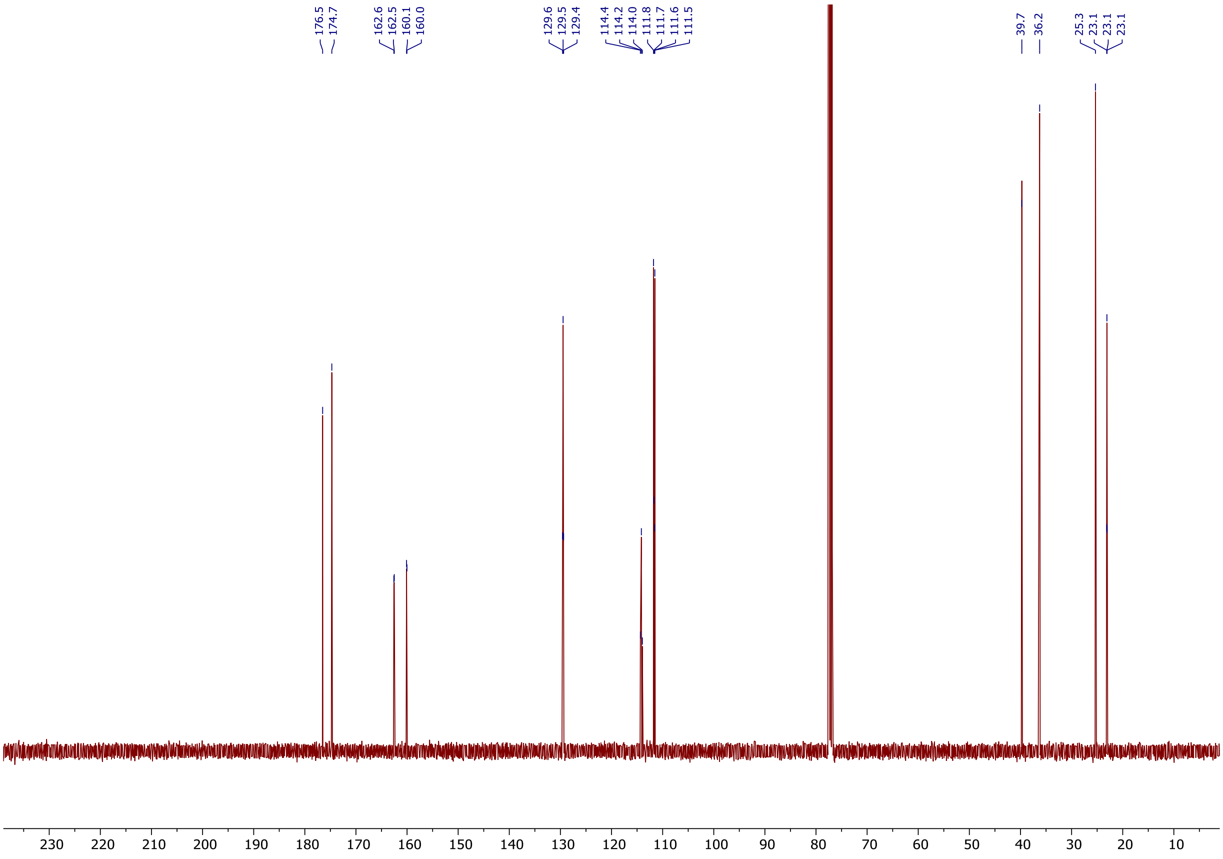

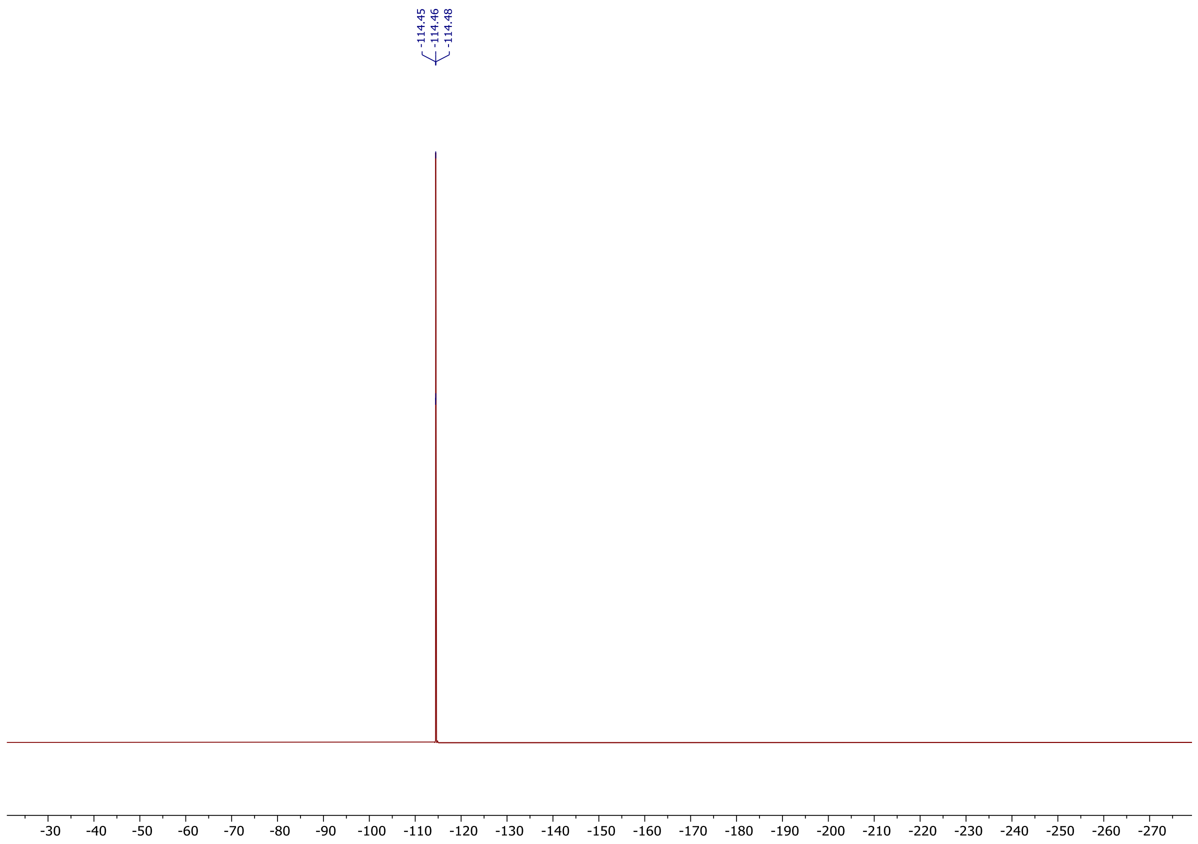
-114.45
-114.46
-114.48
-30 -40 -50 -60 -70 -80 -90 -100 -110 -120 -130 -140 -150 -160 -170 -180 -190 -200 -210 -220 -230 -240 -250 -260 -270

4-Chloro-*N*-(2-(2-(prop-2-yn-1-yloxy)ethoxy)ethyl)pyrimidin-2-amine S2

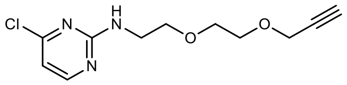

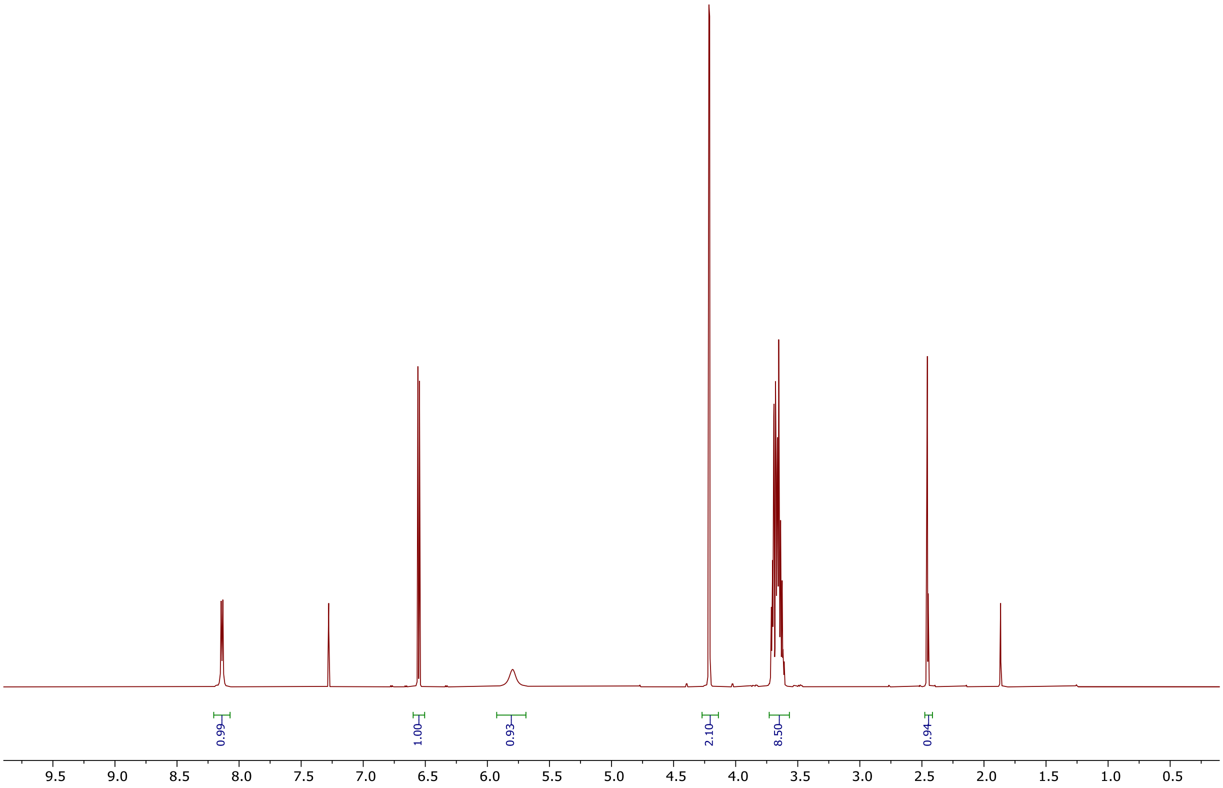

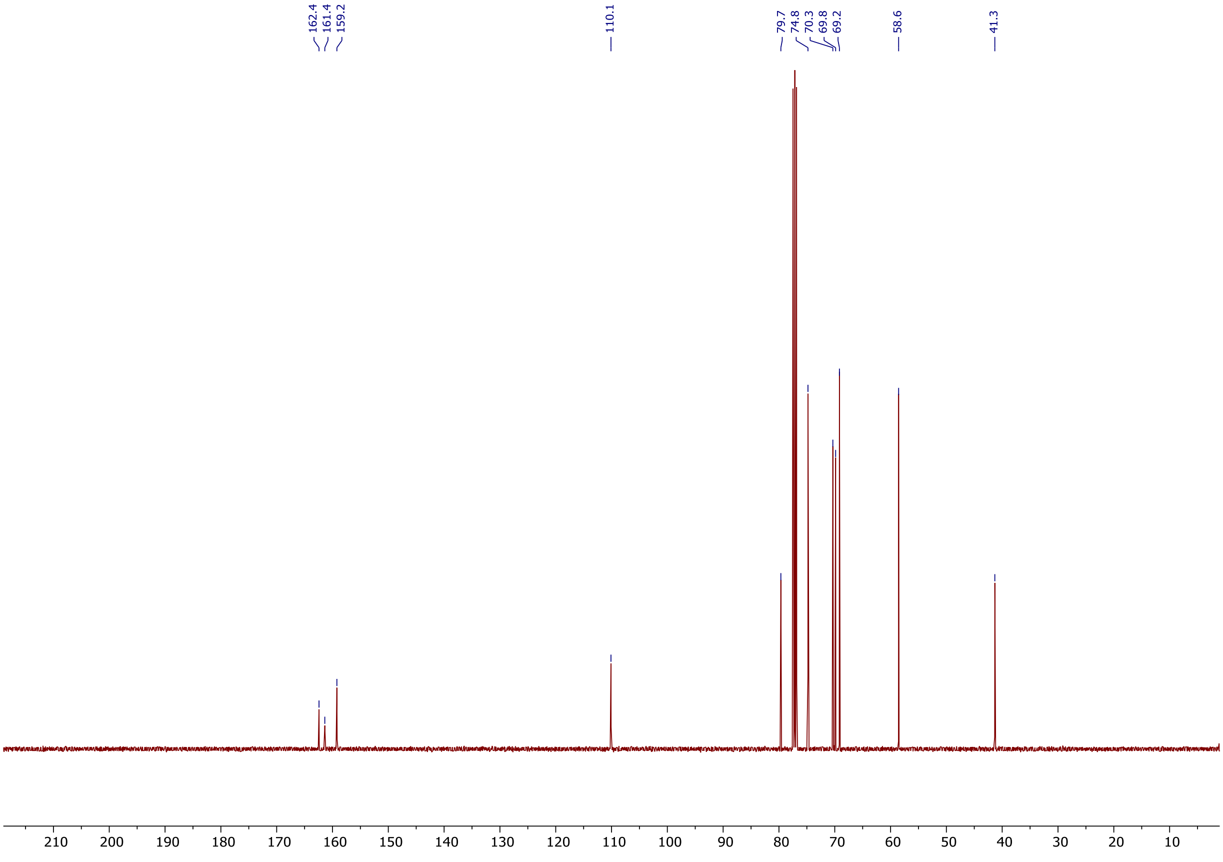

N-(2-(2-(Prop-2-yn-1-yloxy)ethoxy)ethyl)-4-vinylpyrimidin-2-amine 8

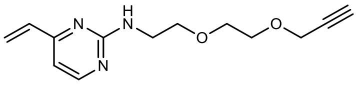

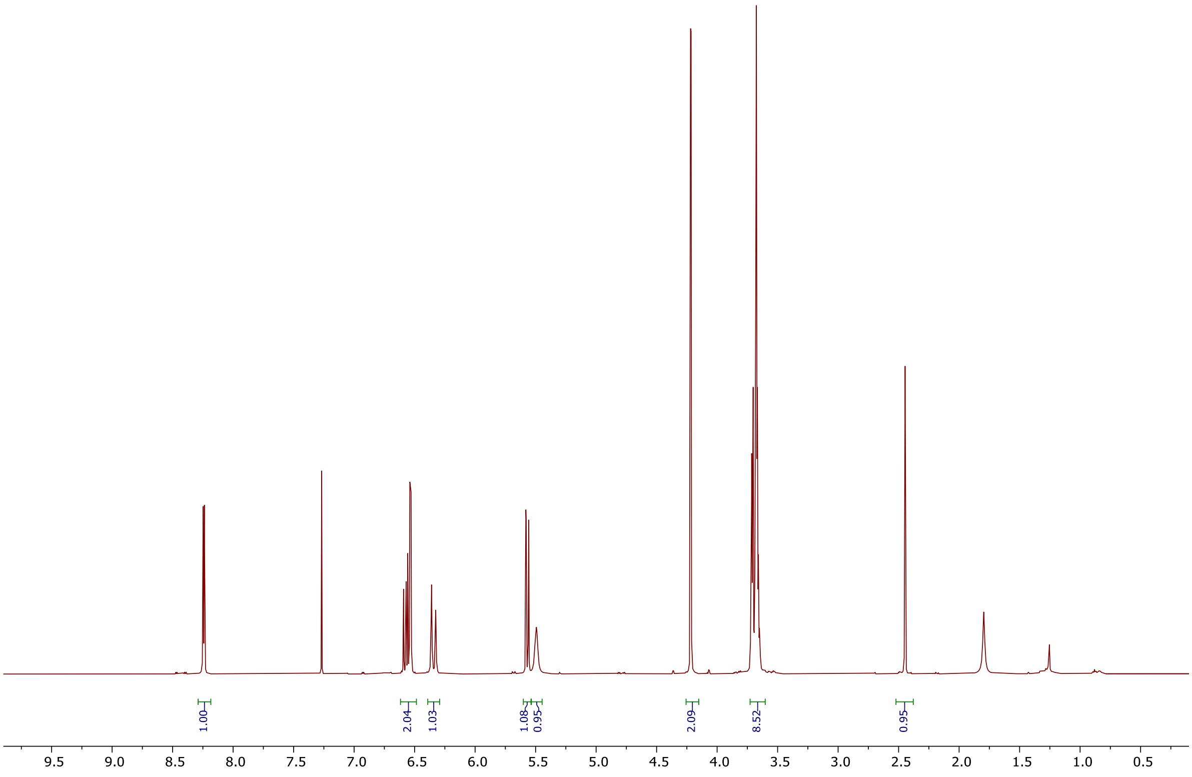

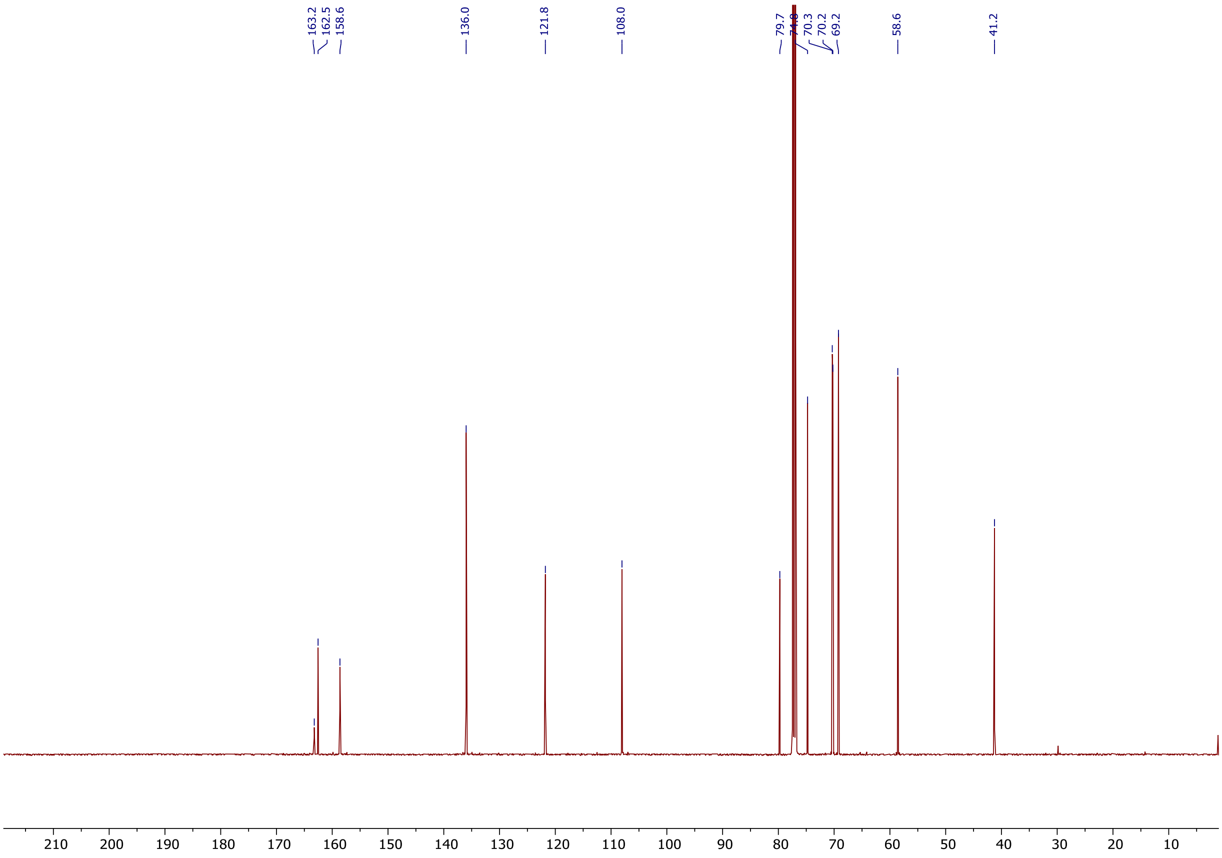

N-(2-(2-(2-(2-Azidoethoxy)ethoxy)ethoxy)ethyl)-7-nitrobenzo[c][1,2,5]oxadiazol-4-amine

S3

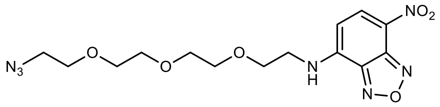

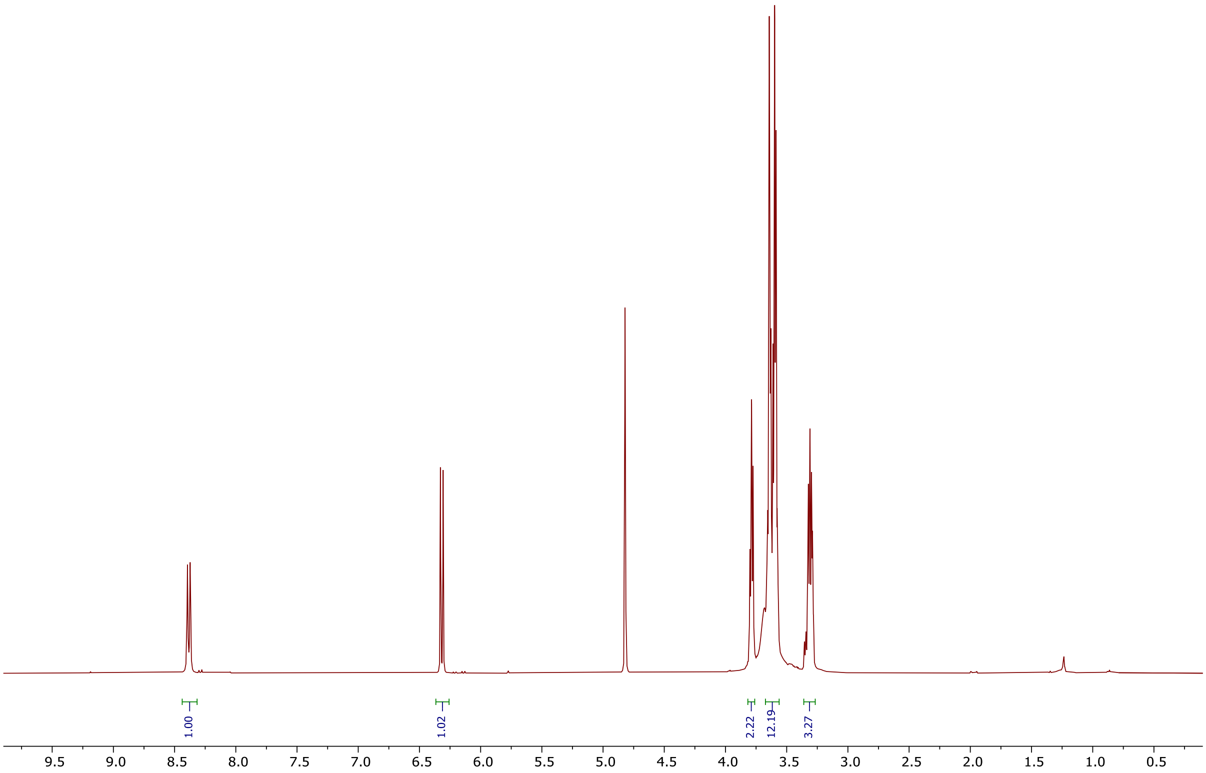

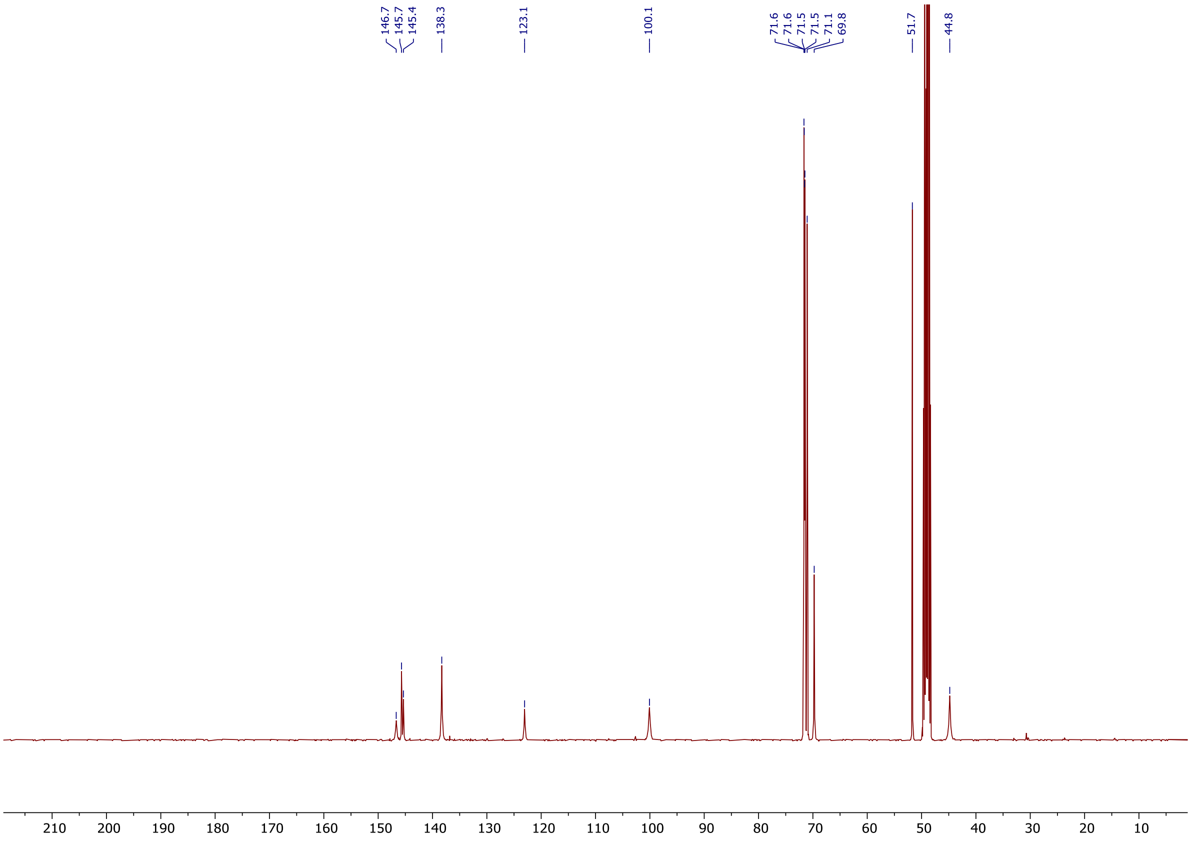

7-Nitro-*N*-(2-(2-(2-(2-(4-((2-(2-((4-vinylpyrimidin-2-yl)amino)ethoxy)ethoxy)methyl)-1*H*-1,2,3-triazol-1-yl)ethoxy)ethoxy)ethoxy)ethyl)benzo[*c*][1,2,5]oxadiazol-4-amine 9

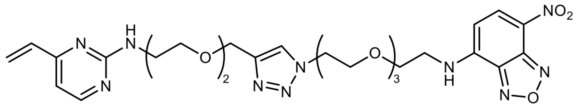

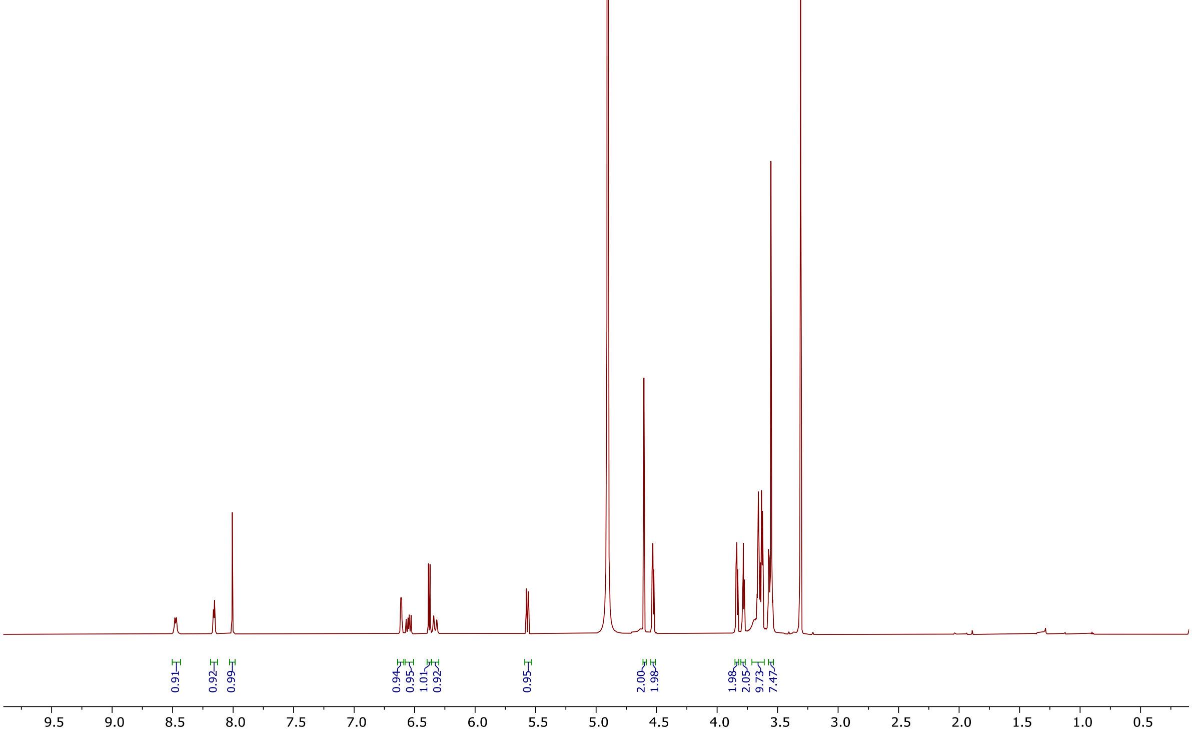

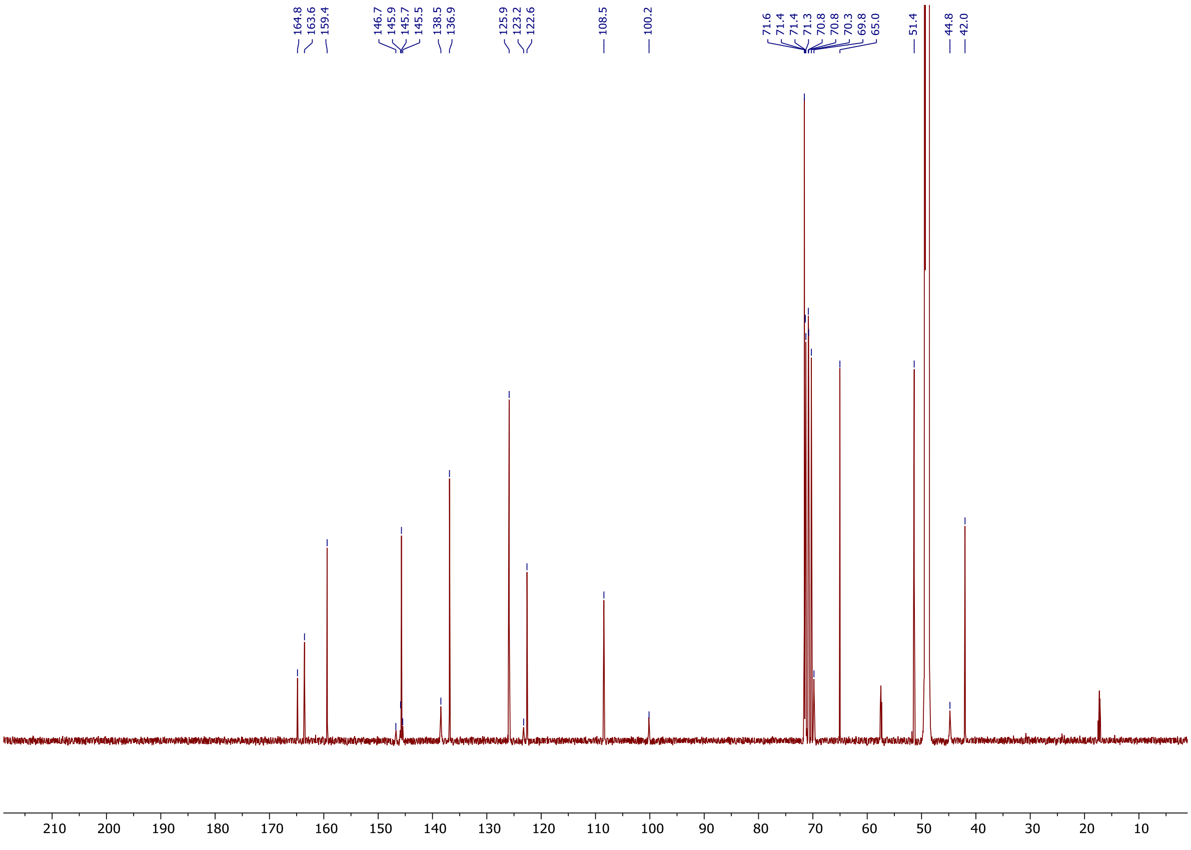

***N*-(2-(2-(2-(2-azidoethoxy)ethoxy)ethoxy)ethyl)-5-((3a*R*,4*R*,6a*S*)-2-oxohexahydro-1*H*-thieno[3,4-d]imidazol-4-yl)pentanamide 12**

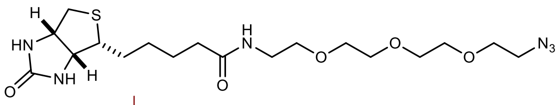

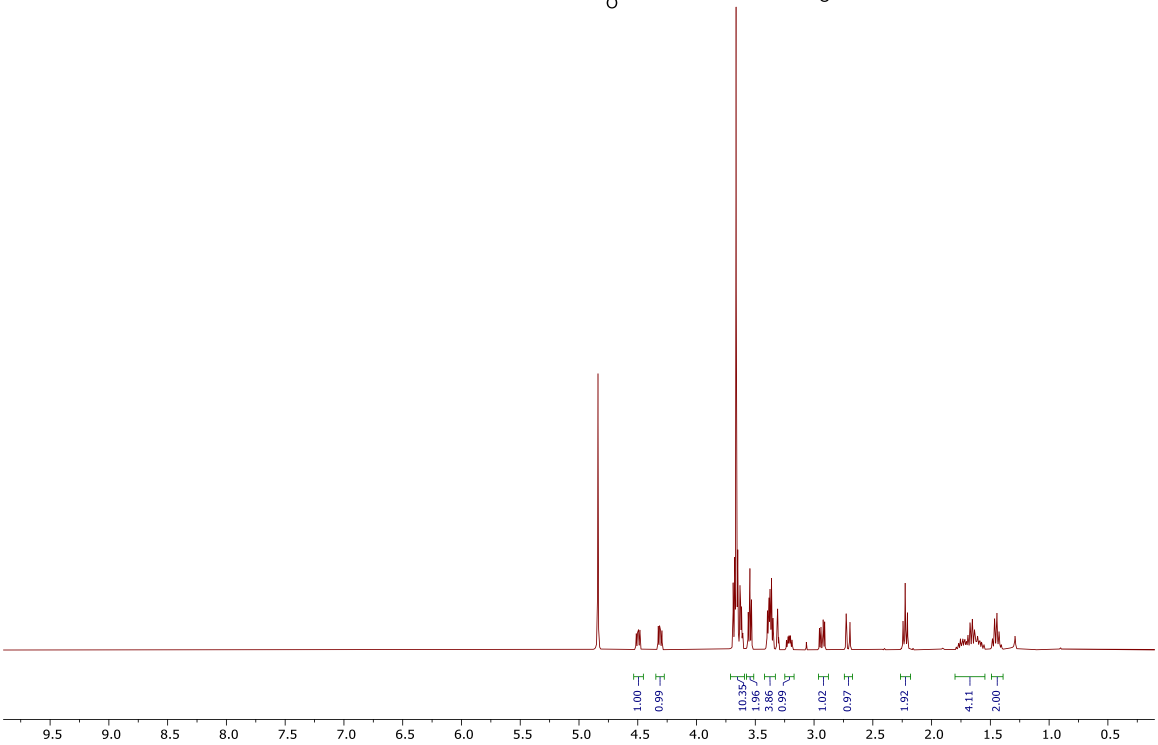

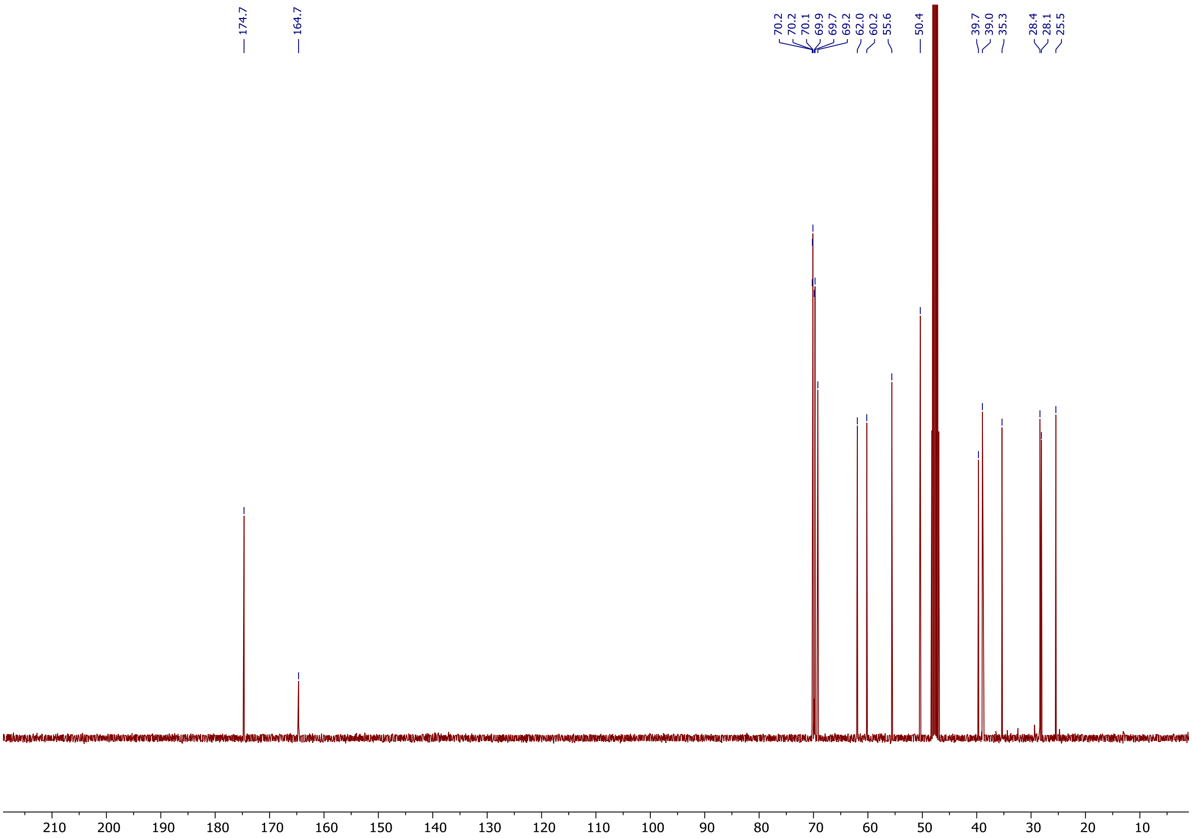

5-((3a*S*,4*S*,6a*R*)-2-Oxohexahydro-1*H*-thieno[3,4-*d*]imidazol-4-yl)-N-(2-(2-(2-(2-(4-((2-(2-((4-vinylpyrimidin-2-yl)amino)ethoxy)ethoxy)methyl)-1*H*-1,2,3-triazol-1-yl)ethoxy)ethoxy)ethoxy)ethyl)pentanamide 10

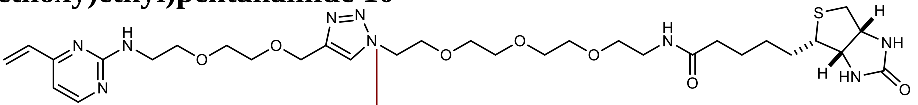

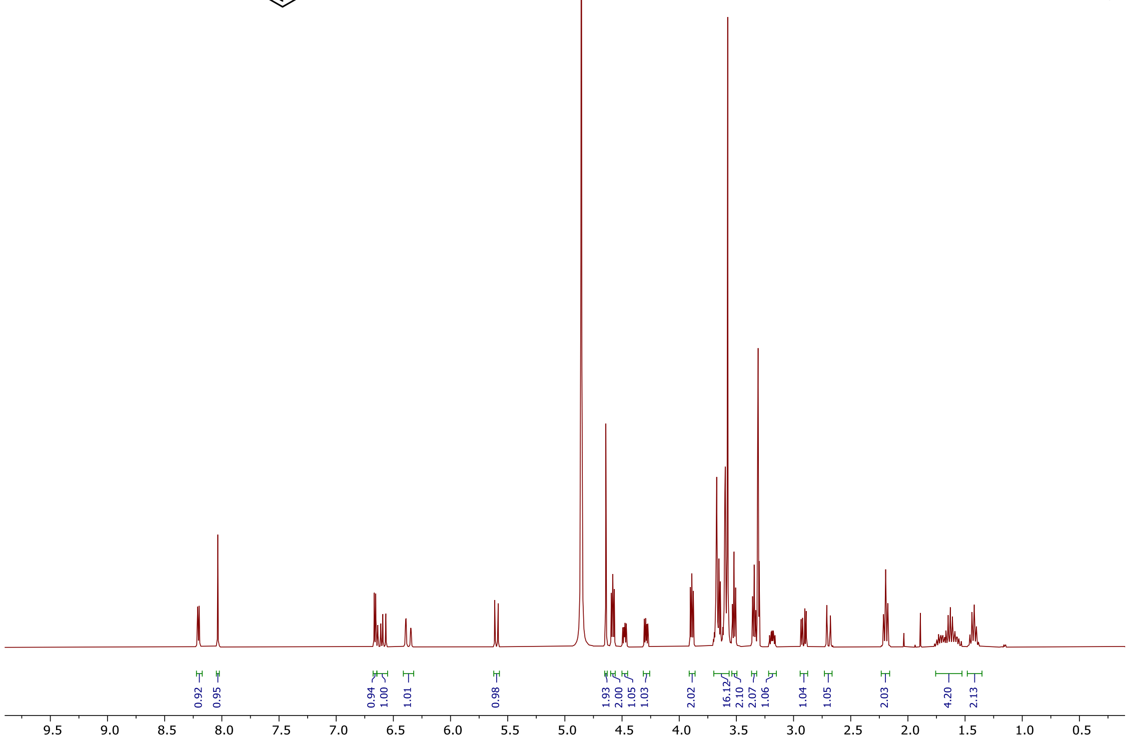

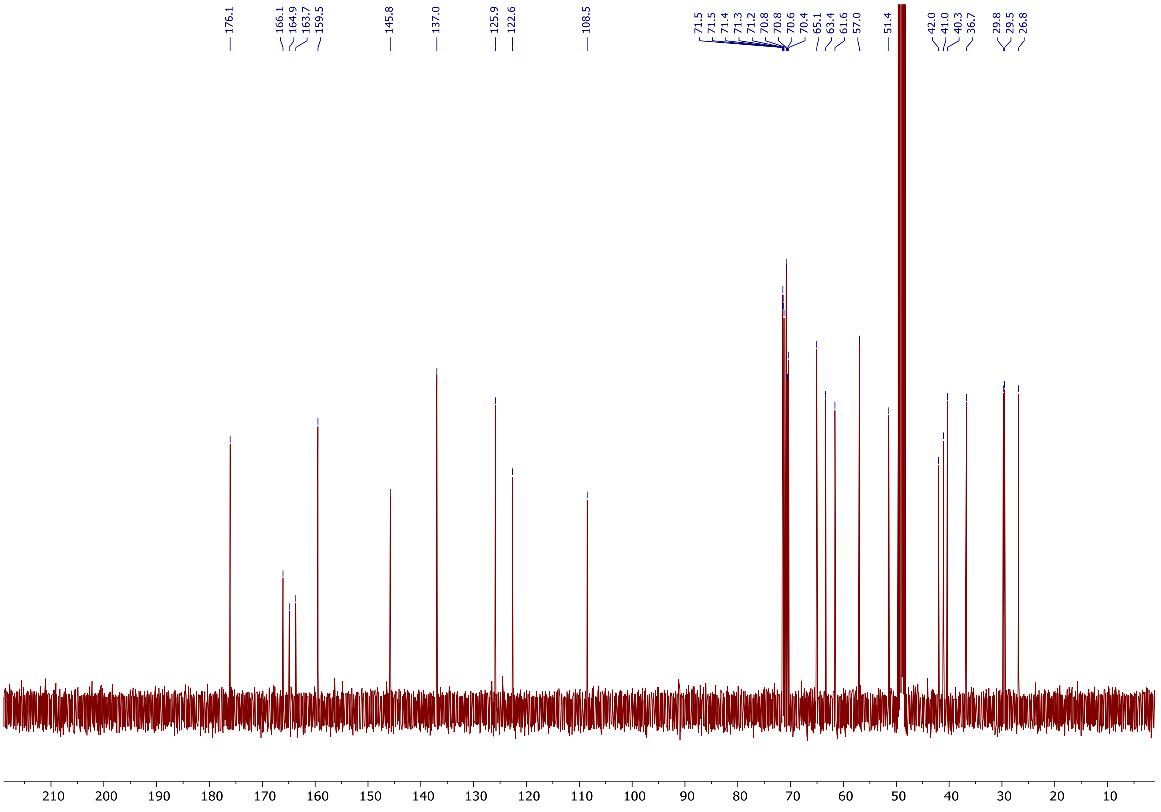

4-Chloro-*N*-(2-(2-(prop-2-yn-1-yloxy)ethoxy)ethyl)-1,3,5-triazin-2-amine S4

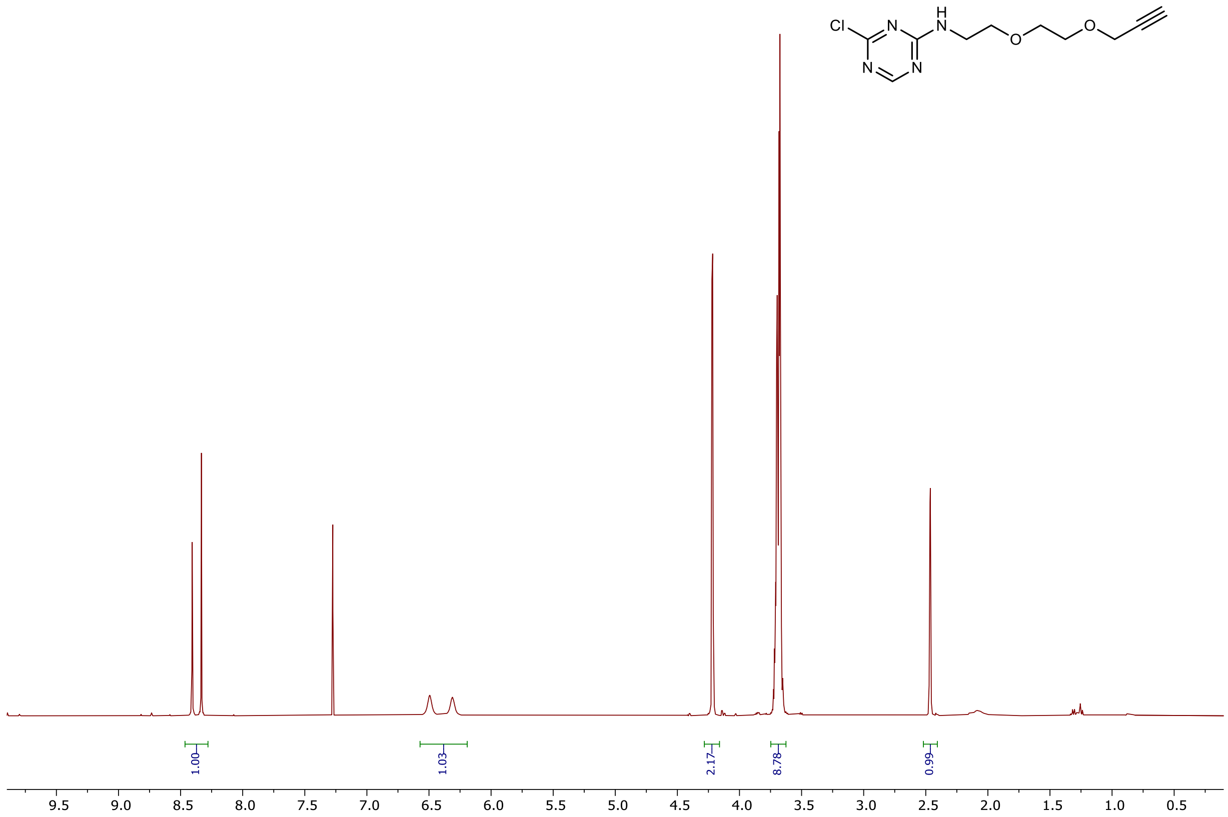

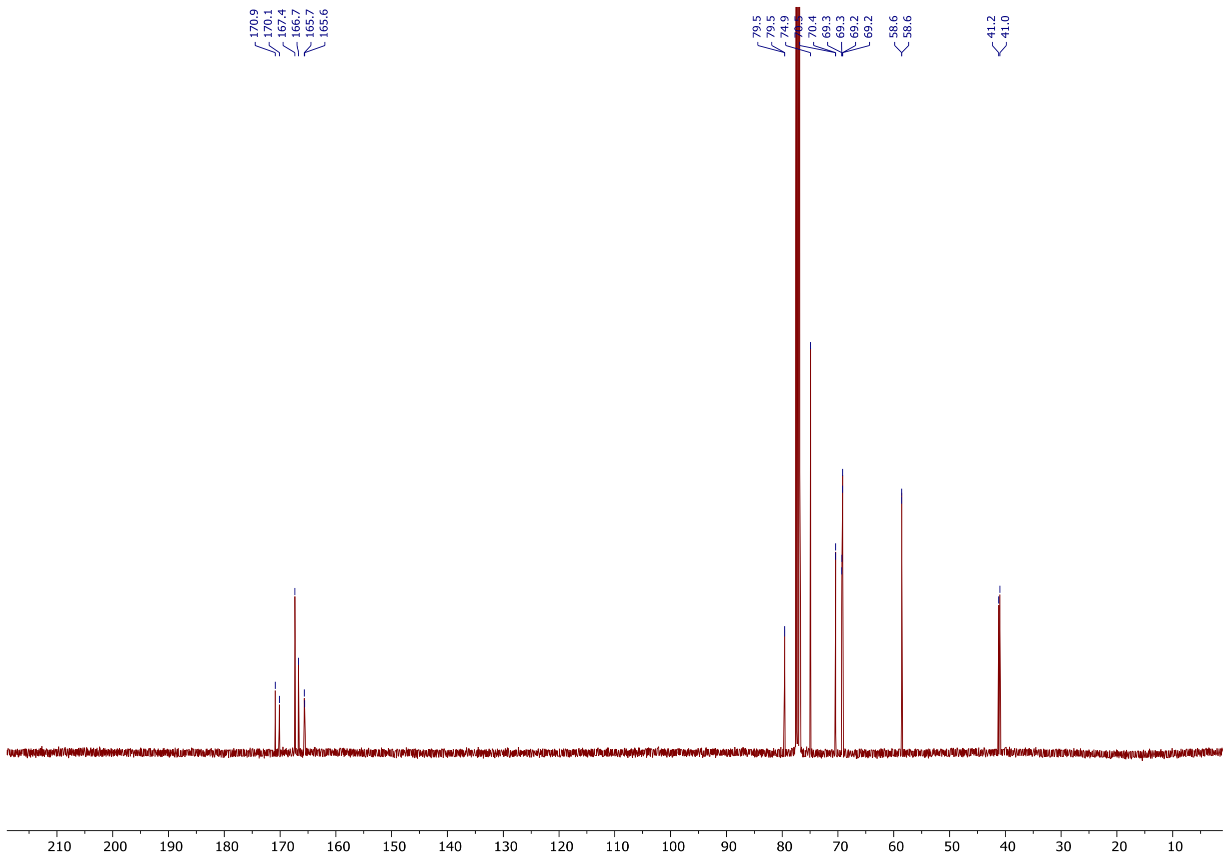

N-(2-(2-(Prop-2-yn-1-yloxy)ethoxy)ethyl)-4-vinyl-1,3,5-triazin-2-amine 11

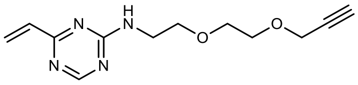

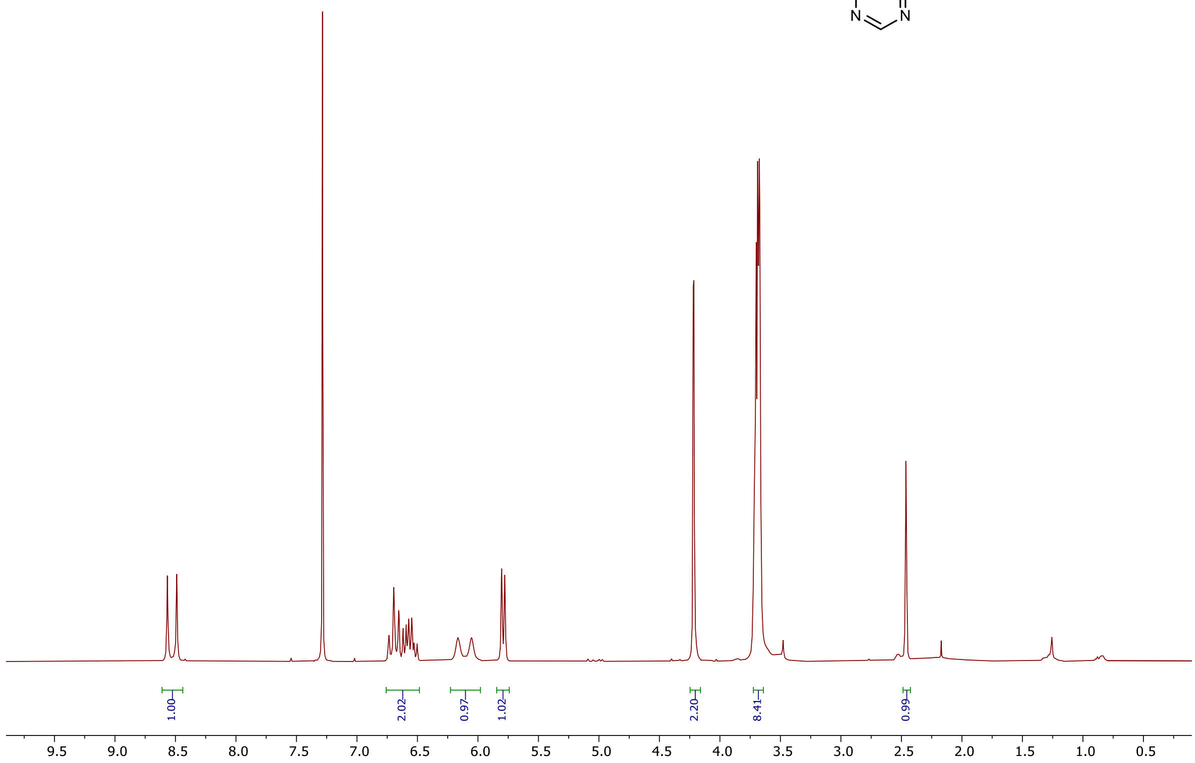

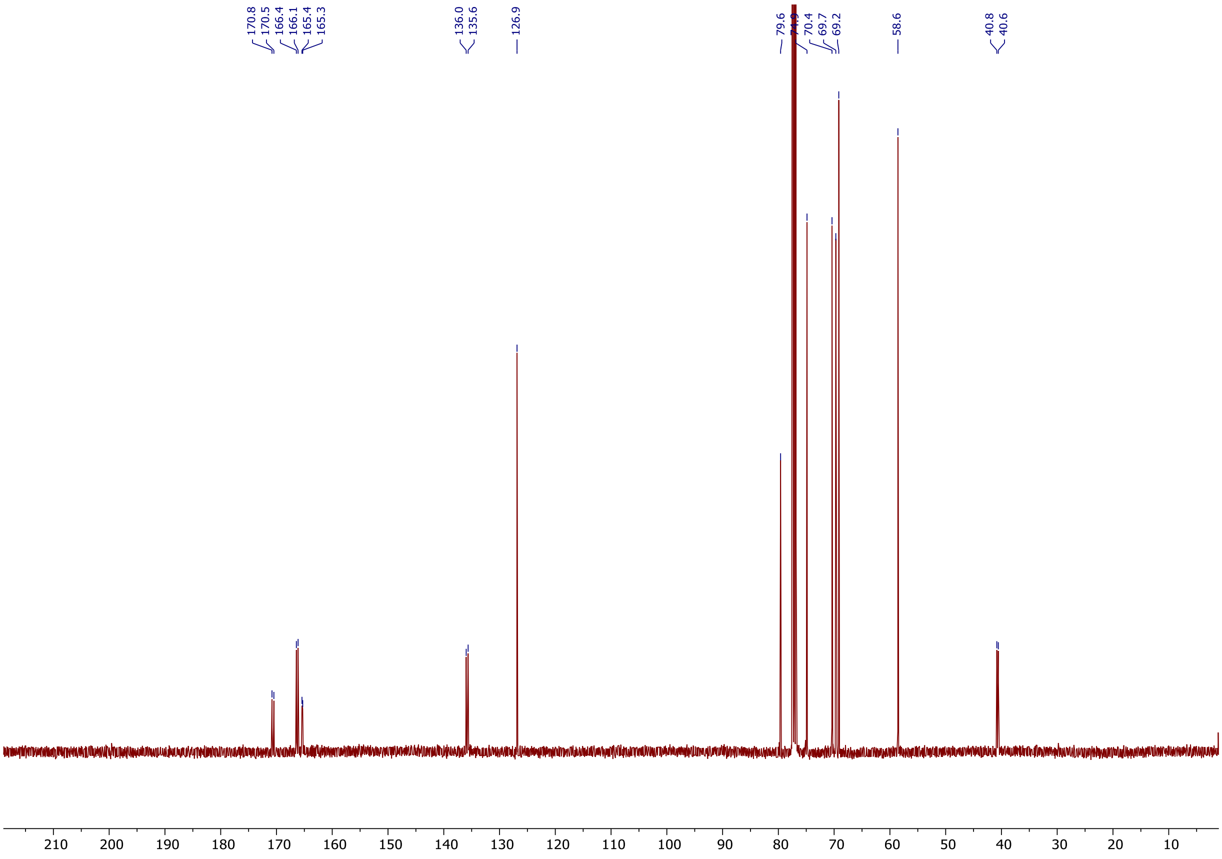

1-(2-(2-(Prop-2-yn-1-yloxy)ethoxy)ethyl)-1*H*-pyrrole-2,5-dione 15

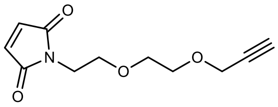

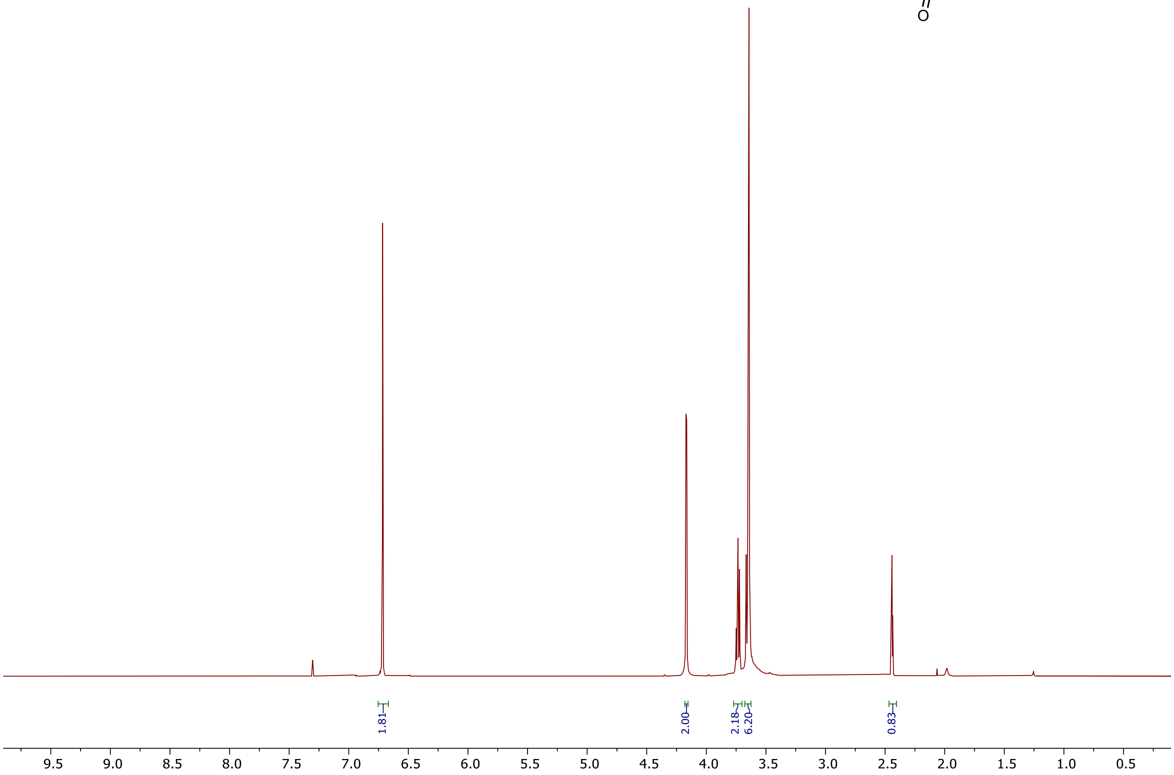

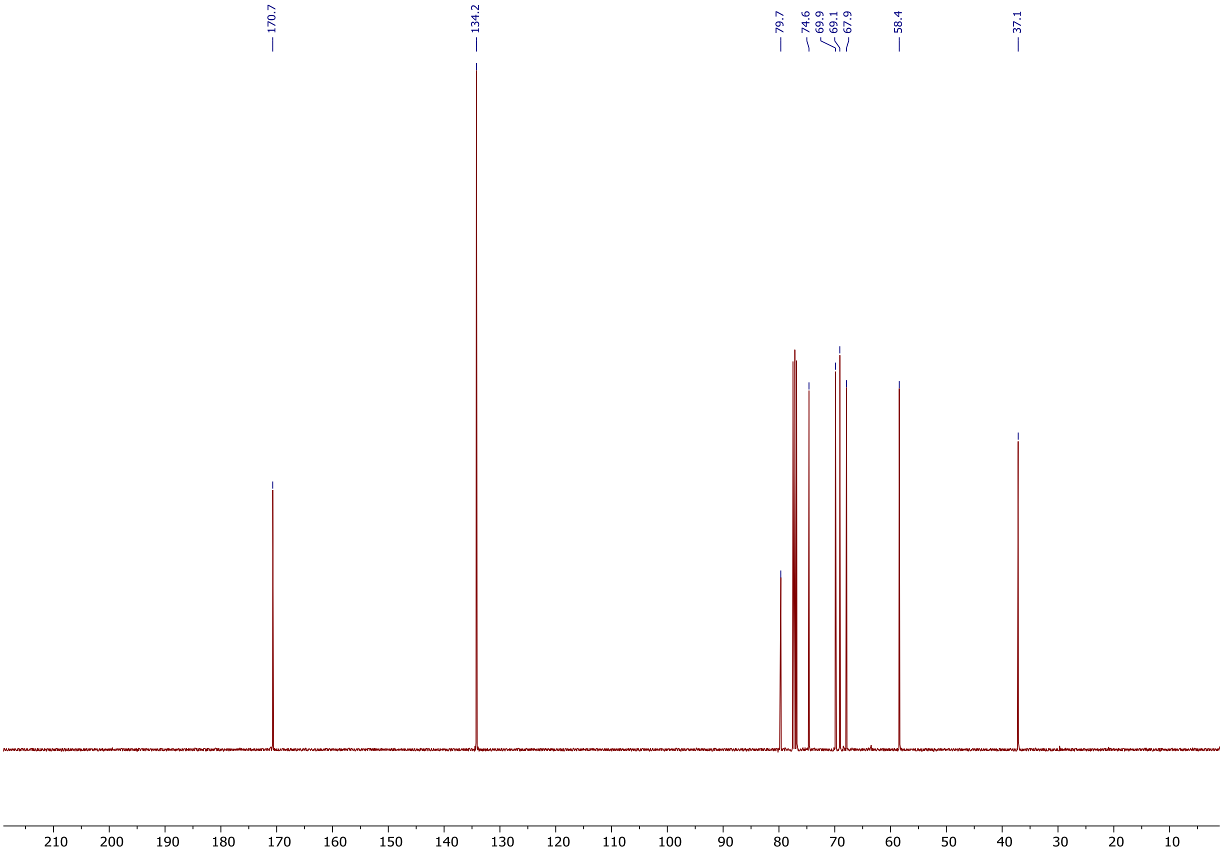

11. HPLC Traces

Azide-PEG3-arylsulfate-MMAE 14

Absorbance at 254 nm. Solvent system: 5-95% MeCN/H_2O over 20 min.

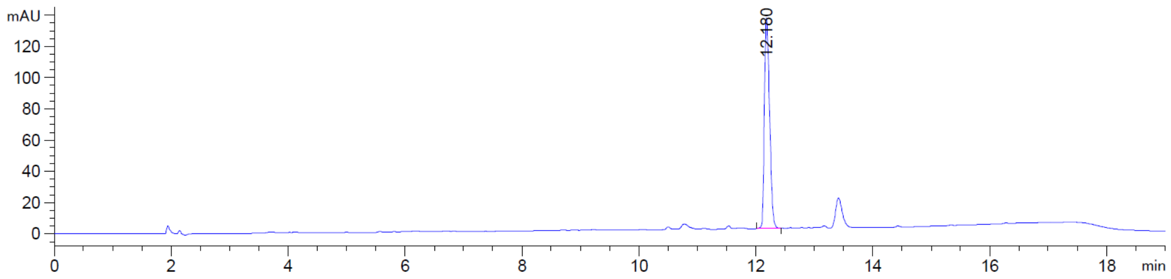

Supplement: SC-012-D1SC02722K-s001 [file SC-012-D1SC02722K-s001.pdf]
